# Analysis of shared pathogenic mechanisms and drug targets in myocardial infarction and gastric cancer based on transcriptomics and machine learning

**DOI:** 10.3389/fimmu.2025.1533959

**Published:** 2025-03-21

**Authors:** Junyang Ma, Shufu Hou, Xinxin Gu, Peng Guo, Jiankang Zhu

**Affiliations:** ^1^ School of Clinical Medicine, Jining Medical University, Jining, China; ^2^ Laboratory of Metabolism and Gastrointestinal Tumor, The First Affiliated Hospital of Shandong First Medical University, Jinan, China; ^3^ Jinzhou Medical University, Shanghai Fengxian District Central Hospital Postgraduate Training Base, Shanghai, China; ^4^ College of Clinical and Basic Medicine, Shandong First Medical University, Jinan, China

**Keywords:** gastric cancer, myocardial infarction, Mendelian randomization, machine learning, transcriptomics

## Abstract

**Background:**

Recent studies have suggested a potential association between gastric cancer (GC) and myocardial infarction (MI), with shared pathogenic factors. This study aimed to identify these common factors and potential pharmacologic targets.

**Methods:**

Data from the IEU Open GWAS project were used. Two-sample Mendelian randomization (MR) analysis was used to explore the causal link between MI and GC. Transcriptome analysis identified common differentially expressed genes, followed by enrichment analysis. Drug target MR analysis and eQTLs validated these associations with GC, and the Steiger direction test confirmed their direction. The random forest and Lasso algorithms were used to identify genes with diagnostic value, leading to nomogram construction. The performance of the model was evaluated via ROC, calibration, and decision curves. Correlations between diagnostic genes and immune cell infiltration were analyzed.

**Results:**

MI was linked to increased GC risk (*OR*=1.112, *P*=0.04). Seventy-four genes, which are related mainly to ubiquitin-dependent proteasome pathways, were commonly differentially expressed between MI and GC. Nine genes were consistently associated with GC, and eight had diagnostic value. The nomogram built on these eight genes had strong predictive performance (*AUC*=0.950, validation set *AUC*=0.957). Immune cell infiltration analysis revealed significant correlations between several genes and immune cells, such as T cells, macrophages, neutrophils, B cells, and dendritic cells.

**Conclusion:**

MI is associated with an increased risk of developing GC, and both share common pathogenic factors. The nomogram constructed based on 8 genes with diagnostic value had good predictive performance.

## 1 Introduction

Gastric cancer (GC) remains one of the leading causes of cancer-related deaths worldwide. Although the incidence of GC has declined in some regions of the world, data from the past decade show that the incidence of GC has remained high and has even increased in specific regions and populations (1, 2). By 2040, the global burden of this malignant tumor is projected to increase by 62% (3). The treatment of GC is mainly radical surgery supplemented with radiotherapy, chemotherapy, and drug therapy. However, research on the treatment of GC is still in progress, to further clarify the pathogenesis of GC, identify the risk factors for GC, and overcome the challenges in the treatment of GC (1, 4–6).

Myocardial infarction (MI), or heart attack, is a leading cause of morbidity and mortality worldwide. It typically results from coronary artery disease, where thrombotic occlusion of an artery or bypass graft leads to a sudden reduction or complete interruption of myocardial blood supply, potentially causing heart failure or death (7–9). Established risk factors such as diabetes, smoking, obesity, hypertension, and hyperlipidemia significantly contribute to MI occurrence, while sex, aging, genetic predisposition, family history of cardiovascular disease, and racial differences also play a role (10, 11). Moreover, emerging evidence suggests that systemic diseases, particularly cancer, may worsen MI progression or elevate its risk, highlighting the need for further investigation into the potential link between cancer and MI (12).

Although they belong to different systems, GC and MI share many similar pathogenic factors and may be potentially interrelated. Examples include inflammation (7, 13), a high-salt diet (14, 15), and smoking (16, 17). In addition, in some cases, GC and MI may present similar clinical features. These include pain in the upper abdomen or chest and dyspepsia, nausea, and vomiting (4, 18, 19). However, little is known about the complex mechanisms underlying the relationship between GC and MI, and the available research evidence is limited. A long-term cohort study suggested that MI may increase the risk of cancer (20), and a large meta-analysis revealed that *Helicobacter pylori* infection, one of the main causative factors of GC, was also associated with an increased risk of MI (21). Given these findings, exploring the common pathogenic mechanisms of GC and MI has become an important research direction. However, traditional observational studies have limitations in inferring causality (22), and although associations between GC and MI can be observed, determining the causal relationship between them is not yet possible. Therefore, this study aims to further explore the common pathogenic mechanisms of these two diseases through a more rigorous study design and to promote the progress of related diagnostic and treatment strategies.

In recent years, Mendelian randomization (MR) analysis has gradually become a powerful tool in the fields of drug target discovery and drug repurposing (23). In MR studies, researchers utilize genetic variants associated with specific exposures as instrumental variables (IVs) to assess the causal links between exposures and outcomes. Compared with traditional observational studies, MR analyses can effectively avoid the interference of confounding factors such as acquired environmental factors and behavioral habits and significantly reduce the influence of reverse causality (24, 25). This approach is similar to randomized controlled trials in nature, where genetic variation plays a role similar to that of randomized grouping, thus providing a reliable basis for drug target validation (23). With the rapid development of genome-wide association studies (GWASs), MR strategies have led to breakthroughs in therapeutic target identification for a variety of diseases (26, 27). In addition, MR analysis of drug targets can be used to predict the pharmacological modulatory effects of drug targets, simulate drug responses in clinical trials, and predict the potential benefits and risks of treatment (28, 29).

In this study, we innovatively combined MR analysis and transcriptome analysis to investigate the association between GC and MI and explored the potential MI-related therapeutic targets of GC through drug target MR and machine learning. These findings provide insights for understanding the common pathogenesis of GC and MI.

## 2 Method

### 2.1 Study design

**Figure 1:**
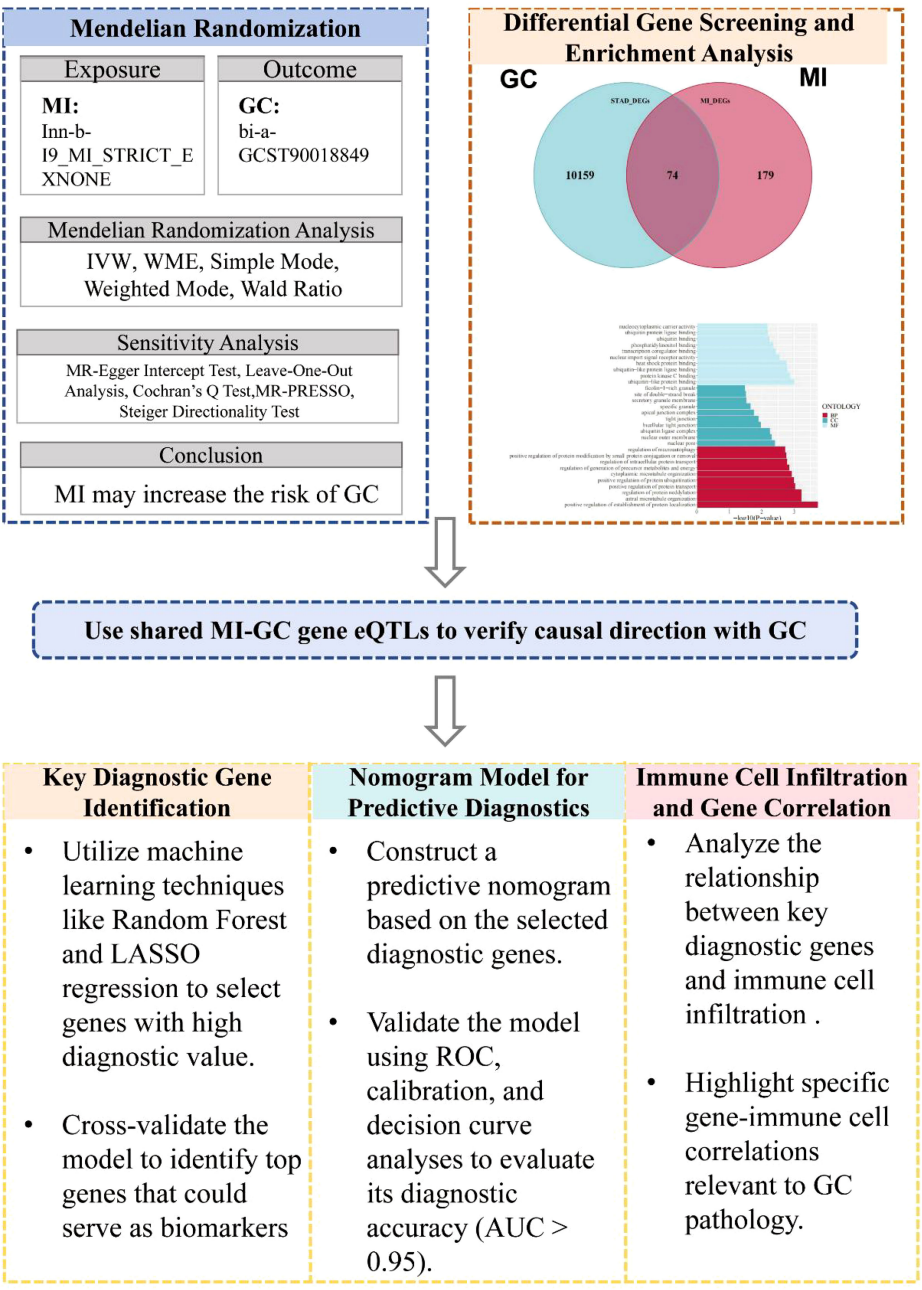
Overview of study design. MR, Mendelian Randomization; GC, Gastric Cancer; MI, Myocardial Infarction; IVW, Inverse-Variance Weighted; WME, Weighted Median Estimator; eQTLs, Expression Quantitative Trait Loci; ROC, Receiver Operating Characteristic; AUC, Area Under the Curve.

In this study, we first explored the causal relationship between MI and GC via the MR method (see **Figure 1**). Differentially expressed genes associated with MI and GC were subsequently screened, and these genes were enriched and analyzed. Next, the expression quantitative trait loci (eQTLs) of the common differential genes of MI and GC were utilized to explore the causal associations between these genes and GC and to verify whether the direction of causality was as expected. Finally, to identify candidate biomarkers and construct a prediction model for GC, this study used machine learning algorithms, such as random forest and least absolute shrinkage and selection operator (LASSO) regression, to screen key genes for the construction of a column-line graph (nomogram) and verified its performance in the diagnosis and prediction of GC. In addition, we explored the correlations between genes with diagnostic value and immune cell infiltration.

The MR analysis method used in this study is consistent with the three main hypotheses of MR research: (1) IVs are associated with risk factors. (2) IVs are not associated with confounding factors. (3) IVs affect the outcome only through risk factors (30, 31) with the STRIOBE-MR guidelines (32) (**Figure 1**).

### 2.2 Data sources

GWAS data for MI (finn-b-I9_MI_STRICT_EXNONE), GC (ebi-a-GCST90018849), and differential gene expression quantitative trait loci (eQTLs) were obtained from the IEU Open GWAS project (https://gwas.mrcieu.ac.uk/). The data obtained were derived from European populations, for which summary information is given in **Table 1**. The relevant data were obtained from publicly available GWAS databases; therefore, this part of the study did not address the need for ethics committee approval.

**Table 1:** Brief information on the GWAS database in the MR study.

Data source	Phenotype	Sample size	Cases	Population	Adjustment
IEU Open GWAS project(finn-b-I9_MI_STRICT_EXNONE)	MI	218792	11622	European	Males and Females
IEU Open GWAS project(ebi-a-GCST90018849)	GC	476116	1029	European	–

#### 2.2.1 Instrumental variable screening conditions for MI and GC

Significant SNPs were screened from pooled GWAS data for MI. The linkage disequilibrium coefficient (r2) was set to 0.001, and the width of the linkage disequilibrium region was set to 10,000 kb to ensure that the individual SNPs were independent of each other and to exclude the influence of genetic pleiotropy on the results. LDtrait (https://ldlink.nih.gov/?tab=ldtrait) was used to exclude SNPs associated with confounders and outcomes. The relevant SNPs screened above were extracted from the GWAS pooled data of GC; the minimum *r^2^* was set to > 0.8 (33, 34). The instrumental variable screening condition was *P* < 1 × 10^-5^, *F* > 10 was set to reduce weak instrumental variable bias, and a nonzero intercept term in the MR−Egger regression model (*P* > 0.05) indicated that there was no genetic pleiotropy in the SNPs.

#### 2.2.2 Differential gene expression quantitative trait loci and GC instrumental variable screening conditions

The chain disequilibrium coefficient (r2) was set to 0.3, the width of the chain disequilibrium region was 300 kb, and the minor allele frequency (*MAF*) was >0.01 to ensure that the individual SNPs were independent of each other and to eliminate the influence of chain disequilibrium on the results, including the SNPs associated with confounders and the SNPs associated with endings. Instrumental variables located within ±300 kb from the cis-acting region of the drug target gene were extracted from the eQTL data of the drug target genes screened above for relevant instrumental variables (35, 36). In addition, the above screened relevant SNPs were extracted from the GWAS summary data of the outcome variable GC, excluding SNPs with palindromic structure and an *MAF*>0.42, excluding SNPs directly associated with the outcome variable (*P*<1×10^-5^), and excluding abnormal SNPs via MR-PRESSO.

### 2.3 MR analysis methods

Two-sample MR and drug-target MR analyses were performed via five regression models, namely, MR−Egger regression, random-effects inverse-variance weighted (IVW), weighted median estimator (WME), weighted model, and simple model, and two-sample MR analyses were applied to assess the potential causal relationship between MI and GC risk. Inverse variance weighting (IVW) was used as the main method for causal estimation. When SNPs ≤ 3, the effect of a single SNP on the outcome was applied to the Wald coefficient ratio method (Wald ratio), and the rest were applied to the fixed-effects IVW method; when more than 3 SNPs were included, the random-effects IVW method was applied (37–39). MR−Egger essentially uses a weaker hypothetical premise (InSIDE) based on IVW to accomplish causal effect estimation and detects and corrects for bias due to instrumental variable multivariate polytomies by introducing a regression intercept to estimate the causal relationship between the exposure and the outcome in the presence of horizontal polytomies. The results of MR−Egger are referred to when there are horizontal polytomies. The Cochran's Q test and the I² (I-squared) statistic were used to determine the heterogeneity of the SNPs, which were heterogeneous if the Cochran's Q test had a *P* < 0.05 (40). The values of I² ranged from 0% to 100%, with an I² greater than 50%, indicating that the SNPs had some heterogeneity (41). The intercept term of the MR−Egger method was used for polytropy analysis, and leave-one-out was used for sensitivity analysis. A nonzero intercept term (*P* > 0.05) in the MR−Egger regression model indicated that the SNPs were not polytropic (42). Leave-one-out analyses were performed on the SNPs by progressively removing each SNP and reanalyzing the remaining SNPs to observe the magnitude of the effect of each SNP on the analysis results (43, 44). All of the above methods were implemented via the two-sample MR package in R 4.1.0 software with a test level of *α* = 0.05.

### 2.4 MI and GC differential gene screening

Gene expression data were obtained from the Gene Expression Omnibus (GEO) database (http://www.ncbi.nlm.nih.gov/geo/) and The Cancer Genome Atlas (TCGA). Differential expression analysis was performed on GC tissues (375 cases) and normal gastric tissues (32 cases) via limma analysis, and differential expression analysis was performed on MI patient samples (17 cases) and control samples (20 cases) from the GEO dataset (GSE83500), and intersections were taken for the differential genes obtained (45).

### 2.5 MI and GC differential gene acquisition and gene set enrichment analysis

To further confirm the functions of potential targets, the data were explored via functional enrichment analysis. Gene Ontology (GO) analysis was used to examine and clarify coordinated changes at the pathway level and gene function between phenotypes. The focus of GO analysis is to identify differences in biological processes, cellular components, and molecular functions to help reveal potential biological functions.

### 2.6 Steiger direction test

To verify the consistency of genotypes in the direction of causality between intermediate variables (gene expression) and the outcome (GC and MI), the Steiger direction test was used in this study. The causal direction was determined by calculating the explained variance of the instrumental variables (eQTLs) on the intermediate and outcome variables, and the direction was considered to be correct if the explained variance of the instrumental variables on the intermediate variables was greater than the explained variance on the outcome. Analyses were performed via the TwoSampleMR package in R. The significance level was set at *α* = 0.05, and directional consistency was judged by “TRUE” or “FALSE” in the test results.

### 2.7 Screening of genes with diagnostic value and GC prognostic modeling

To identify candidate biomarkers and build a prediction model for GC, a variety of random forest and LASSO regression algorithms were used to screen key genes. The random forest algorithm is an integrated learning algorithm that improves prediction accuracy by constructing multiple decision trees and combining their results and is particularly suitable for handling high-dimensional data, assessing the importance of features, and reducing overfitting. LASSO regression performs L1 regularization for variable selection and model simplification, which is based on the principle of applying penalties to the regression coefficients and reducing unimportant feature coefficients to zero, thus improving the generalization ability and interpretability of the model. Based on the screened genes with diagnostic value, a nomogram was constructed via the R package “rms”, and the area under the ROC curve was plotted to evaluate the diagnostic effect of the genes with diagnostic value in GC diagnosis. Finally, calibration curve and decision curve analysis (DCA) were performed to evaluate the efficiency of the predictive model of GC predicted by the nomogram.

### 2.8 Correlation analysis between genes with diagnostic value and immune cell infiltration

The gene expression matrix of GC was uploaded to the CIBERSORTx database (https://cibersortx.stanford.edu/), and immune cell infiltration was calculated for each sample. Correlation analysis between key genes and immune cell infiltration was performed via Spearman rank correlation coefficients.

## 3 Result

### 3.1 MI as a risk factor for GC incidence

**Figure 2:**
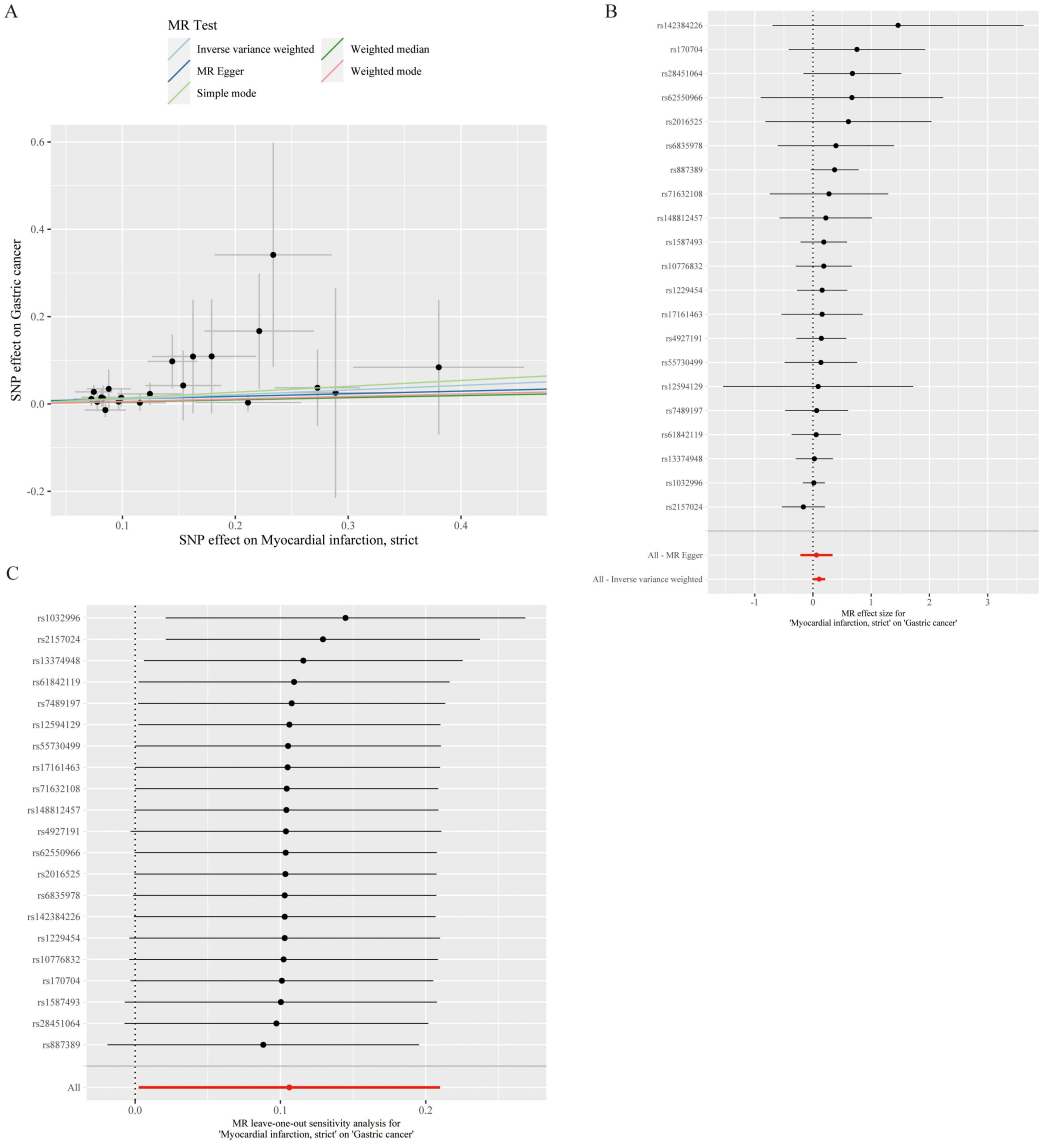
Causal relationship analysis of myocardial infarction on gastric cancer risk. **(A)** Scatter Plot: The slope of each line represents the effect estimated through different Mendelian randomization methods. **(B)** Forest Plot: Red dots indicate the combined estimate after integrating all SNPs using the Inverse Variance Weighted (IVW) method, with horizontal lines representing the 95% confidence intervals. **(C)** Leave-One-Out Analysis: Black dots represent the causal effect estimated by the IVW method while excluding a specific variant, and red dots indicate the IVW estimate using all SNPs.

After screening and the strict quality control described above, 21 SNPs were ultimately included for MR analysis, and the basic information of the SNPs is shown in **Table 2**. After MR, the IVW results revealed that MI was associated with an increased risk of developing GC (*OR*=1.112, 95% *CI*: 1.002–1.233, *P*=0.045) (**Figure 2A**). MI heterogeneity was not detected among eQTLs associated with GC (*I²*= 0%, *Cochran's Q*=11.199, *P*=0.941). The MR−Egger results revealed no statistically significant difference between the intercept term and 0 (*P*=0. 724), and MR−PRESSO did not detect significant horizontal pleiotropy (*P*=0.929). Therefore, no horizontal pleiotropy occurred in the SNPs (**Table 3**). Scatter and funnel plots for GC revealed that the distributions of all the included SNPs were largely symmetrical, indicating that causal associations were less likely to be affected by potential bias (**Figures 2B, C**). After each SNP of GC was excluded sequentially via the leave-one-out test, the analysis results of the remaining SNPs were similar to those with the inclusion of all the SNPs (**Figure 2D**), and no SNPs were found to have a large impact on the estimation value of the causal associations, indicating that the MR results of the present study were robust.

**Table 2:** Information on single nucleotide polymorphisms (SNPs) associated with MI and GC.

SNP	Outcome	CHR	POS	EA/OA	*EAF*	β*	*SE*	*P*	*F-statistic*
MI									
rs2016525	GC	1	174355957	G/A	0.031	0.109	0.130	0.402	20.536
rs62550966	GC	9	91397911	T/C	0.029	0.109	0.130	0.403	19.930
rs28451064	GC	21	35593827	A/G	0.103	0.098	0.062	0.113	42.903
rs148812457	GC	1	17890801	A/G	0.024	0.084	0.154	0.585	25.238
rs71632108	GC	1	181267101	C/T	0.086	0.042	0.080	0.596	20.952
rs55730499	GC	6	161005610	T/C	0.071	0.037	0.087	0.669	51.810
rs887389	GC	17	3793303	G/A	0.538	0.028	0.016	0.078	20.441
rs10776832	GC	9	138495074	T/C	0.746	0.015	0.020	0.447	20.861
rs13374948	GC	1	228235008	G/A	0.147	-0.003	0.019	0.882	25.174
rs1032996	GC	1	48043687	A/C	0.928	-0.003	0.021	0.879	20.193
rs7489197	GC	12	38750065	T/C	0.286	-0.005	0.021	0.819	20.594
rs61842119	GC	10	17420907	C/T	0.186	-0.005	0.021	0.798	21.944
rs1229454	GC	7	81535972	A/G	0.470	-0.011	0.016	0.473	20.734
rs17161463	GC	5	108054728	T/C	0.220	-0.013	0.030	0.659	20.472
rs2157024	GC	16	13583664	G/A	0.320	-0.014	0.016	0.381	21.950
rs4927191	GC	1	55491702	C/T	0.246	-0.014	0.022	0.511	31.641
rs1587493	GC	2	234689751	A/G	0.859	-0.023	0.025	0.362	19.707
rs12594129	GC	15	89509679	C/A	0.029	-0.026	0.241	0.915	35.900
rs6835978	GC	4	47500814	G/A	0.200	-0.035	0.045	0.439	20.695
rs170704	GC	6	134253421	A/C	0.032	-0.167	0.132	0.207	20.887
rs142384226	GC	1	236784037	T/C	0.025	-0.341	0.257	0.184	20.337

SNP, Single Nucleotide SNP, Polymorphism; CHR, Chromosome; POS, Position; EA, Effect Allele; OA, Other Allele; EAF, Effect Allele Frequency; *β*, beta value; *SE*, Standard Error of beta; *P*, P-value*. SNPs were sorted in descending order by beta value (Allele Effect Value), all with *P* < 5×10^-8^.

**Table 3:** Results of MR analysis of the causal effect of MI on GC.

Outcome	Nsnp	MR			Heterogeneity			Horizontal pleiotropy			MR-PRESSO
			OR(95%CI)	P	I^2^(%)	Cochran's Q	P	Egger intercept	SE	P	P
GC	21	Inverse variance weighted	1.112 (1.002-1.233)	0.045	0	11.199	0.941				0.929
GC	21	MR Egger	1.062 (0.806-1.399)	0.675	0	11.073	0.921	0.005	0.015	0.727	
GC	21	Weighted median	1.049 (0.904-1.217)	0.528							
GC	21	Simple mode	1.144 (0.914-1.432)	0.253							
GC	21	Weighted mode	1.059 (0.925-1.212)	0.415							

### 3.2 Analysis of DEGs and their enrichment in MI and GC

**Figure 3:**
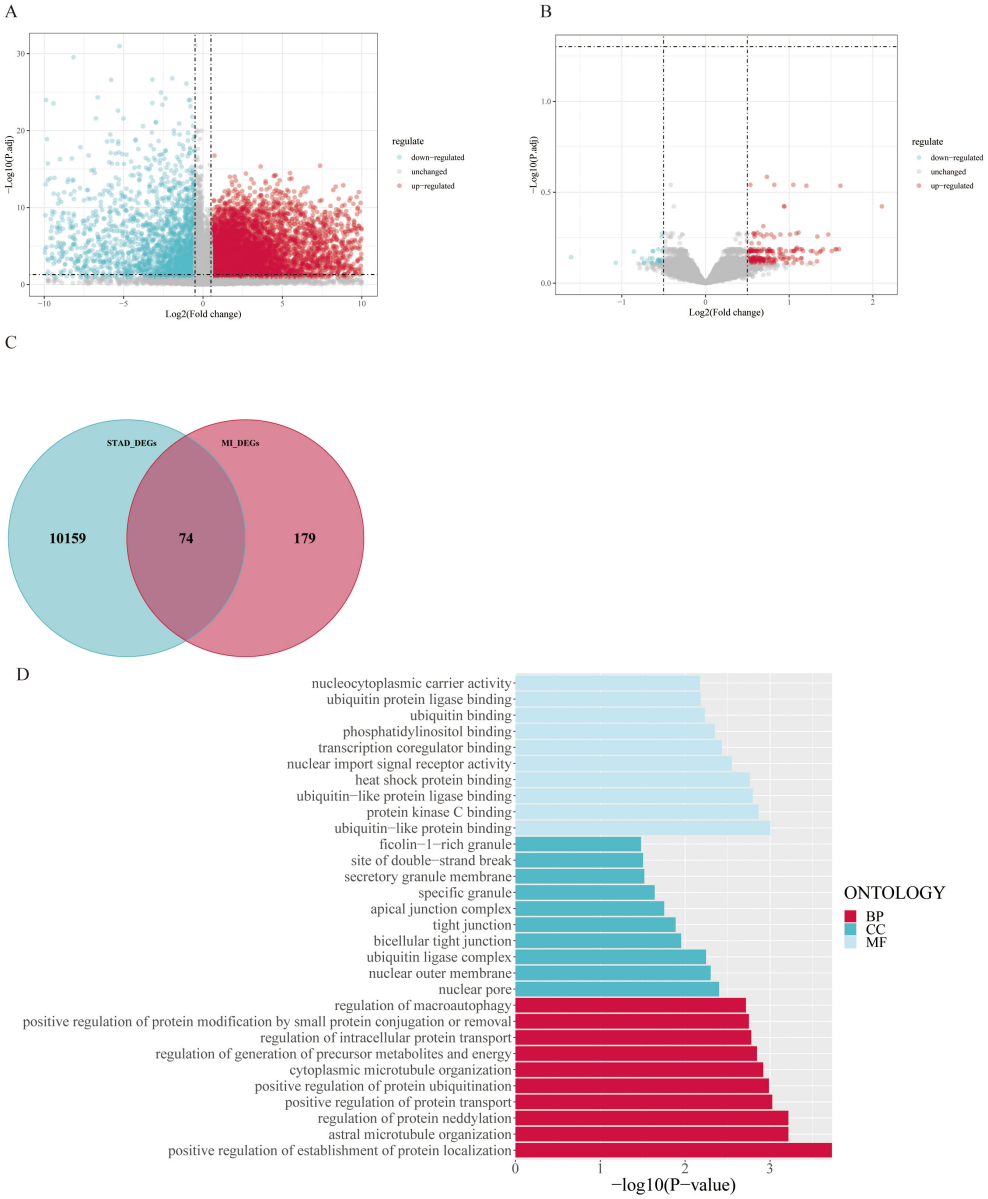
Differentially expressed gene analysis and enrichment analysis results: **(A)** Volcano plot of differentially expressed genes in the TCGA-STAD dataset, identifying 10,233 differentially expressed genes (*|log2(FC*)| > 0.5, *P-value* < 0.05). **(B)** Volcano plot of differentially expressed genes in the GSE83500 dataset, identifying 253 differentially expressed genes. **(C)** Venn diagram of the two datasets, showing 74 genes that are consistently differentially expressed in both datasets. **(D)** GO enrichment analysis of the intersecting genes, significantly enriched in pathways such as positive regulation of establishment of protein localization.

Differential expression analysis was performed on the transcriptome expression data of the TCGA-STAD dataset via the limma package, and 10,233 DEGs were identified by setting thresholds of |*log 2 FC*| >0.5 and *P* < 0.05 (**Figure 3A**). Similarly, differential expression analysis was performed on the GSE83500 dataset via the limma package, and 253 DEGs were screened with the same threshold (**Figure 3B**). We took the intersection of genes that were simultaneously present in both datasets and expressed in the same direction and obtained a total of 74 intersecting genes (**Figure 3C**).

We subsequently performed gene enrichment analysis, which revealed that the differentially expressed genes were significantly enriched in the positive regulation of the establishment of protein localization and other related pathways. Other biological processes that were significantly enriched included positive regulation of protein transport, positive regulation of protein ubiquitination, and stellate microtubule organization. In terms of cellular composition, the enriched terms were nuclear pores, the outer nuclear membrane, and the apical junction complex. Among the molecular functional categories, ubiquitin-protein ligase binding, heat shock protein binding, and transcriptional coregulator binding were notable (**Figure 3D**).

### 3.3 Expression of MI and GC common differential genes for quantitative trait loci validation of causal associations between eQTLs and GCs

**Figure 4:**
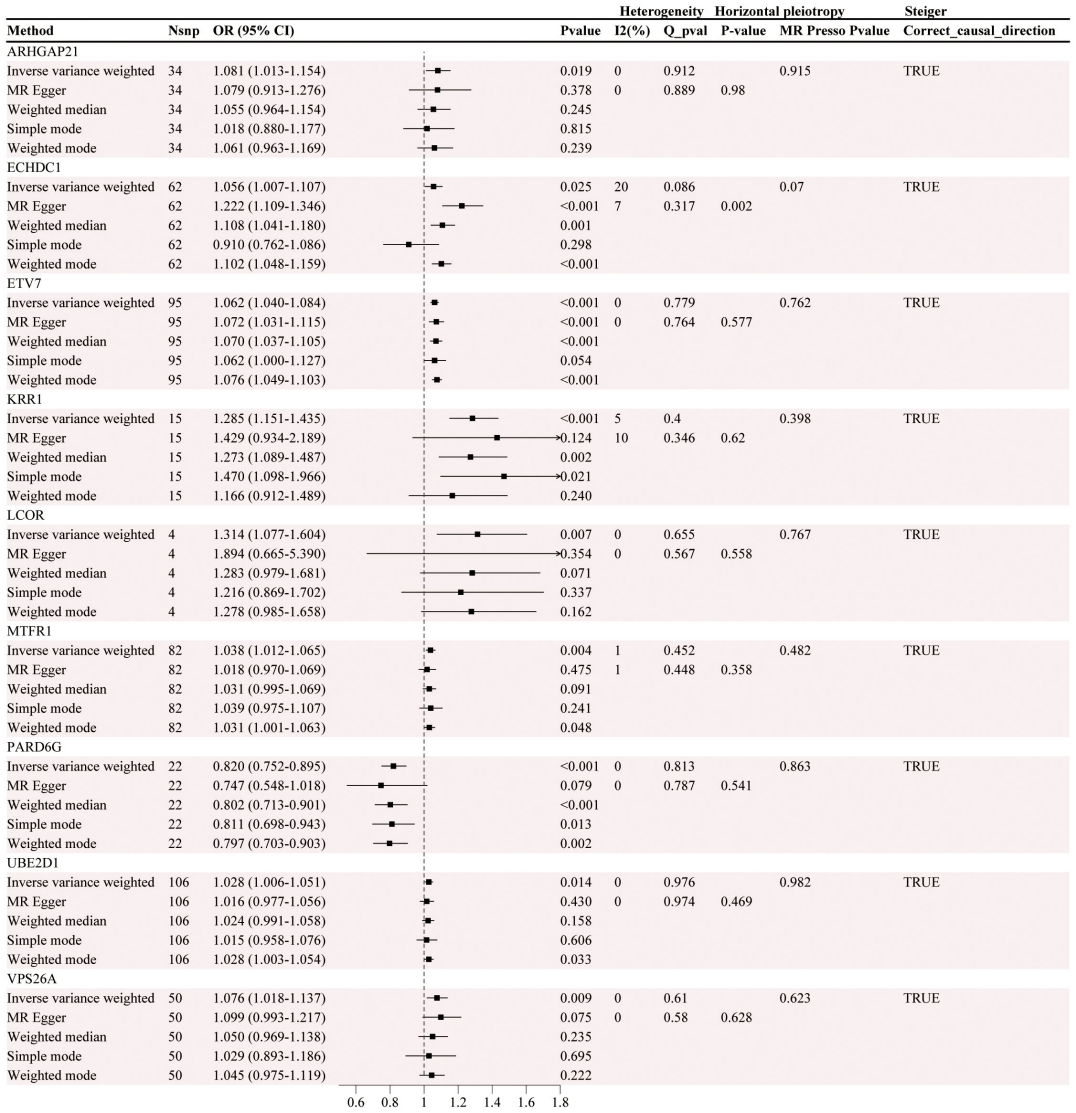
Causal association analysis results between gene eQTLs and gastric cancer.

After screening, 74 DEGs were identified, with only 37 DEGs from the blood eQTLs screened by available instrumental variables. The eQTLs for 69 differential genes were extracted from the IEU OpenGWAS program, and a total of 2101 cis eQTLs for differential genes were identified (**Supplementary Table 1**). Drug target MR analysis revealed that 9 genes were causally associated with GC in the same direction as their expression in GC and MI. Among them, 8 genes, namely, *LCOR, VPS26A, KRR1, ARHGAP21, ECHDC1, UBE2D1, MTFR1*, and *ETV7*, were positively associated with GC, suggesting that they are associated with an increased risk of developing GC. Another gene, *PARD6G*, was negatively associated with GC, indicating an association with a decreased risk of GC (**Figure 4**). In addition, the sensitivity test did not detect significant horizontal pleiotropy or heterogeneity, indicating that the MR results were robust. Finally, Steiger's direction test revealed that in the GC ebi-a-GCST90018849 dataset, the directions of the DEGs were all “TRUE”, indicating that the causal relationship between the DEGs and the outcome was consistent with the expected direction (**Tables 4**, **5**).

**Table 4:** Results of MR analysis of the causal effect of eqtl on GC.

Exposure	Outcome	Nsnp	MR			Heterogeneity			Horizontal pleiotropy			MR-PRESSO
			OR (95%CI)	*P*	FDR	I^2^ (%)	Cochran's Q	P	Egger intercept	SE	*P*	*P*
*LCOR*	GC	4	1.314 (1.077-1.604)	0.007	0.038	0	1.619	0.655	-0.035	0.051	0.558	0.767
*PARD6G*	GC	22	0.820 (0.752-0.895)	<0.001	<0.001	0	15.195	0.813	0.011	0.018	0.541	0.863
*VPS26A*	GC	50	1.076 (1.018-1.137)	0.009	0.045	0	45.638	0.610	-0.004	0.008	0.628	0.623
*KRR1*	GC	15	1.285 (1.151-1.435)	<0.001	<0.001	5	14.689	0.400	-0.012	0.024	0.620	0.398
*ARHGAP21*	GC	34	1.081 (1.013-1.154)	0.019	0.074	0	22.652	0.912	0.000	0.010	0.980	0.915
*ECHDC1*	GC	62	1.056 (1.007-1.107)	0.025	0.090	20	76.596	0.086	-0.040	0.012	0.002	0.07
*UBE2D1*	GC	106	1.028 (1.006-1.051)	0.014	0.058	0	78.337	0.976	0.005	0.006	0.469	0.982
*MTFR1*	GC	82	1.038 (1.012-1.065)	0.004	0.028	1	81.856	0.452	0.007	0.008	0.358	0.482
*ETV7*	GC	95	1.062 (1.040-1.084)	<0.001	<0.001	0	83.235	0.779	-0.003	0.006	0.577	0.762

**Table 5:** Steiger directionality test.

Exposure	Outcome	Correct_causal_direction	Steiger_pval
*ETV7*	GC	TRUE	0
*MTFR1*	GC	TRUE	0
*UBE2D1*	GC	TRUE	–
*ECHDC1*	GC	TRUE	0
*ARHGAP21*	GC	TRUE	0
*KRR1*	GC	TRUE	3.18E-259
*VPS26A*	GC	TRUE	0
*PARD6G*	GC	TRUE	5.07E-212
*LCOR*	GC	TRUE	2.22112E-18

### 3.4 Establishment and validation of gastric cancer-related prognostic models

**Figure 5:**
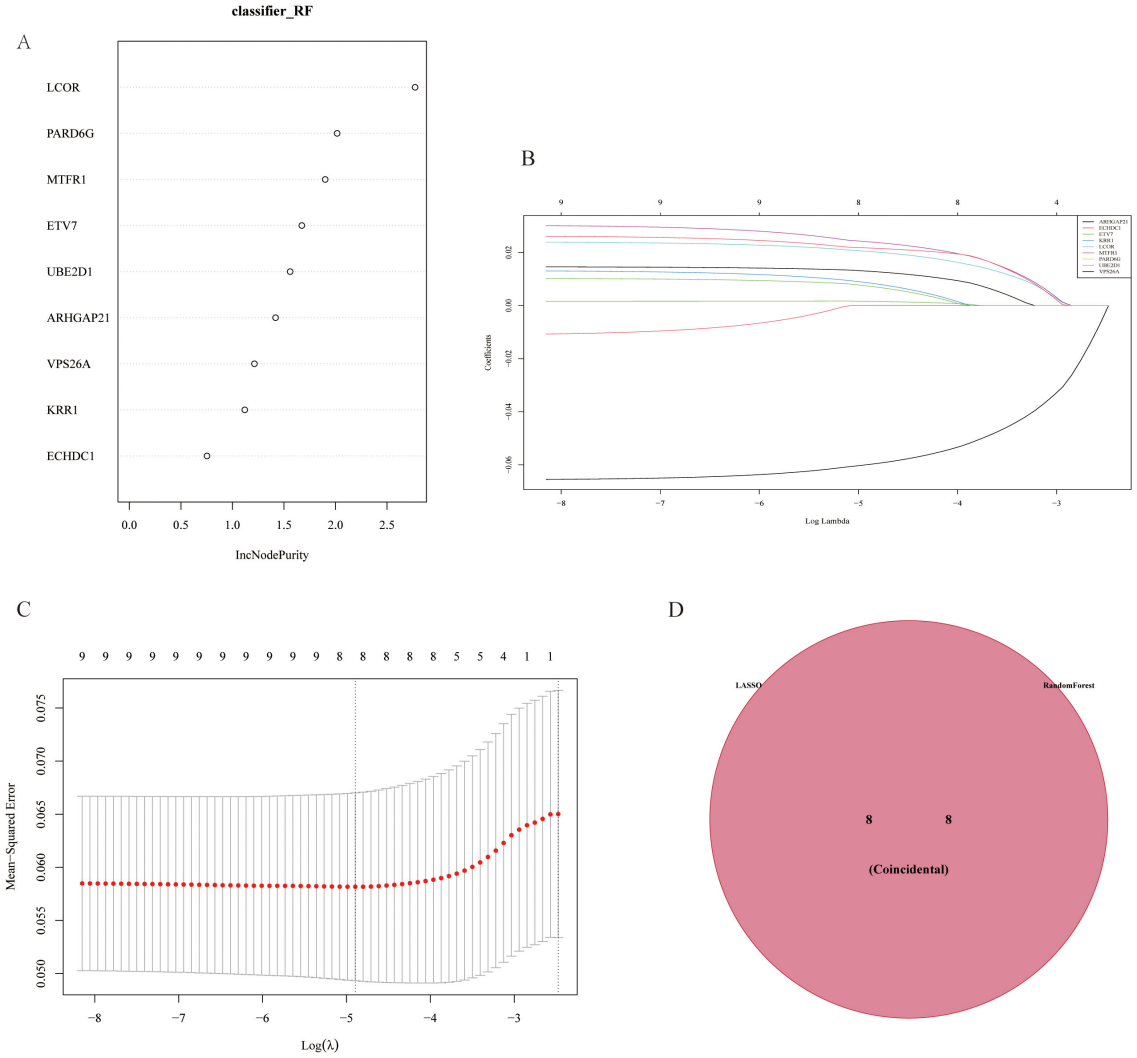
Gene selection results from LASSO and random forest model analyses. **(A)** Gene importance plot in the Random Forest (RF) model, showing genes with a high increase in node purity (incNodePurity). **(B)** Cross-validation for parameter tuning in LASSO analysis. **(C)** Least Absolute Shrinkage and Selection Operator (LASSO) regression model, which avoids overfitting by reducing redundant features and narrows down key differentially expressed genes associated with myocardial infarction. **(D)** Venn diagram of genes selected by both LASSO and RF models, showing eight genes identified by both methods, indicating their diagnostic value in predicting gastric cancer.

Based on the results of differential analysis and MR analysis, we screened a total of 9 genes with consistent direction and positive analysis results and used LASSO and RF to construct a GC prediction model to identify genes with diagnostic values for GC (**Figures 5A–C**). The results revealed that the LASSO model identified 8 genes with diagnostic value, and the RF model also identified 8 genes with diagnostic value. The intersection of the two methods revealed 8 common genes (**Figure 5D**).

**Figure 6:**
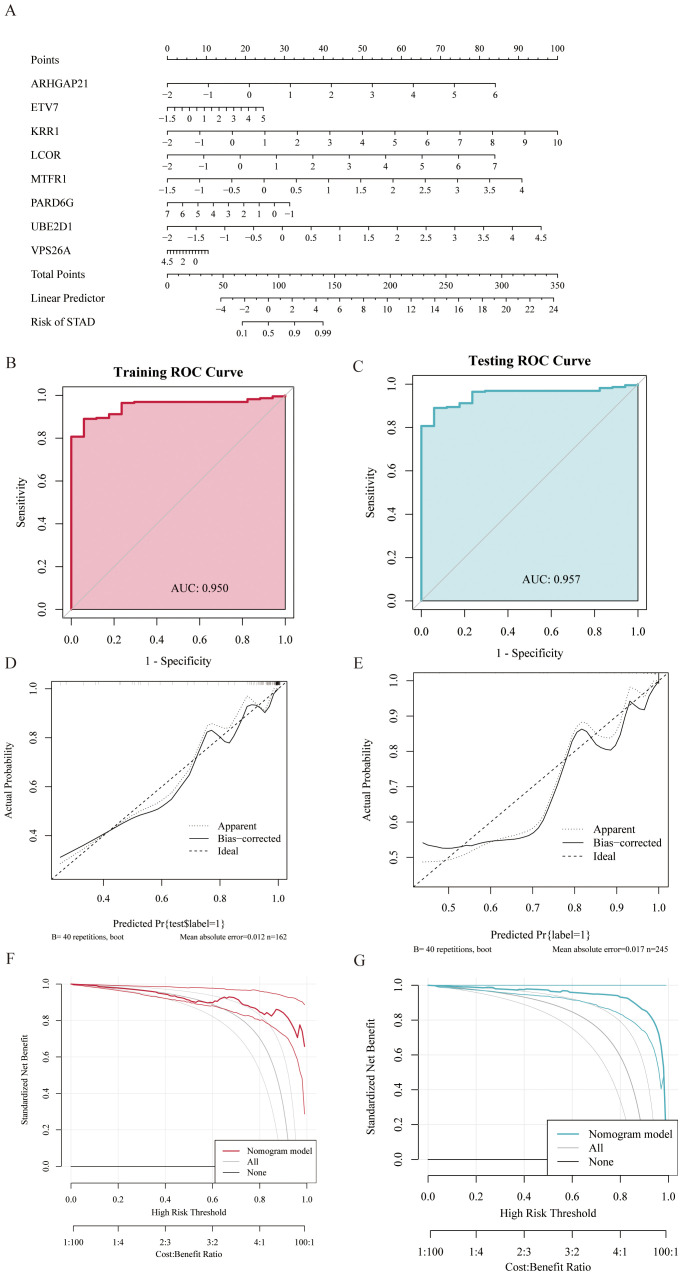
Gastric cancer prediction model constructed based on random forest (RF) and LASSO regression models. **(A)** Nomogram displaying eight diagnostic genes for gastric cancer and their scoring criteria to predict individual gastric cancer risk. **(B, C)** Receiver Operating Characteristic (ROC) curves showing Area Under the Curve (AUC) values above 0.95 in both training and testing sets, indicating the high discriminative ability of the model in gastric cancer diagnosis. **(D, E)** Calibration curves evaluate the consistency between model predictions and actual outcomes, demonstrating good calibration performance of the predictive model. **(F, G)** ROC curves showing AUC values above 0.95 in both training and testing sets, further indicating the model's strong diagnostic capability for gastric cancer. Decision Curve Analysis (DCA) shows that using the nomogram model provides a higher net benefit for patients.

To improve the diagnostic and predictive performance of GC patients, we constructed a nomogram based on 8 genes with diagnostic values (*ARHGAP21, ETV7, KRR1, LCOR, MTFR1, PARD6G, UBE2D1*, and *VPS26A*) via logistic regression analysis (**Figure 6A**). The AUC values of the model were all greater than 0.95, suggesting that the nomogram may have a strong diagnostic value for predicting GC (**Figures 6B, C**). The calibration curve revealed that the predicted probability of the nomogram model was almost identical to that of the ideal model (**Figures 6D, E**). Additionally, we performed DCA of the nomogram, which indicated that decisions based on the nomogram model may be beneficial for diagnosing GC (**Figures 6F, G**).

### 3.5 Correlation analysis of genes with diagnostic value and immune cell infiltration

**Figure 7:**
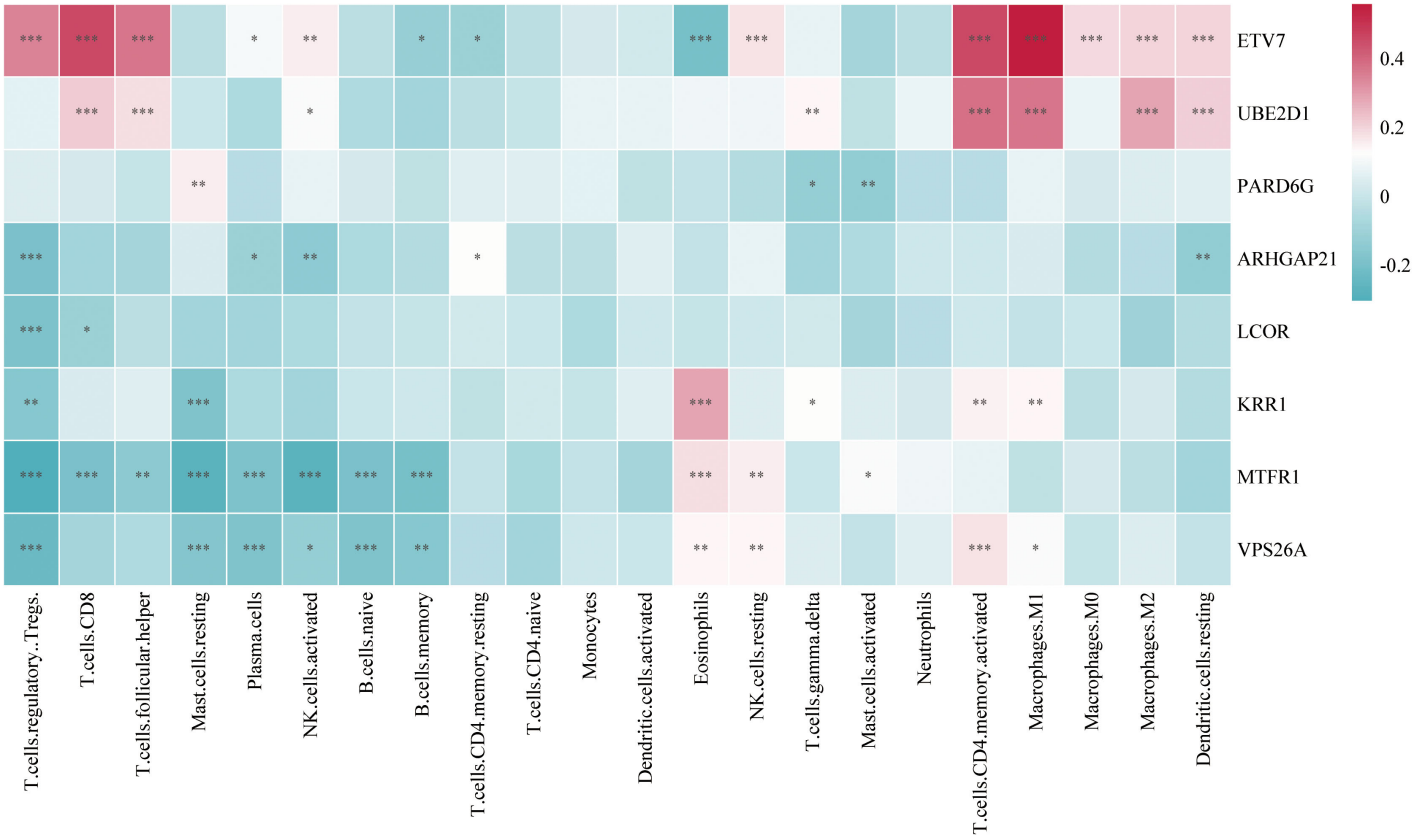
Heatmap of the correlation between key gene expression and infiltration of various immune cells in gastric cancer tissues (Color indicates the strength and direction of correlation; statistical significance levels: ns, *p* ≥ 0.05; *p < 0.05; **p < 0.01; ***p < 0.001.

In this study, we analyzed immune cell infiltration in GC, and the expression of the key gene *ETV7* was significantly and positively correlated with the degree of infiltration of T.cells.follicular.helper (*p*<0.001). The key gene *UBE2D1* was significantly positively correlated (*p*<0.001) with the degree of infiltration of Macrophage.M0, whereas *ETV7* was significantly negatively correlated (*p*<0.001) with Macrophage.M0. The expression of the key gene *ARHGAP21* was significantly positively correlated (*p*<0.001) with the degree of infiltration of NK.cells.resting, whereas *VPS26A* was significantly negatively correlated (*p*<0.01) with NK.cells.resting. The key gene *ARHGAP21* was significantly negatively correlated with the degree of infiltration of neutrophils (*p*<0.05), whereas *ETV7* was significantly positively correlated with the number of neutrophils (*p*<0.001). The expression of the key gene *PARD6G* was significantly negatively correlated (*p*<0.05) with the degree of infiltration of Dendritic.cells.resting (**Figure 7**).

## 4 Discussion

In this study, we systematically explored the common pathogenic factors of GC and MI and identified potential common drug targets by combining GEO data and eQTL data with MR analysis and transcriptomics analysis. First, through MR analysis of GWAS data of GC and MI, eQTLs of MI were found to be significantly associated with the risk of GC. In addition, 74 genes that were differentially expressed in GC and MI were screened via differential expression gene analysis. Nine genes were identified as potential drug targets for GC treatment via drug-targeted MR, and the directional consistency of the causal associations between the DEGs and GC was verified. A nomogram for GC was subsequently constructed based on 8 key genes, including *LCOR*, which were screened via random forest and LASSO regression algorithms, and it showed good predictive performance. In addition, the expression of key genes was significantly correlated with the degree of T-cell infiltration according to immune cell infiltration analysis, further suggesting the potential role of these genes in the immune microenvironment. In this study, by integrating multisource data and applying multiple methods, we revealed the common pathogenic mechanism of GC and MI, identified multiple potential drug targets, and provided new ideas for future disease prevention and drug intervention. By combining the advantages of MR analysis, transcriptomics, and machine learning modeling, this paper provides more comprehensive insights into the comorbidities of GC and MI, with important clinical applications.

Existing studies have found that patients with myocardial infarction (MI) have a significantly higher risk of developing cancer both in the short and long term, especially within the first 6 months after MI (20). A study by Maarten J G Leening and colleagues showed that, although the overall cancer incidence among STEMI patients over 5 years was similar to that of the general population, the cancer risk was significantly higher in the first 3 months after STEMI (12). Although gastric cancer (GC) and MI affect different systems, there may be a potential link between the two. Logan Vincent and colleagues reported that GC and MI share several pathogenic factors, such as inflammation, smoking, and high-salt diets, which may contribute to the development and progression of both diseases (46). Ryan J Koene's article emphasized that inflammation is a key mechanism in both cardiovascular disease (CVD) and cancer. Common risk factors like obesity, hyperglycemia, hypertension, and hypertriglyceridemia can trigger inflammation, leading to shared risk for both diseases. Additionally, hormones, cytokines, and growth factors might also play a role in the biological connection between these diseases (47). Therefore, exploring the causal relationship between GC and MI is important for understanding this association. However, most of the previous studies used observational study designs, which revealed the overlap between the two in terms of causative factors but were limited by confounding factors and reverse causality, making it difficult to draw clear causal inferences. To address this problem, MR, as an emerging analytical tool, provides a more reliable basis for causal inference by using genetic variation as an instrumental variable to simulate a randomized controlled trial, which reduces the interference of environmental and behavioral differences on the results (48). In this study, for the first time, we not only verified the potential causal association between GC and MI via MR analysis but also revealed the common differential genes and potential drug targets by combining it with transcriptomics analysis. Unlike previous studies that explored only shared risk factors, the present study explored the link between GC and MI at the genetic level in detail, identifying multiple molecular mechanisms that are jointly involved. In addition, while previous studies have not systematically analyzed the common targets of GC and MI, this study fills this gap by identifying multiple possible therapeutic targets through drug target MR analysis. Moreover, this study screened key genes with diagnostic value via machine learning algorithms (e.g., random forest and LASSO regression) and constructed a column-line graph model with high predictive performance, which provides new ideas for the early diagnosis of GC and MI in the future. Therefore, this study is not only a revalidation of previous studies but also an innovation in the exploration of pathogenic mechanisms and clinical applications, which fills the research gaps in related fields, promotes an in-depth understanding of the common pathogenesis of GC and MI, and provides potential drug targets and diagnostic tools for future therapeutic strategies.

In this study, the 74 DEGs identified by GO enrichment analysis were significantly enriched in several key biological processes, cellular compositions and molecular functions. Specifically, at the biological process (BP) level, these genes were significantly enriched in the positive regulation of establishment of protein localization, protein transport, and protein ubiquitination modification, and ubiquitin-dependent protein catabolic process. These biological processes are critical in cancer and cardiovascular diseases, and the correct localization and modification of proteins play decisive roles in the maintenance of normal cell function, signaling, and the regulation of cell proliferation and metabolism. Abnormal localization of proteins may lead to cellular dysfunction and thus drive cancer progression (49), whereas in MI, abnormal protein transport affects cardiomyocyte function (50). At the cellular component (CC) level, these genes were enriched predominantly in the nuclear pore complex, endoplasmic reticulum membrane and Golgi apparatus. The abnormal function of these cellular structures is closely related to the development of GC and MI. Studies have shown that altered Golgi function in GC affects protein transport, leading to tumor cell proliferation and metastasis (51). In MI, disruption of the nuclear pore complex may affect the exchange of materials in the nucleus, thereby aggravating cellular damage. At the molecular function (MF) level, these genes were enriched for ubiquitin protein ligase binding and heat shock protein binding, which play key roles in protein modification, protein folding and the stress response. In particular, the ubiquitination pathway plays a central role in cell cycle regulation, DNA repair, and signaling. Abnormal ubiquitination is an important mechanism in the development of GC (52) and MI (53).

By integrating differentially expressed genes with their corresponding eQTL data, we further carried out MR analysis of drug targets to explore the causal associations between these genes and the risk of GC. This analysis not only verified the potential causal relationship between genes and diseases but also provided an important theoretical basis for the development of future drug targets. According to the results of MR analysis, the eQTLs of nine genes showed significant causal associations with the risk of GC development, suggesting that they may be common potential therapeutic targets for both. Among these genes, those associated with an increased risk of GC development included *LCOR, VPS26A, KRR1, ARHGAP21, ECHDC1, UBE2D1, MTFR1*, and *ETV7*, whereas the gene associated with a decreased risk of GC development was *PARD6G*. Previous studies have shown that high expression of *LCOR* in GC is independently associated with poor prognosis, suggesting that it may be a common potential therapeutic target. These findings suggest that *LCOR* may play a key role in GC progression (54). *VPS26A* is a protein involved in intracellular retrograde transport and is responsible for transporting proteins from endosomes to the Golgi apparatus as part of the reticulum complex. Although *VPS26A* has been shown to regulate cell proliferation, migration, and invasive ability in a variety of tumors, thereby promoting tumor progression, its specific function in GC needs to be further investigated (55). *KRR1* is mainly responsible for the formation of the 40S ribosomal subunit and is thought to correlate with drug response and tumor progression, especially in the use of S-1, cisplatin, and docetaxel. *ARHGAP21* acts as a Rho GTPase-activating protein and converts Rho family GTPases from active to inactive states, mainly by regulating cytoskeletal activities (56). In prostate cancer, *ARHGAP21* affects tumor progression by regulating the expression of the *PCA3* gene, but its specific role in GC is still unclear (57). *ECHDC1*, as a metabolism-correcting enzyme, is involved in fatty acid synthesis, which is associated with drug resistance in tumors such as bladder cancer, but the specific mechanism of *ECHDC* in GC still needs to be further studied (58). *UBE2D1* is a ubiquitin-binding enzyme, and its high expression has been shown to promote epithelial−mesenchymal transition (EMT) through the *TGF-β/SMAD4* signaling pathway to increase the migration and invasion of GC cells (59). *MTFR1*, a protein that regulates mitochondrial fission, has been demonstrated to be correlated with poor prognosis and drug resistance in *NSCLC* (60) and drug resistance in non-small cell lung cancer, but studies in GC have not yet reached a definitive conclusion (61). *ETV7* is a member of the Ets family of transcription factors and plays a regulatory role in cell differentiation and proliferation. Studies have shown that *ETV7* expression is correlated with decreased sensitivity of GC cells to chemotherapeutic agents such as 5-FU and *CDDP* [PMID: 24504010]. *PARD6G*, a parapolar protein, is involved in the regulation of cell polarity, and studies have demonstrated that its differential expression among subtypes of lung cancer has a role as a potential marker; however, its function in GC has not yet been explored in depth [PMID: 32360590]. The robust causal relationship between these genes and their consistent expression in GC and MI suggests that they may play important roles in the common pathological mechanisms of these two diseases, providing strong support for drug target development and clinical intervention strategies.

To verify the potential application value of differential genes in the diagnosis of GC, this study used random forest and LASSO regression algorithms to construct a prediction model for GC. The results revealed that the LASSO model screened eight genes with diagnostic value, and the random forest model also screened eight genes with diagnostic value. The intersecting genes of the two were ultimately used in the construction of the prediction model. The nomogram model constructed on the basis of these genes had high prediction performance, and its AUC values were all greater than 0.95, suggesting that the model has high accuracy in the diagnosis of GC. Moreover, calibration curve and decision curve analysis (DCA) further indicated that the predictive model had good calibration and decision value. These results suggest that the predictive model based on differential genes not only has potential application in the diagnosis of GC but also may provide a reference for the diagnosis and prediction of MI.

We subsequently constructed a prediction model for GC via LASSO regression and a random forest model and ultimately identified 8 genes with diagnostic value. The column-line graph model based on these eight genes demonstrated extremely high diagnostic efficacy, with AUC values greater than 0.95, suggesting that these genes have important potential for clinical application in the diagnosis and prediction of GC. In addition, immune cell infiltration analysis revealed that some of the key genes were positively correlated with the infiltration of T cells (especially CD8+ T cells), further supporting the important role of these genes in the immune response.

This study, which explored the common pathogenic mechanisms and potential drug targets of GC and MI, had several limitations despite the application of techniques such as MR, transcriptome analysis and machine learning. First, the data sources are based mainly on European populations, and although these data provide rich genotypic and phenotypic information, the incidence and pathogenic mechanisms of GC and MI differ in different populations. Therefore, the global applicability of the findings, especially their validity in Asian populations, needs to be verified. Second, although we identified 74 differential genes in the transcriptome analysis, only 37 instrumental variables of blood eQTLs were ultimately screened for use in MR analysis, which limited the causal analysis of more potential key genes. In addition, the cross-sectional data used in this study failed to reflect the dynamics of the disease at different stages and could not reveal changes in gene action over time. Future studies should incorporate longitudinal data combined with time-dimensional analysis to further elucidate the dynamic pathogenic mechanisms of GC and MI. Finally, although we screened potential targets through our analysis, the clinical feasibility of these targets still needs to be verified through cellular experiments, animal models, and clinical trials to determine their effectiveness and safety in therapy. Therefore, future studies should further validate the biological functions of these key genes by experimental means to ensure their feasibility and effectiveness as clinical targets. Finally, although we constructed predictive models for GC by machine learning and the diagnostic performance of the models showed high AUC values, these models still need to be validated by independent external datasets to ensure their robustness and generalizability in different populations and practical clinical applications.

## 5 Conclusion

The MR analysis in this study indicated that MI may increase the risk of GC. Eight potential diagnostic genes associated with MI and GC were identified through transcriptome analysis and drug target MR validation, and a nomogram model with good predictive performance was successfully constructed. The results provide new insights into the comorbid mechanisms of MI and GC and provide a theoretical basis for the optimization of individualized diagnosis and prognosis assessment strategies.

## Data Availability

The original contributions presented in the study are included in the article/**Supplementary Material**. Further inquiries can be directed to the corresponding author.
