# Supplementary material for: Analysis of shared pathogenic mechanisms and drug targets in myocardial infarction and gastric cancer based on transcriptomics and machine learning
Source: Front Immunol. 2025 Mar 21;16:1533959. doi: 10.3389/fimmu.2025.1533959 (PMC11968731; doi:10.3389/fimmu.2025.1533959)
Supplement: Supplementary file 2 [file Table1.docx]

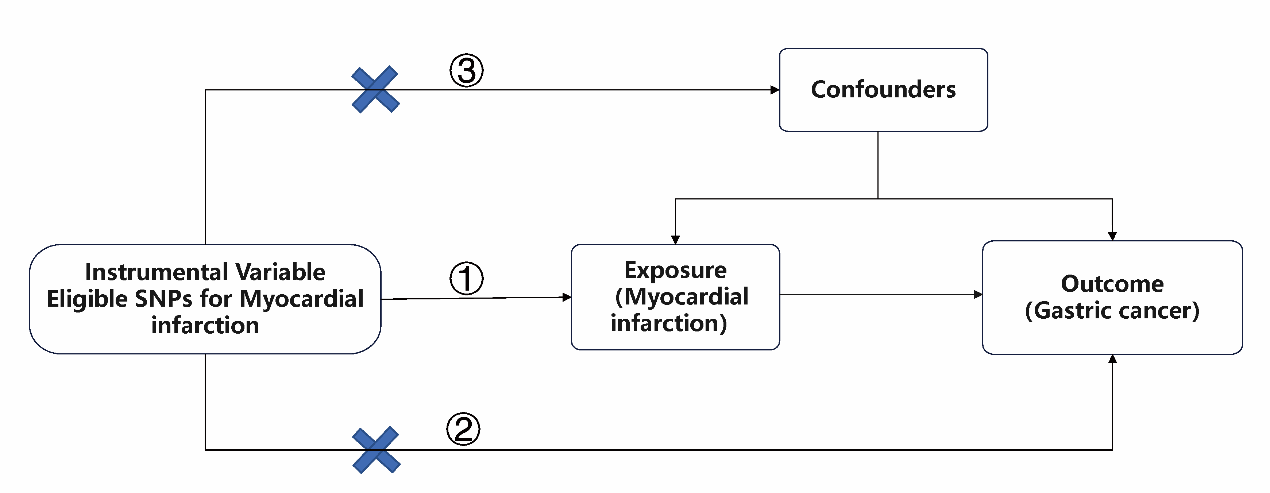


Figure S1 Schematic of Mendelian randomization of the association between Myocardial infarction and gastric cancer

Table S1 Detailed Information of eQTL and GC Datasets

| **Data source** | **Phenotype** | **Sample size** | **Cases** | **Population** | **Adjustment** |
| --- | --- | --- | --- | --- | --- |
| IEU Open GWAS project(eqtl-a-ENSG00000047188) | YTHDC2 | 31684 | - | European | Males and Females |
| IEU Open GWAS project(eqtl-a-ENSG00000066855) | MTFR1 | 31684 | - | European | Males and Females |
| IEU Open GWAS project(eqtl-a-ENSG00000067066) | SP100 | 31684 | - | European | Males and Females |
| IEU Open GWAS project(eqtl-a-ENSG00000077097) | TOP2B | 31684 | - | European | Males and Females |
| IEU Open GWAS project(eqtl-a-ENSG00000100644) | HIF1A | 31684 | - | European | Males and Females |
| IEU Open GWAS project(eqtl-a-ENSG00000108848) | LUC7L3 | 31684 | - | European | Males and Females |
| IEU Open GWAS project(eqtl-a-ENSG00000111615) | KRR1 | 31684 | - | European | Males and Females |
| IEU Open GWAS project(eqtl-a-ENSG00000115145) | STAM2 | 31684 | - | European | Males and Females |
| IEU Open GWAS project(eqtl-a-ENSG00000115355) | CCDC88A | 31684 | - | European | Males and Females |
| IEU Open GWAS project(eqtl-a-ENSG00000115762) | PLEKHB2 | 31684 | - | European | Males and Females |
| IEU Open GWAS project(eqtl-a-ENSG00000117174) | ZNHIT6 | 31684 | - | European | Males and Females |
| IEU Open GWAS project(eqtl-a-ENSG00000119335) | SET | 31684 | - | European | Males and Females |
| IEU Open GWAS project(eqtl-a-ENSG00000135486) | HNRNPA1 | 31684 | - | European | Males and Females |
| IEU Open GWAS project(eqtl-a-ENSG00000137221) | TJAP1 | 31684 | - | European | Males and Females |
| IEU Open GWAS project(eqtl-a-ENSG00000137831) | UACA | 31684 | - | European | Males and Females |
| IEU Open GWAS project(eqtl-a-ENSG00000143183) | TMCO1 | 31684 | - | European | Males and Females |
| IEU Open GWAS project(eqtl-a-ENSG00000152413) | HOMER1 | 31684 | - | European | Males and Females |
| IEU Open GWAS project(eqtl-a-ENSG00000162231) | NXF1 | 31684 | - | European | Males and Females |
| IEU Open GWAS project(eqtl-a-ENSG00000163029) | SMC6 | 31684 | - | European | Males and Females |
| IEU Open GWAS project(eqtl-a-ENSG00000164022) | AIMP1 | 31684 | - | European | Males and Females |
| IEU Open GWAS project(eqtl-a-ENSG00000170581) | STAT2 | 31684 | - | European | Males and Females |
| IEU Open GWAS project(eqtl-a-ENSG00000177889) | UBE2N | 31684 | - | European | Males and Females |
| IEU Open GWAS project(eqtl-a-ENSG00000178184) | PARD6G | 31684 | - | European | Males and Females |
| IEU Open GWAS project(eqtl-a-ENSG00000178691) | SUZ12 | 31684 | - | European | Males and Females |
| IEU Open GWAS project(eqtl-a-ENSG00000189043) | NDUFA4 | 31684 | - | European | Males and Females |
| IEU Open GWAS project(eqtl-a-ENSG00000189180) | ZNF33A | 31684 | - | European | Males and Females |
| IEU Open GWAS project(eqtl-a-ENSG00000085274) | MYNN | 31644 | - | European | Males and Females |
| IEU Open GWAS project(eqtl-a-ENSG00000127334) | DYRK2 | 31644 | - | European | Males and Females |
| IEU Open GWAS project(eqtl-a-ENSG00000196233) | LCOR | 31644 | - | European | Males and Females |
| IEU Open GWAS project(eqtl-a-ENSG00000054654) | SYNE2 | 31470 | - | European | Males and Females |
| IEU Open GWAS project(eqtl-a-ENSG00000090989) | EXOC1 | 31470 | - | European | Males and Females |
| IEU Open GWAS project(eqtl-a-ENSG00000109445) | ZNF330 | 31470 | - | European | Males and Females |
| IEU Open GWAS project(eqtl-a-ENSG00000112697) | TMEM30A | 31470 | - | European | Males and Females |
| IEU Open GWAS project(eqtl-a-ENSG00000114062) | UBE3A | 31470 | - | European | Males and Females |
| IEU Open GWAS project(eqtl-a-ENSG00000122958) | VPS26A | 31470 | - | European | Males and Females |
| IEU Open GWAS project(eqtl-a-ENSG00000123352) | SPATS2 | 31470 | - | European | Males and Females |
| IEU Open GWAS project(eqtl-a-ENSG00000143457) | GOLPH3L | 31470 | - | European | Males and Females |
| IEU Open GWAS project(eqtl-a-ENSG00000145685) | LHFPL2 | 31470 | - | European | Males and Females |
| IEU Open GWAS project(eqtl-a-ENSG00000163453) | IGFBP7 | 31470 | - | European | Males and Females |
| IEU Open GWAS project(eqtl-a-ENSG00000163848) | ZNF148 | 31470 | - | European | Males and Females |
| IEU Open GWAS project(eqtl-a-ENSG00000170266) | GLB1 | 31470 | - | European | Males and Females |
| IEU Open GWAS project(eqtl-a-ENSG00000170540) | ARL6IP1 | 31470 | - | European | Males and Females |
| IEU Open GWAS project(eqtl-a-ENSG00000010030) | ETV7 | 31430 | - | European | Males and Females |
| IEU Open GWAS project(eqtl-a-ENSG00000106460) | TMEM106B | 31430 | - | European | Males and Females |
| IEU Open GWAS project(eqtl-a-ENSG00000184007) | PTP4A2 | 31256 | - | European | Males and Females |
| IEU Open GWAS project(eqtl-a-ENSG00000093144) | ECHDC1 | 31086 | - | European | Males and Females |
| IEU Open GWAS project(eqtl-a-ENSG00000140563) | MCTP2 | 30935 | - | European | Males and Females |
| IEU Open GWAS project(eqtl-a-ENSG00000151849) | CENPJ | 30935 | - | European | Males and Females |
| IEU Open GWAS project(eqtl-a-ENSG00000160255) | ITGB2 | 30721 | - | European | Males and Females |
| IEU Open GWAS project(eqtl-a-ENSG00000198830) | HMGN2 | 28877 | - | European | Males and Females |
| IEU Open GWAS project(eqtl-a-ENSG00000156735) | BAG4 | 28703 | - | European | Males and Females |
| IEU Open GWAS project(eqtl-a-ENSG00000205352) | PRR13 | 28703 | - | European | Males and Females |
| IEU Open GWAS project(eqtl-a-ENSG00000048162) | NOP16 | 26609 | - | European | Males and Females |
| IEU Open GWAS project(eqtl-a-ENSG00000148468) | FAM171A1 | 26609 | - | European | Males and Females |
| IEU Open GWAS project(eqtl-a-ENSG00000163961) | RNF168 | 26609 | - | European | Males and Females |
| IEU Open GWAS project(eqtl-a-ENSG00000113356) | POLR3G | 26196 | - | European | Males and Females |
| IEU Open GWAS project(eqtl-a-ENSG00000118873) | RAB3GAP2 | 26196 | - | European | Males and Females |
| IEU Open GWAS project(eqtl-a-ENSG00000242485) | MRPL20 | 26011 | - | European | Males and Females |
| IEU Open GWAS project(eqtl-a-ENSG00000156136) | DCK | 25820 | - | European | Males and Females |
| IEU Open GWAS project(eqtl-a-ENSG00000043093) | DCUN1D1 | 14263 | - | European | Males and Females |
| IEU Open GWAS project(eqtl-a-ENSG00000072401) | UBE2D1 | 14263 | - | European | Males and Females |
| IEU Open GWAS project(eqtl-a-ENSG00000102898) | NUTF2 | 14263 | - | European | Males and Females |
| IEU Open GWAS project(eqtl-a-ENSG00000107863) | ARHGAP21 | 14263 | - | European | Males and Females |
| IEU Open GWAS project(eqtl-a-ENSG00000108424) | KPNB1 | 14263 | - | European | Males and Females |
| IEU Open GWAS project(eqtl-a-ENSG00000120675) | DNAJC15 | 14263 | - | European | Males and Females |
| IEU Open GWAS project(eqtl-a-ENSG00000164332) | UBLCP1 | 14263 | - | European | Males and Females |
| IEU Open GWAS project(eqtl-a-ENSG00000230124) | ACBD6 | 10577 | - | European | Males and Females |
| IEU Open GWAS project(eqtl-a-ENSG00000128059) | PPAT | 9188 | - | European | Males and Females |
| IEU Open GWAS project(eqtl-a-ENSG00000204577) | LILRB3 | 9188 | - | European | Males and Females |
| IEU Open GWAS project(ebi-a-GCST90018849) | Gastric cancer | 476116 | 1029 | European | - |

Table S2 SNP Information for eQTLs of Shared Differentially Expressed Genes in MI and GC

| **SNP** | **Outcome** | **CHR** | **POS** | **EA/OA** | **EAF** | **β*** | **SE** | **P** | **F-statistic** |
| --- | --- | --- | --- | --- | --- | --- | --- | --- | --- |
| ZNF330 |  |  |  |  |  |  |  |  |  |
| rs151211221 | Gastric cancer | 4 | 142135488 | C/T | 0.045 | 0.278 | 0.110 | 0.012 | 20.783 |
| rs138371866 | Gastric cancer | 4 | 142276494 | A/G | 0.026 | 0.164 | 0.145 | 0.258 | 22.165 |
| rs114307200 | Gastric cancer | 4 | 142056534 | A/G | 0.051 | 0.051 | 0.103 | 0.624 | 25.429 |
| rs10519586 | Gastric cancer | 4 | 142066636 | G/A | 0.225 | 0.035 | 0.054 | 0.514 | 202.636 |
| rs62327676 | Gastric cancer | 4 | 141992592 | T/C | 0.051 | 0.032 | 0.102 | 0.754 | 21.502 |
| rs754851 | Gastric cancer | 4 | 142333572 | T/C | 0.056 | 0.020 | 0.034 | 0.544 | 24.750 |
| rs146685536 | Gastric cancer | 4 | 142229746 | T/C | 0.058 | 0.020 | 0.034 | 0.554 | 44.477 |
| rs978157 | Gastric cancer | 4 | 142177058 | G/A | 0.473 | 0.018 | 0.016 | 0.264 | 62.647 |
| rs12510721 | Gastric cancer | 4 | 142278257 | C/T | 0.319 | 0.017 | 0.018 | 0.355 | 64.399 |
| rs72728297 | Gastric cancer | 4 | 142454364 | G/A | 0.086 | 0.016 | 0.081 | 0.841 | 30.067 |
| rs6841849 | Gastric cancer | 4 | 142164078 | G/A | 0.206 | 0.011 | 0.023 | 0.635 | 172.413 |
| rs72726212 | Gastric cancer | 4 | 142096381 | G/A | 0.031 | 0.008 | 0.130 | 0.954 | 85.126 |
| rs3910325 | Gastric cancer | 4 | 142078502 | C/A | 0.545 | 0.005 | 0.018 | 0.791 | 189.926 |
| rs13102572 | Gastric cancer | 4 | 142198497 | C/T | 0.419 | 0.001 | 0.017 | 0.975 | 40.137 |
| rs3733484 | Gastric cancer | 4 | 142155518 | T/G | 0.325 | 0.000 | 0.016 | 0.989 | 794.149 |
| rs1979948 | Gastric cancer | 4 | 142018592 | C/T | 0.893 | 0.000 | 0.022 | 0.988 | 25.990 |
| rs10001746 | Gastric cancer | 4 | 141948889 | C/T | 0.252 | -0.008 | 0.052 | 0.885 | 23.376 |
| rs4956285 | Gastric cancer | 4 | 142180045 | C/T | 0.380 | -0.013 | 0.016 | 0.415 | 204.284 |
| rs75554812 | Gastric cancer | 4 | 142138511 | C/T | 0.031 | -0.015 | 0.133 | 0.910 | 121.058 |
| rs62327565 | Gastric cancer | 4 | 142136585 | A/G | 0.060 | -0.026 | 0.097 | 0.789 | 182.323 |
| rs143003356 | Gastric cancer | 4 | 142016471 | A/G | 0.028 | -0.031 | 0.143 | 0.830 | 19.704 |
| rs116181457 | Gastric cancer | 4 | 142312050 | G/A | 0.026 | -0.033 | 0.145 | 0.823 | 19.811 |
| rs115304316 | Gastric cancer | 4 | 142185551 | A/G | 0.022 | -0.033 | 0.155 | 0.829 | 66.957 |
| rs79989952 | Gastric cancer | 4 | 142116338 | T/C | 0.043 | -0.038 | 0.110 | 0.730 | 21.534 |
| rs1897925 | Gastric cancer | 4 | 142150338 | A/G | 0.895 | -0.063 | 0.075 | 0.400 | 26.585 |
| rs72726287 | Gastric cancer | 4 | 142227557 | T/G | 0.072 | -0.080 | 0.087 | 0.359 | 77.440 |
| rs111832426 | Gastric cancer | 4 | 142043169 | A/G | 0.012 | -0.092 | 0.210 | 0.663 | 35.441 |
| rs72728281 | Gastric cancer | 4 | 142437171 | G/A | 0.026 | -0.101 | 0.142 | 0.477 | 53.032 |
| rs62325276 | Gastric cancer | 4 | 142284387 | T/C | 0.056 | -0.129 | 0.099 | 0.193 | 104.338 |
| rs1012864 | Gastric cancer | 4 | 142168903 | A/C | 0.039 | -0.161 | 0.118 | 0.174 | 50.415 |
| rs10004930 | Gastric cancer | 4 | 142192970 | A/G | 0.023 | -0.248 | 0.152 | 0.103 | 23.305 |
| rs41280519 | Gastric cancer | 4 | 142152327 | G/A | 0.014 | -0.326 | 0.199 | 0.101 | 23.276 |
| IGFBP7 |  |  |  |  |  |  |  |  |  |
| rs114081993 | Gastric cancer | 4 | 57985479 | T/C | 0.018 | 0.328 | 0.171 | 0.055 | 23.555 |
| rs75473212 | Gastric cancer | 4 | 57806515 | G/A | 0.030 | 0.221 | 0.131 | 0.092 | 34.749 |
| rs75310502 | Gastric cancer | 4 | 58003919 | T/C | 0.016 | 0.202 | 0.194 | 0.298 | 20.429 |
| rs113489532 | Gastric cancer | 4 | 58003240 | T/C | 0.014 | 0.122 | 0.197 | 0.535 | 25.240 |
| rs115510849 | Gastric cancer | 4 | 57692256 | A/G | 0.031 | 0.095 | 0.131 | 0.467 | 51.909 |
| rs11133469 | Gastric cancer | 4 | 57809045 | C/T | 0.226 | 0.088 | 0.065 | 0.179 | 365.710 |
| rs111927443 | Gastric cancer | 4 | 57819280 | T/C | 0.020 | 0.071 | 0.160 | 0.658 | 33.972 |
| rs188042204 | Gastric cancer | 4 | 57752547 | T/C | 0.024 | 0.055 | 0.145 | 0.707 | 23.206 |
| rs79743032 | Gastric cancer | 4 | 57759065 | T/C | 0.025 | 0.050 | 0.142 | 0.724 | 48.539 |
| rs5002004 | Gastric cancer | 4 | 57954457 | G/A | 0.870 | 0.042 | 0.030 | 0.158 | 91.258 |
| rs111757556 | Gastric cancer | 4 | 57681857 | T/C | 0.129 | 0.041 | 0.067 | 0.542 | 32.790 |
| rs116906920 | Gastric cancer | 4 | 57644286 | T/C | 0.104 | 0.039 | 0.075 | 0.605 | 36.201 |
| rs1718865 | Gastric cancer | 4 | 57886301 | A/G | 0.198 | 0.039 | 0.044 | 0.383 | 367.807 |
| rs75559205 | Gastric cancer | 4 | 57891482 | G/A | 0.025 | 0.036 | 0.143 | 0.799 | 37.304 |
| rs10024901 | Gastric cancer | 4 | 57961010 | A/G | 0.095 | 0.034 | 0.020 | 0.093 | 105.333 |
| rs35297343 | Gastric cancer | 4 | 57963121 | C/T | 0.064 | 0.031 | 0.095 | 0.745 | 38.213 |
| rs781561 | Gastric cancer | 4 | 57714659 | A/G | 0.522 | 0.028 | 0.016 | 0.084 | 124.248 |
| rs11725261 | Gastric cancer | 4 | 57914217 | T/C | 0.181 | 0.027 | 0.058 | 0.641 | 425.346 |
| rs704637 | Gastric cancer | 4 | 57713592 | G/T | 0.740 | 0.025 | 0.017 | 0.137 | 23.004 |
| rs1277313 | Gastric cancer | 4 | 57903948 | G/T | 0.706 | 0.021 | 0.016 | 0.187 | 29.289 |
| rs1718845 | Gastric cancer | 4 | 57943153 | G/A | 0.734 | 0.017 | 0.022 | 0.438 | 31.755 |
| rs114282228 | Gastric cancer | 4 | 57797467 | T/C | 0.020 | 0.016 | 0.161 | 0.919 | 33.962 |
| rs10012574 | Gastric cancer | 4 | 57747276 | T/C | 0.479 | 0.015 | 0.016 | 0.333 | 510.698 |
| rs781570 | Gastric cancer | 4 | 57705999 | C/A | 0.846 | 0.014 | 0.020 | 0.491 | 28.079 |
| rs75522131 | Gastric cancer | 4 | 57966325 | A/G | 0.142 | 0.012 | 0.080 | 0.883 | 57.874 |
| rs113895998 | Gastric cancer | 4 | 57696235 | A/G | 0.018 | 0.010 | 0.168 | 0.951 | 28.089 |
| rs4865181 | Gastric cancer | 4 | 57963469 | A/G | 0.283 | 0.010 | 0.017 | 0.541 | 456.647 |
| rs111632193 | Gastric cancer | 4 | 57698525 | A/C | 0.155 | 0.010 | 0.028 | 0.716 | 42.366 |
| rs6828212 | Gastric cancer | 4 | 58026173 | A/C | 0.392 | 0.009 | 0.019 | 0.639 | 25.228 |
| rs13108002 | Gastric cancer | 4 | 57718253 | T/C | 0.330 | 0.006 | 0.024 | 0.797 | 152.816 |
| rs2271808 | Gastric cancer | 4 | 57899602 | T/C | 0.356 | 0.005 | 0.017 | 0.765 | 640.153 |
| rs1718860 | Gastric cancer | 4 | 57949517 | A/G | 0.712 | 0.004 | 0.017 | 0.835 | 42.848 |
| rs11573031 | Gastric cancer | 4 | 57965669 | A/G | 0.023 | 0.003 | 0.145 | 0.983 | 50.111 |
| rs67057259 | Gastric cancer | 4 | 57958652 | G/A | 0.162 | 0.003 | 0.061 | 0.966 | 200.407 |
| rs781550 | Gastric cancer | 4 | 57729256 | A/G | 0.088 | 0.002 | 0.030 | 0.937 | 59.607 |
| rs4611987 | Gastric cancer | 4 | 57923981 | G/A | 0.860 | 0.001 | 0.032 | 0.969 | 150.531 |
| rs1713957 | Gastric cancer | 4 | 57923517 | A/G | 0.448 | 0.001 | 0.016 | 0.961 | 583.068 |
| rs144563685 | Gastric cancer | 4 | 58026454 | C/T | 0.196 | -0.001 | 0.022 | 0.951 | 28.041 |
| rs59562605 | Gastric cancer | 4 | 57912040 | T/C | 0.154 | -0.003 | 0.018 | 0.885 | 49.083 |
| rs1277292 | Gastric cancer | 4 | 57931613 | A/C | 0.496 | -0.003 | 0.016 | 0.866 | 185.695 |
| rs35867715 | Gastric cancer | 4 | 57627999 | C/T | 0.264 | -0.004 | 0.019 | 0.812 | 32.992 |
| rs11573079 | Gastric cancer | 4 | 57929631 | A/C | 0.037 | -0.005 | 0.028 | 0.870 | 34.052 |
| rs6812026 | Gastric cancer | 4 | 57981902 | A/C | 0.214 | -0.005 | 0.017 | 0.771 | 253.462 |
| rs1277305 | Gastric cancer | 4 | 57792128 | A/G | 0.525 | -0.008 | 0.017 | 0.651 | 265.992 |
| rs1714020 | Gastric cancer | 4 | 57937134 | C/T | 0.771 | -0.008 | 0.018 | 0.665 | 106.905 |
| rs10434431 | Gastric cancer | 4 | 58030307 | G/A | 0.336 | -0.008 | 0.018 | 0.664 | 24.797 |
| rs114150987 | Gastric cancer | 4 | 58114577 | C/A | 0.081 | -0.009 | 0.035 | 0.791 | 32.210 |
| rs55987498 | Gastric cancer | 4 | 58019178 | T/C | 0.140 | -0.010 | 0.021 | 0.652 | 75.523 |
| rs62308299 | Gastric cancer | 4 | 57981582 | T/C | 0.038 | -0.010 | 0.047 | 0.840 | 68.510 |
| rs7670481 | Gastric cancer | 4 | 58001688 | T/C | 0.485 | -0.011 | 0.017 | 0.526 | 80.000 |
| rs1708763 | Gastric cancer | 4 | 57737041 | G/A | 0.466 | -0.011 | 0.017 | 0.512 | 42.134 |
| rs11573053 | Gastric cancer | 4 | 57942825 | G/A | 0.192 | -0.011 | 0.024 | 0.636 | 138.914 |
| rs60399011 | Gastric cancer | 4 | 57749875 | T/C | 0.136 | -0.012 | 0.019 | 0.518 | 41.696 |
| rs11732382 | Gastric cancer | 4 | 57661555 | G/A | 0.268 | -0.015 | 0.050 | 0.762 | 24.557 |
| rs7677987 | Gastric cancer | 4 | 58020760 | A/G | 0.249 | -0.018 | 0.052 | 0.730 | 36.896 |
| rs10005320 | Gastric cancer | 4 | 57720234 | T/C | 0.083 | -0.019 | 0.027 | 0.485 | 46.559 |
| rs62308335 | Gastric cancer | 4 | 58031176 | A/G | 0.107 | -0.020 | 0.023 | 0.393 | 24.273 |
| rs9760540 | Gastric cancer | 4 | 57741620 | G/A | 0.127 | -0.020 | 0.040 | 0.617 | 411.300 |
| rs6814936 | Gastric cancer | 4 | 57635503 | A/G | 0.071 | -0.021 | 0.028 | 0.442 | 48.295 |
| rs1713949 | Gastric cancer | 4 | 57849764 | C/T | 0.925 | -0.023 | 0.047 | 0.620 | 94.846 |
| rs67241701 | Gastric cancer | 4 | 58027169 | G/T | 0.076 | -0.028 | 0.029 | 0.330 | 20.614 |
| rs3755905 | Gastric cancer | 4 | 57906930 | G/T | 0.067 | -0.037 | 0.025 | 0.132 | 52.059 |
| rs140471527 | Gastric cancer | 4 | 57785350 | T/C | 0.037 | -0.037 | 0.118 | 0.751 | 41.088 |
| rs1718840 | Gastric cancer | 4 | 57827171 | A/G | 0.046 | -0.040 | 0.106 | 0.703 | 74.918 |
| rs34862525 | Gastric cancer | 4 | 57665826 | T/C | 0.084 | -0.043 | 0.080 | 0.590 | 33.278 |
| rs34713282 | Gastric cancer | 4 | 58005775 | A/G | 0.049 | -0.044 | 0.103 | 0.667 | 38.686 |
| rs78440444 | Gastric cancer | 4 | 57818637 | A/G | 0.057 | -0.045 | 0.097 | 0.646 | 142.180 |
| rs75882609 | Gastric cancer | 4 | 57950739 | G/T | 0.074 | -0.045 | 0.087 | 0.603 | 161.246 |
| rs17787430 | Gastric cancer | 4 | 57929982 | T/G | 0.075 | -0.056 | 0.044 | 0.203 | 302.897 |
| rs116686317 | Gastric cancer | 4 | 57903239 | G/A | 0.039 | -0.067 | 0.114 | 0.557 | 46.032 |
| rs73242635 | Gastric cancer | 4 | 57935733 | A/G | 0.032 | -0.068 | 0.103 | 0.508 | 26.869 |
| rs6839753 | Gastric cancer | 4 | 58098768 | A/G | 0.297 | -0.105 | 0.061 | 0.086 | 28.809 |
| rs111538253 | Gastric cancer | 4 | 57937693 | T/C | 0.015 | -0.107 | 0.182 | 0.556 | 40.229 |
| rs62309920 | Gastric cancer | 4 | 57602042 | G/T | 0.024 | -0.134 | 0.152 | 0.379 | 30.543 |
| rs112943852 | Gastric cancer | 4 | 57686073 | A/G | 0.039 | -0.206 | 0.115 | 0.072 | 23.414 |
| rs62310297 | Gastric cancer | 4 | 57872766 | T/C | 0.022 | -0.245 | 0.151 | 0.104 | 47.050 |
| rs11573041 | Gastric cancer | 4 | 57948316 | T/G | 0.018 | -0.335 | 0.166 | 0.044 | 51.556 |
| PPAT |  |  |  |  |  |  |  |  |  |
| rs141459401 | Gastric cancer | 4 | 57251845 | A/C | 0.083 | 0.202 | 0.083 | 0.015 | 30.834 |
| rs56719128 | Gastric cancer | 4 | 57427143 | A/C | 0.214 | 0.041 | 0.019 | 0.028 | 22.066 |
| rs17086758 | Gastric cancer | 4 | 57280554 | C/T | 0.042 | 0.041 | 0.035 | 0.242 | 28.839 |
| rs59467381 | Gastric cancer | 4 | 57404019 | T/C | 0.085 | 0.019 | 0.031 | 0.538 | 26.980 |
| rs113089454 | Gastric cancer | 4 | 57451567 | T/C | 0.031 | 0.001 | 0.130 | 0.996 | 21.048 |
| rs1401906 | Gastric cancer | 4 | 57187415 | C/T | 0.476 | 0.000 | 0.018 | 0.980 | 160.997 |
| rs4131086 | Gastric cancer | 4 | 57385455 | G/A | 0.388 | -0.011 | 0.016 | 0.480 | 41.574 |
| rs1464286 | Gastric cancer | 4 | 57290126 | G/A | 0.688 | -0.016 | 0.017 | 0.333 | 102.394 |
| rs10010544 | Gastric cancer | 4 | 57202676 | T/C | 0.132 | -0.019 | 0.023 | 0.423 | 112.478 |
| rs4362880 | Gastric cancer | 4 | 57316066 | A/G | 0.027 | -0.021 | 0.141 | 0.884 | 33.532 |
| rs28470886 | Gastric cancer | 4 | 57397082 | C/T | 0.304 | -0.046 | 0.016 | 0.005 | 65.587 |
| EXOC1 |  |  |  |  |  |  |  |  |  |
| rs143147253 | Gastric cancer | 4 | 56661477 | A/G | 0.016 | 0.219 | 0.187 | 0.242 | 21.257 |
| rs2412693 | Gastric cancer | 4 | 56672709 | G/A | 0.214 | 0.023 | 0.022 | 0.293 | 25.220 |
| rs7679852 | Gastric cancer | 4 | 56683846 | C/T | 0.451 | 0.016 | 0.019 | 0.373 | 34.751 |
| rs12152592 | Gastric cancer | 4 | 56663194 | T/C | 0.441 | 0.013 | 0.020 | 0.531 | 22.527 |
| rs968446 | Gastric cancer | 4 | 56660386 | T/C | 0.246 | 0.009 | 0.017 | 0.585 | 20.686 |
| rs73238392 | Gastric cancer | 4 | 56761931 | T/C | 0.200 | 0.008 | 0.016 | 0.641 | 70.254 |
| rs7695702 | Gastric cancer | 4 | 56637704 | T/C | 0.789 | 0.006 | 0.034 | 0.856 | 22.416 |
| rs76263457 | Gastric cancer | 4 | 56643574 | G/A | 0.108 | 0.002 | 0.018 | 0.916 | 20.615 |
| rs10015409 | Gastric cancer | 4 | 56641023 | C/A | 0.345 | 0.001 | 0.016 | 0.933 | 34.003 |
| rs28759770 | Gastric cancer | 4 | 56600956 | G/T | 0.609 | -0.003 | 0.022 | 0.907 | 32.047 |
| rs7660872 | Gastric cancer | 4 | 56903251 | T/C | 0.216 | -0.050 | 0.029 | 0.088 | 33.638 |
| rs28660976 | Gastric cancer | 4 | 56617282 | C/T | 0.546 | -0.058 | 0.057 | 0.306 | 21.212 |
| rs6841695 | Gastric cancer | 4 | 56872528 | C/T | 0.064 | -0.076 | 0.040 | 0.060 | 44.852 |
| rs76653299 | Gastric cancer | 4 | 56895326 | A/C | 0.035 | -0.078 | 0.120 | 0.516 | 24.174 |
| rs11726168 | Gastric cancer | 4 | 56595706 | C/T | 0.117 | -0.090 | 0.069 | 0.194 | 51.523 |
| rs17783479 | Gastric cancer | 4 | 56599416 | C/T | 0.040 | -0.222 | 0.114 | 0.052 | 21.829 |
| AIMP1 |  |  |  |  |  |  |  |  |  |
| rs114933695 | Gastric cancer | 4 | 107178698 | T/C | 0.028 | 0.311 | 0.135 | 0.021 | 20.846 |
| rs79150233 | Gastric cancer | 4 | 107047744 | G/T | 0.047 | 0.137 | 0.106 | 0.195 | 20.659 |
| rs7683106 | Gastric cancer | 4 | 106970272 | A/C | 0.145 | 0.050 | 0.063 | 0.429 | 29.983 |
| rs10016884 | Gastric cancer | 4 | 107120677 | C/T | 0.717 | 0.018 | 0.016 | 0.263 | 149.473 |
| rs6856408 | Gastric cancer | 4 | 107236868 | G/A | 0.189 | 0.011 | 0.019 | 0.577 | 143.132 |
| rs12513317 | Gastric cancer | 4 | 106975067 | T/C | 0.187 | 0.010 | 0.020 | 0.593 | 135.776 |
| rs13126551 | Gastric cancer | 4 | 107297522 | G/A | 0.481 | 0.002 | 0.017 | 0.910 | 41.940 |
| rs9685568 | Gastric cancer | 4 | 107386029 | C/A | 0.176 | -0.003 | 0.021 | 0.905 | 26.241 |
| rs114393400 | Gastric cancer | 4 | 107039586 | G/T | 0.032 | -0.041 | 0.044 | 0.351 | 33.904 |
| rs28722963 | Gastric cancer | 4 | 107232881 | C/T | 0.081 | -0.078 | 0.082 | 0.341 | 31.731 |
| rs56220887 | Gastric cancer | 4 | 107202536 | G/T | 0.027 | -0.300 | 0.140 | 0.032 | 24.999 |
| DCK |  |  |  |  |  |  |  |  |  |
| rs116029892 | Gastric cancer | 4 | 71718454 | A/G | 0.023 | 0.332 | 0.155 | 0.032 | 44.907 |
| rs16845887 | Gastric cancer | 4 | 72005823 | G/A | 0.019 | 0.312 | 0.165 | 0.058 | 73.933 |
| rs10019874 | Gastric cancer | 4 | 71861270 | C/T | 0.021 | 0.204 | 0.153 | 0.183 | 375.499 |
| rs62325392 | Gastric cancer | 4 | 71790241 | A/G | 0.082 | 0.163 | 0.085 | 0.057 | 31.240 |
| rs141975383 | Gastric cancer | 4 | 71822740 | A/G | 0.014 | 0.153 | 0.191 | 0.423 | 21.759 |
| rs140915056 | Gastric cancer | 4 | 71651406 | A/C | 0.019 | 0.126 | 0.162 | 0.439 | 402.593 |
| rs59194778 | Gastric cancer | 4 | 72021413 | G/T | 0.068 | 0.063 | 0.034 | 0.064 | 39.129 |
| rs140515517 | Gastric cancer | 4 | 71863004 | T/C | 0.014 | 0.062 | 0.199 | 0.754 | 60.811 |
| rs2363719 | Gastric cancer | 4 | 72138216 | A/G | 0.121 | 0.034 | 0.024 | 0.153 | 26.183 |
| rs17688758 | Gastric cancer | 4 | 71997259 | C/T | 0.103 | 0.013 | 0.021 | 0.557 | 54.004 |
| rs1984848 | Gastric cancer | 4 | 72004709 | C/T | 0.121 | 0.012 | 0.025 | 0.648 | 39.940 |
| rs28480090 | Gastric cancer | 4 | 71975492 | T/C | 0.179 | 0.005 | 0.018 | 0.801 | 23.851 |
| rs12512502 | Gastric cancer | 4 | 71776935 | C/A | 0.603 | -0.003 | 0.017 | 0.862 | 50.607 |
| rs1043903 | Gastric cancer | 4 | 71672653 | G/A | 0.389 | -0.004 | 0.016 | 0.790 | 69.401 |
| rs58406098 | Gastric cancer | 4 | 71792495 | C/T | 0.050 | -0.007 | 0.023 | 0.770 | 25.300 |
| rs1891451 | Gastric cancer | 4 | 71570134 | A/G | 0.552 | -0.009 | 0.018 | 0.612 | 38.686 |
| rs6845656 | Gastric cancer | 4 | 71741844 | A/C | 0.880 | -0.011 | 0.020 | 0.587 | 70.069 |
| rs10029546 | Gastric cancer | 4 | 72016768 | G/A | 0.646 | -0.023 | 0.019 | 0.230 | 22.921 |
| rs6447020 | Gastric cancer | 4 | 71965223 | A/G | 0.975 | -0.135 | 0.147 | 0.361 | 207.311 |
| YTHDC2 |  |  |  |  |  |  |  |  |  |
| rs72810022 | Gastric cancer | 5 | 113149169 | G/A | 0.020 | 0.289 | 0.167 | 0.085 | 34.949 |
| rs113998419 | Gastric cancer | 5 | 112731255 | C/T | 0.025 | 0.189 | 0.142 | 0.183 | 30.634 |
| rs114959290 | Gastric cancer | 5 | 112841809 | C/T | 0.043 | 0.133 | 0.109 | 0.223 | 85.915 |
| rs112266112 | Gastric cancer | 5 | 112961420 | A/G | 0.032 | 0.106 | 0.127 | 0.405 | 20.808 |
| rs72810007 | Gastric cancer | 5 | 113125527 | G/A | 0.049 | 0.081 | 0.049 | 0.096 | 31.969 |
| rs10071403 | Gastric cancer | 5 | 113216633 | C/T | 0.071 | 0.081 | 0.086 | 0.352 | 37.529 |
| rs79473751 | Gastric cancer | 5 | 112905479 | T/C | 0.065 | 0.064 | 0.033 | 0.049 | 44.265 |
| rs79267814 | Gastric cancer | 5 | 112783921 | A/C | 0.015 | 0.064 | 0.188 | 0.733 | 37.622 |
| rs76982909 | Gastric cancer | 5 | 112801564 | G/A | 0.055 | 0.064 | 0.032 | 0.044 | 34.275 |
| rs35019626 | Gastric cancer | 5 | 113124606 | A/G | 0.620 | 0.057 | 0.019 | 0.003 | 34.823 |
| rs1482346 | Gastric cancer | 5 | 113053318 | G/A | 0.815 | 0.056 | 0.031 | 0.070 | 132.007 |
| rs74999043 | Gastric cancer | 5 | 112724675 | G/A | 0.017 | 0.055 | 0.177 | 0.756 | 23.817 |
| rs2195367 | Gastric cancer | 5 | 113012575 | G/A | 0.902 | 0.049 | 0.047 | 0.300 | 79.431 |
| rs72788615 | Gastric cancer | 5 | 112585908 | T/G | 0.062 | 0.045 | 0.092 | 0.627 | 252.444 |
| rs115303527 | Gastric cancer | 5 | 112684506 | C/T | 0.037 | 0.035 | 0.121 | 0.770 | 23.978 |
| rs10051648 | Gastric cancer | 5 | 112980424 | C/T | 0.158 | 0.035 | 0.030 | 0.253 | 29.939 |
| rs111974768 | Gastric cancer | 5 | 112793843 | T/C | 0.062 | 0.033 | 0.093 | 0.720 | 265.376 |
| rs149921468 | Gastric cancer | 5 | 112880264 | C/T | 0.022 | 0.031 | 0.155 | 0.841 | 29.920 |
| rs4492103 | Gastric cancer | 5 | 113041312 | T/C | 0.704 | 0.030 | 0.018 | 0.098 | 67.467 |
| rs2170527 | Gastric cancer | 5 | 113118517 | A/C | 0.780 | 0.026 | 0.031 | 0.393 | 20.671 |
| rs17135735 | Gastric cancer | 5 | 112917803 | T/C | 0.068 | 0.026 | 0.089 | 0.770 | 248.422 |
| rs148024110 | Gastric cancer | 5 | 113062801 | A/C | 0.441 | 0.024 | 0.017 | 0.145 | 156.036 |
| rs34561076 | Gastric cancer | 5 | 112723012 | A/G | 0.167 | 0.017 | 0.027 | 0.522 | 205.903 |
| rs75047936 | Gastric cancer | 5 | 113007312 | T/G | 0.033 | 0.017 | 0.035 | 0.628 | 197.130 |
| rs182987 | Gastric cancer | 5 | 112839026 | G/A | 0.538 | 0.016 | 0.018 | 0.360 | 610.374 |
| rs2128313 | Gastric cancer | 5 | 113118069 | G/A | 0.632 | 0.015 | 0.017 | 0.382 | 41.524 |
| rs7705632 | Gastric cancer | 5 | 113096022 | G/A | 0.009 | 0.015 | 0.234 | 0.950 | 35.263 |
| rs114060237 | Gastric cancer | 5 | 113121593 | G/A | 0.078 | 0.012 | 0.084 | 0.885 | 23.899 |
| rs7724917 | Gastric cancer | 5 | 112582714 | T/C | 0.296 | 0.009 | 0.019 | 0.651 | 661.755 |
| rs78898148 | Gastric cancer | 5 | 112950740 | G/A | 0.016 | 0.009 | 0.184 | 0.963 | 23.449 |
| rs10478118 | Gastric cancer | 5 | 112644247 | C/T | 0.041 | 0.003 | 0.068 | 0.963 | 96.519 |
| rs67214532 | Gastric cancer | 5 | 113206216 | C/T | 0.195 | 0.003 | 0.035 | 0.944 | 21.633 |
| rs115386141 | Gastric cancer | 5 | 112893477 | G/A | 0.044 | 0.001 | 0.112 | 0.993 | 49.087 |
| rs509237 | Gastric cancer | 5 | 112550247 | G/A | 0.775 | -0.004 | 0.018 | 0.840 | 477.750 |
| rs6594702 | Gastric cancer | 5 | 112691960 | T/C | 0.046 | -0.014 | 0.038 | 0.710 | 507.570 |
| rs11241245 | Gastric cancer | 5 | 113193525 | G/T | 0.154 | -0.015 | 0.020 | 0.460 | 28.139 |
| rs10477493 | Gastric cancer | 5 | 112818821 | T/C | 0.046 | -0.015 | 0.038 | 0.692 | 532.502 |
| rs57134656 | Gastric cancer | 5 | 112864239 | A/C | 0.191 | -0.015 | 0.021 | 0.473 | 1053.225 |
| rs10063334 | Gastric cancer | 5 | 112760874 | C/T | 0.172 | -0.016 | 0.022 | 0.452 | 931.346 |
| rs11950886 | Gastric cancer | 5 | 112937133 | T/C | 0.047 | -0.017 | 0.037 | 0.641 | 540.225 |
| rs1845290 | Gastric cancer | 5 | 112687214 | A/G | 0.458 | -0.019 | 0.018 | 0.291 | 532.823 |
| rs12515653 | Gastric cancer | 5 | 113007302 | T/G | 0.225 | -0.020 | 0.017 | 0.249 | 29.692 |
| rs900303 | Gastric cancer | 5 | 113129950 | A/G | 0.836 | -0.020 | 0.026 | 0.436 | 54.752 |
| rs10478145 | Gastric cancer | 5 | 113113879 | A/G | 0.090 | -0.022 | 0.020 | 0.259 | 22.802 |
| rs139822431 | Gastric cancer | 5 | 112935672 | T/C | 0.047 | -0.024 | 0.026 | 0.362 | 66.975 |
| rs60097481 | Gastric cancer | 5 | 112575173 | A/G | 0.066 | -0.025 | 0.033 | 0.451 | 228.505 |
| rs2195369 | Gastric cancer | 5 | 113009441 | C/A | 0.437 | -0.025 | 0.016 | 0.128 | 22.751 |
| rs72810019 | Gastric cancer | 5 | 113147967 | G/T | 0.050 | -0.026 | 0.106 | 0.806 | 33.055 |
| rs73245079 | Gastric cancer | 5 | 112978061 | T/C | 0.045 | -0.028 | 0.041 | 0.505 | 116.635 |
| rs10043198 | Gastric cancer | 5 | 113120219 | T/C | 0.099 | -0.031 | 0.022 | 0.148 | 46.667 |
| rs10072577 | Gastric cancer | 5 | 112879789 | A/G | 0.873 | -0.036 | 0.035 | 0.303 | 80.262 |
| rs1822488 | Gastric cancer | 5 | 112604760 | T/C | 0.083 | -0.039 | 0.028 | 0.171 | 258.085 |
| rs1838204 | Gastric cancer | 5 | 112644349 | C/T | 0.250 | -0.039 | 0.021 | 0.067 | 58.052 |
| rs116292983 | Gastric cancer | 5 | 112757833 | C/T | 0.055 | -0.041 | 0.101 | 0.683 | 61.340 |
| rs11241203 | Gastric cancer | 5 | 112693442 | A/G | 0.213 | -0.043 | 0.055 | 0.428 | 160.830 |
| rs11956333 | Gastric cancer | 5 | 112969748 | C/T | 0.009 | -0.044 | 0.234 | 0.849 | 60.502 |
| rs13355627 | Gastric cancer | 5 | 112572840 | T/C | 0.056 | -0.060 | 0.034 | 0.078 | 430.619 |
| rs4583881 | Gastric cancer | 5 | 113124757 | G/A | 0.143 | -0.060 | 0.066 | 0.366 | 20.900 |
| rs1816062 | Gastric cancer | 5 | 112856023 | C/A | 0.149 | -0.063 | 0.031 | 0.040 | 33.942 |
| rs72810020 | Gastric cancer | 5 | 113148083 | T/G | 0.014 | -0.063 | 0.130 | 0.626 | 50.583 |
| rs4705804 | Gastric cancer | 5 | 112558770 | C/T | 0.752 | -0.073 | 0.022 | 0.001 | 172.423 |
| rs6864965 | Gastric cancer | 5 | 113124189 | C/T | 0.112 | -0.087 | 0.072 | 0.230 | 24.738 |
| rs2195375 | Gastric cancer | 5 | 112988579 | T/C | 0.080 | -0.090 | 0.084 | 0.280 | 28.782 |
| rs62382054 | Gastric cancer | 5 | 113121298 | G/A | 0.047 | -0.099 | 0.111 | 0.372 | 24.003 |
| rs80107724 | Gastric cancer | 5 | 112717759 | A/G | 0.051 | -0.111 | 0.102 | 0.280 | 32.282 |
| rs114514314 | Gastric cancer | 5 | 112568083 | C/T | 0.008 | -0.139 | 0.249 | 0.576 | 21.938 |
| rs35676551 | Gastric cancer | 5 | 112553563 | A/C | 0.062 | -0.162 | 0.097 | 0.094 | 29.574 |
| rs72794123 | Gastric cancer | 5 | 112868785 | G/A | 0.019 | -0.166 | 0.168 | 0.324 | 96.963 |
| rs75793391 | Gastric cancer | 5 | 112887936 | G/A | 0.032 | -0.166 | 0.135 | 0.217 | 42.654 |
| rs77778476 | Gastric cancer | 5 | 113149626 | T/C | 0.011 | -0.238 | 0.214 | 0.266 | 42.657 |
| rs113816144 | Gastric cancer | 5 | 113066351 | T/G | 0.015 | -0.364 | 0.183 | 0.046 | 43.095 |
| rs112744876 | Gastric cancer | 5 | 112751818 | G/A | 0.012 | -0.473 | 0.206 | 0.022 | 106.612 |
| rs142231548 | Gastric cancer | 5 | 112906433 | T/C | 0.012 | -0.510 | 0.207 | 0.014 | 111.077 |
| LHFPL2 |  |  |  |  |  |  |  |  |  |
| rs17801606 | Gastric cancer | 5 | 77720720 | C/A | 0.021 | 0.038 | 0.071 | 0.593 | 32.372 |
| rs4704514 | Gastric cancer | 5 | 77820081 | T/C | 0.287 | 0.033 | 0.018 | 0.063 | 26.722 |
| rs10462558 | Gastric cancer | 5 | 77806988 | G/T | 0.103 | 0.027 | 0.018 | 0.137 | 295.389 |
| rs11743650 | Gastric cancer | 5 | 77783029 | C/T | 0.238 | 0.017 | 0.034 | 0.607 | 59.698 |
| rs1968382 | Gastric cancer | 5 | 77721404 | C/A | 0.421 | 0.015 | 0.021 | 0.475 | 466.552 |
| rs1035856 | Gastric cancer | 5 | 77916348 | A/G | 0.099 | 0.010 | 0.019 | 0.589 | 119.082 |
| rs344651 | Gastric cancer | 5 | 77852998 | C/T | 0.258 | 0.007 | 0.016 | 0.685 | 182.782 |
| rs4133340 | Gastric cancer | 5 | 77518914 | G/A | 0.775 | 0.005 | 0.017 | 0.787 | 21.763 |
| rs149265908 | Gastric cancer | 5 | 77981973 | C/T | 0.728 | 0.002 | 0.018 | 0.921 | 22.911 |
| rs6881634 | Gastric cancer | 5 | 77630854 | A/G | 0.475 | 0.000 | 0.024 | 0.996 | 99.428 |
| rs6875879 | Gastric cancer | 5 | 77812753 | A/G | 0.307 | -0.001 | 0.016 | 0.954 | 50.972 |
| rs17217297 | Gastric cancer | 5 | 78024610 | G/A | 0.342 | -0.006 | 0.047 | 0.905 | 25.586 |
| rs341927 | Gastric cancer | 5 | 77940438 | T/C | 0.534 | -0.010 | 0.016 | 0.546 | 56.683 |
| rs11749979 | Gastric cancer | 5 | 77640190 | C/T | 0.414 | -0.019 | 0.016 | 0.227 | 20.201 |
| rs4455545 | Gastric cancer | 5 | 77639605 | T/G | 0.201 | -0.021 | 0.016 | 0.202 | 109.391 |
| rs145430413 | Gastric cancer | 5 | 77722815 | G/A | 0.019 | -0.026 | 0.165 | 0.877 | 30.648 |
| rs72760999 | Gastric cancer | 5 | 77918480 | A/G | 0.037 | -0.026 | 0.121 | 0.832 | 39.373 |
| rs4235707 | Gastric cancer | 5 | 77669593 | G/A | 0.883 | -0.026 | 0.017 | 0.128 | 209.372 |
| rs9293756 | Gastric cancer | 5 | 77918136 | C/A | 0.385 | -0.027 | 0.019 | 0.167 | 233.387 |
| rs34514484 | Gastric cancer | 5 | 77648297 | A/G | 0.170 | -0.027 | 0.031 | 0.371 | 185.693 |
| rs9293744 | Gastric cancer | 5 | 77637023 | C/T | 0.241 | -0.029 | 0.032 | 0.354 | 35.730 |
| rs138800755 | Gastric cancer | 5 | 77962505 | C/T | 0.020 | -0.030 | 0.164 | 0.854 | 25.850 |
| rs62362576 | Gastric cancer | 5 | 77645045 | T/C | 0.067 | -0.034 | 0.091 | 0.707 | 40.357 |
| rs7721185 | Gastric cancer | 5 | 77515737 | G/A | 0.244 | -0.036 | 0.028 | 0.194 | 42.397 |
| rs78214046 | Gastric cancer | 5 | 77763157 | G/A | 0.023 | -0.041 | 0.149 | 0.786 | 21.564 |
| rs340079 | Gastric cancer | 5 | 77992284 | C/A | 0.493 | -0.049 | 0.020 | 0.014 | 29.724 |
| rs2885809 | Gastric cancer | 5 | 78018768 | A/G | 0.381 | -0.053 | 0.047 | 0.259 | 34.515 |
| rs115492025 | Gastric cancer | 5 | 77832116 | T/C | 0.020 | -0.059 | 0.163 | 0.719 | 20.530 |
| rs10052968 | Gastric cancer | 5 | 77903262 | C/T | 0.191 | -0.060 | 0.057 | 0.286 | 166.150 |
| rs7356739 | Gastric cancer | 5 | 77622868 | G/A | 0.264 | -0.069 | 0.050 | 0.172 | 45.044 |
| rs144535168 | Gastric cancer | 5 | 77785380 | A/C | 0.018 | -0.111 | 0.171 | 0.515 | 44.604 |
| rs62362623 | Gastric cancer | 5 | 77696631 | C/T | 0.072 | -0.121 | 0.087 | 0.164 | 74.629 |
| MTFR1 |  |  |  |  |  |  |  |  |  |
| rs139594272 | Gastric cancer | 8 | 66792102 | G/A | 0.009 | 0.418 | 0.285 | 0.142 | 38.943 |
| rs151273849 | Gastric cancer | 8 | 66509509 | A/G | 0.015 | 0.336 | 0.184 | 0.067 | 28.569 |
| rs34369294 | Gastric cancer | 8 | 66485512 | C/T | 0.055 | 0.225 | 0.098 | 0.022 | 41.542 |
| rs189480653 | Gastric cancer | 8 | 66351364 | A/G | 0.023 | 0.193 | 0.157 | 0.217 | 109.611 |
| rs150693821 | Gastric cancer | 8 | 66716975 | C/T | 0.021 | 0.163 | 0.157 | 0.300 | 191.531 |
| rs146494279 | Gastric cancer | 8 | 66668611 | C/A | 0.022 | 0.156 | 0.155 | 0.314 | 327.661 |
| rs118152223 | Gastric cancer | 8 | 66792908 | A/G | 0.023 | 0.149 | 0.150 | 0.322 | 218.764 |
| rs141038879 | Gastric cancer | 8 | 66398165 | A/G | 0.019 | 0.143 | 0.162 | 0.378 | 345.692 |
| rs74616255 | Gastric cancer | 8 | 66588465 | C/A | 0.069 | 0.123 | 0.089 | 0.166 | 1551.621 |
| rs116877959 | Gastric cancer | 8 | 66260703 | A/G | 0.038 | 0.110 | 0.099 | 0.266 | 24.131 |
| rs73251319 | Gastric cancer | 8 | 66378692 | T/G | 0.022 | 0.110 | 0.075 | 0.140 | 69.893 |
| rs11774665 | Gastric cancer | 8 | 66320669 | T/C | 0.042 | 0.100 | 0.116 | 0.390 | 25.437 |
| rs72664750 | Gastric cancer | 8 | 66429778 | G/T | 0.056 | 0.098 | 0.080 | 0.220 | 963.108 |
| rs141623092 | Gastric cancer | 8 | 66546130 | A/C | 0.013 | 0.097 | 0.204 | 0.636 | 292.291 |
| rs17397411 | Gastric cancer | 8 | 66904773 | A/C | 0.103 | 0.092 | 0.074 | 0.214 | 48.542 |
| rs10089133 | Gastric cancer | 8 | 66320587 | A/G | 0.935 | 0.089 | 0.090 | 0.327 | 62.720 |
| rs180758063 | Gastric cancer | 8 | 66461261 | T/C | 0.058 | 0.086 | 0.097 | 0.377 | 1017.402 |
| rs189386861 | Gastric cancer | 8 | 66389950 | A/G | 0.011 | 0.084 | 0.219 | 0.703 | 23.908 |
| rs74805159 | Gastric cancer | 8 | 66680248 | A/G | 0.063 | 0.082 | 0.100 | 0.411 | 51.265 |
| rs117336177 | Gastric cancer | 8 | 66701846 | C/T | 0.018 | 0.081 | 0.171 | 0.637 | 20.143 |
| rs62507652 | Gastric cancer | 8 | 66736720 | G/A | 0.053 | 0.075 | 0.099 | 0.446 | 26.405 |
| rs184640020 | Gastric cancer | 8 | 66529643 | C/A | 0.023 | 0.069 | 0.042 | 0.105 | 117.451 |
| rs59770832 | Gastric cancer | 8 | 66357258 | G/A | 0.190 | 0.061 | 0.029 | 0.037 | 257.863 |
| rs77784867 | Gastric cancer | 8 | 66754497 | T/C | 0.027 | 0.061 | 0.042 | 0.144 | 64.343 |
| rs2357395 | Gastric cancer | 8 | 66315494 | C/T | 0.321 | 0.055 | 0.044 | 0.216 | 74.036 |
| rs2357478 | Gastric cancer | 8 | 66610806 | A/G | 0.182 | 0.054 | 0.028 | 0.051 | 1383.217 |
| rs78780238 | Gastric cancer | 8 | 66637901 | A/G | 0.023 | 0.053 | 0.042 | 0.206 | 101.816 |
| rs189745612 | Gastric cancer | 8 | 66520619 | T/C | 0.026 | 0.048 | 0.141 | 0.737 | 28.882 |
| rs138720756 | Gastric cancer | 8 | 66480037 | T/C | 0.014 | 0.046 | 0.195 | 0.813 | 274.734 |
| rs28521470 | Gastric cancer | 8 | 66850258 | C/T | 0.027 | 0.039 | 0.138 | 0.779 | 29.525 |
| rs4737751 | Gastric cancer | 8 | 66600233 | G/A | 0.887 | 0.039 | 0.025 | 0.125 | 32.518 |
| rs72666509 | Gastric cancer | 8 | 66550599 | A/G | 0.066 | 0.036 | 0.078 | 0.643 | 86.012 |
| rs117587105 | Gastric cancer | 8 | 66672347 | T/C | 0.039 | 0.032 | 0.117 | 0.783 | 37.554 |
| rs78748261 | Gastric cancer | 8 | 66625124 | A/G | 0.067 | 0.032 | 0.039 | 0.411 | 346.840 |
| rs71529172 | Gastric cancer | 8 | 66431728 | C/A | 0.027 | 0.031 | 0.139 | 0.821 | 25.978 |
| rs72664801 | Gastric cancer | 8 | 66542437 | C/T | 0.072 | 0.021 | 0.086 | 0.810 | 1515.885 |
| rs12545181 | Gastric cancer | 8 | 66326001 | A/G | 0.379 | 0.021 | 0.017 | 0.240 | 79.548 |
| rs143568834 | Gastric cancer | 8 | 66839800 | C/T | 0.019 | 0.019 | 0.165 | 0.908 | 167.866 |
| rs1108213 | Gastric cancer | 8 | 66897815 | T/C | 0.147 | 0.018 | 0.028 | 0.537 | 936.756 |
| rs4294622 | Gastric cancer | 8 | 66480563 | C/A | 0.352 | 0.013 | 0.016 | 0.412 | 1190.445 |
| rs147656703 | Gastric cancer | 8 | 66543658 | A/G | 0.087 | 0.011 | 0.078 | 0.888 | 21.923 |
| rs17304935 | Gastric cancer | 8 | 66679221 | C/A | 0.239 | 0.008 | 0.020 | 0.685 | 910.568 |
| rs77732976 | Gastric cancer | 8 | 66684800 | T/C | 0.060 | 0.008 | 0.094 | 0.933 | 954.097 |
| rs11781358 | Gastric cancer | 8 | 66469926 | C/T | 0.459 | 0.006 | 0.045 | 0.897 | 232.741 |
| rs4737738 | Gastric cancer | 8 | 66452315 | G/A | 0.795 | 0.005 | 0.018 | 0.757 | 125.890 |
| rs9650151 | Gastric cancer | 8 | 66628852 | A/G | 0.333 | 0.005 | 0.044 | 0.903 | 525.314 |
| rs62507572 | Gastric cancer | 8 | 66470743 | A/G | 0.591 | 0.003 | 0.018 | 0.861 | 162.287 |
| rs111920933 | Gastric cancer | 8 | 66302667 | C/T | 0.021 | 0.003 | 0.163 | 0.987 | 216.143 |
| rs6415604 | Gastric cancer | 8 | 66736695 | C/T | 0.103 | -0.001 | 0.029 | 0.967 | 39.159 |
| rs13255885 | Gastric cancer | 8 | 66788754 | T/C | 0.516 | -0.003 | 0.018 | 0.849 | 417.248 |
| rs72666548 | Gastric cancer | 8 | 66680271 | T/C | 0.030 | -0.004 | 0.141 | 0.976 | 47.819 |
| rs11557049 | Gastric cancer | 8 | 66692011 | T/C | 0.061 | -0.006 | 0.094 | 0.952 | 56.901 |
| rs6995183 | Gastric cancer | 8 | 66653081 | C/T | 0.656 | -0.006 | 0.016 | 0.722 | 1279.282 |
| rs34863513 | Gastric cancer | 8 | 66306959 | G/A | 0.219 | -0.012 | 0.018 | 0.482 | 24.086 |
| rs11991790 | Gastric cancer | 8 | 66372747 | T/C | 0.631 | -0.014 | 0.016 | 0.365 | 169.738 |
| rs16932223 | Gastric cancer | 8 | 66470243 | G/A | 0.188 | -0.018 | 0.017 | 0.272 | 56.528 |
| rs35042734 | Gastric cancer | 8 | 66635118 | G/A | 0.050 | -0.019 | 0.088 | 0.827 | 82.686 |
| rs117457705 | Gastric cancer | 8 | 66289809 | A/G | 0.024 | -0.022 | 0.155 | 0.887 | 30.372 |
| rs62506046 | Gastric cancer | 8 | 66445177 | A/G | 0.093 | -0.023 | 0.077 | 0.768 | 41.355 |
| rs6472207 | Gastric cancer | 8 | 66474197 | G/A | 0.669 | -0.027 | 0.016 | 0.095 | 2109.998 |
| rs75003352 | Gastric cancer | 8 | 66892263 | T/C | 0.029 | -0.027 | 0.137 | 0.843 | 24.112 |
| rs13262427 | Gastric cancer | 8 | 66287974 | T/C | 0.107 | -0.035 | 0.074 | 0.636 | 22.641 |
| rs62507651 | Gastric cancer | 8 | 66712028 | A/G | 0.053 | -0.041 | 0.100 | 0.684 | 36.218 |
| rs118107026 | Gastric cancer | 8 | 66797673 | A/G | 0.034 | -0.050 | 0.125 | 0.691 | 78.805 |
| rs72662837 | Gastric cancer | 8 | 66311966 | T/C | 0.032 | -0.051 | 0.128 | 0.691 | 391.549 |
| rs117066002 | Gastric cancer | 8 | 66866098 | G/A | 0.046 | -0.053 | 0.108 | 0.622 | 30.055 |
| rs76347759 | Gastric cancer | 8 | 66891150 | G/A | 0.040 | -0.055 | 0.116 | 0.638 | 460.234 |
| rs62507636 | Gastric cancer | 8 | 66613267 | A/G | 0.038 | -0.056 | 0.116 | 0.628 | 22.463 |
| rs117775394 | Gastric cancer | 8 | 66679476 | A/G | 0.019 | -0.061 | 0.165 | 0.709 | 133.407 |
| rs73237256 | Gastric cancer | 8 | 66914816 | C/T | 0.057 | -0.065 | 0.035 | 0.065 | 41.229 |
| rs112672393 | Gastric cancer | 8 | 66652689 | G/A | 0.133 | -0.067 | 0.036 | 0.065 | 48.570 |
| rs62507573 | Gastric cancer | 8 | 66474880 | A/G | 0.038 | -0.068 | 0.116 | 0.557 | 21.814 |
| rs75599573 | Gastric cancer | 8 | 66772874 | A/G | 0.161 | -0.083 | 0.034 | 0.016 | 33.469 |
| rs142210893 | Gastric cancer | 8 | 66371986 | A/C | 0.026 | -0.096 | 0.141 | 0.495 | 26.957 |
| rs34535034 | Gastric cancer | 8 | 66601272 | T/C | 0.022 | -0.099 | 0.156 | 0.524 | 24.583 |
| rs62506587 | Gastric cancer | 8 | 66865952 | G/A | 0.033 | -0.106 | 0.125 | 0.399 | 299.706 |
| rs144154281 | Gastric cancer | 8 | 66522478 | A/G | 0.018 | -0.121 | 0.170 | 0.478 | 317.820 |
| rs17302765 | Gastric cancer | 8 | 66474459 | G/A | 0.060 | -0.122 | 0.095 | 0.197 | 243.148 |
| rs77821074 | Gastric cancer | 8 | 66372275 | G/A | 0.045 | -0.134 | 0.106 | 0.208 | 22.783 |
| rs113057267 | Gastric cancer | 8 | 66812506 | C/A | 0.070 | -0.260 | 0.108 | 0.017 | 57.183 |
| rs74662969 | Gastric cancer | 8 | 66546420 | T/C | 0.024 | -0.270 | 0.146 | 0.064 | 29.205 |
| rs75725452 | Gastric cancer | 8 | 66385725 | C/T | 0.023 | -0.297 | 0.147 | 0.044 | 20.582 |
| ECHDC1 |  |  |  |  |  |  |  |  |  |
| rs76148210 | Gastric cancer | 6 | 127893482 | G/T | 0.010 | 0.262 | 0.228 | 0.250 | 24.310 |
| rs62437084 | Gastric cancer | 6 | 127905364 | G/A | 0.020 | 0.222 | 0.163 | 0.174 | 22.668 |
| rs75560446 | Gastric cancer | 6 | 127753798 | C/T | 0.037 | 0.190 | 0.119 | 0.112 | 89.861 |
| rs72960548 | Gastric cancer | 6 | 127873865 | C/T | 0.058 | 0.178 | 0.096 | 0.064 | 54.639 |
| rs17648408 | Gastric cancer | 6 | 127653443 | T/C | 0.027 | 0.164 | 0.138 | 0.232 | 71.663 |
| rs72960556 | Gastric cancer | 6 | 127876436 | G/A | 0.046 | 0.162 | 0.109 | 0.139 | 39.986 |
| rs138907712 | Gastric cancer | 6 | 127592051 | T/C | 0.014 | 0.156 | 0.199 | 0.433 | 20.130 |
| rs143114633 | Gastric cancer | 6 | 127560233 | A/G | 0.018 | 0.150 | 0.178 | 0.399 | 62.939 |
| rs118002238 | Gastric cancer | 6 | 127695858 | T/C | 0.085 | 0.148 | 0.069 | 0.031 | 54.385 |
| rs76717644 | Gastric cancer | 6 | 127739329 | G/A | 0.061 | 0.146 | 0.096 | 0.127 | 41.070 |
| rs117325110 | Gastric cancer | 6 | 127547533 | C/T | 0.061 | 0.143 | 0.093 | 0.125 | 69.552 |
| rs145638120 | Gastric cancer | 6 | 127858847 | T/C | 0.037 | 0.121 | 0.118 | 0.307 | 77.129 |
| rs117623649 | Gastric cancer | 6 | 127760958 | A/G | 0.037 | 0.119 | 0.123 | 0.333 | 44.127 |
| rs13194028 | Gastric cancer | 6 | 127666068 | T/C | 0.306 | 0.108 | 0.045 | 0.016 | 569.555 |
| rs60059762 | Gastric cancer | 6 | 127900290 | G/A | 0.071 | 0.093 | 0.062 | 0.132 | 25.692 |
| rs77752188 | Gastric cancer | 6 | 127620879 | A/G | 0.019 | 0.092 | 0.169 | 0.585 | 83.946 |
| rs56393120 | Gastric cancer | 6 | 127682957 | T/C | 0.036 | 0.084 | 0.119 | 0.484 | 28.689 |
| rs10485028 | Gastric cancer | 6 | 127655444 | C/T | 0.037 | 0.081 | 0.119 | 0.498 | 41.818 |
| rs17816589 | Gastric cancer | 6 | 127956486 | A/G | 0.039 | 0.081 | 0.115 | 0.485 | 20.319 |
| rs148537879 | Gastric cancer | 6 | 127730082 | A/G | 0.017 | 0.080 | 0.180 | 0.655 | 73.718 |
| rs112759262 | Gastric cancer | 6 | 127938769 | A/G | 0.109 | 0.077 | 0.073 | 0.294 | 42.023 |
| rs148084229 | Gastric cancer | 6 | 127550318 | G/A | 0.036 | 0.064 | 0.124 | 0.606 | 47.886 |
| rs137994678 | Gastric cancer | 6 | 127819910 | T/C | 0.020 | 0.054 | 0.170 | 0.751 | 19.650 |
| rs1013640 | Gastric cancer | 6 | 127593564 | G/A | 0.967 | 0.053 | 0.125 | 0.670 | 104.062 |
| rs2068981 | Gastric cancer | 6 | 127697992 | C/A | 0.142 | 0.048 | 0.064 | 0.453 | 183.620 |
| rs117131035 | Gastric cancer | 6 | 127744342 | T/C | 0.034 | 0.048 | 0.122 | 0.697 | 49.623 |
| rs11968639 | Gastric cancer | 6 | 127663923 | A/C | 0.015 | 0.045 | 0.194 | 0.818 | 50.681 |
| rs6569504 | Gastric cancer | 6 | 127909720 | C/T | 0.626 | 0.042 | 0.047 | 0.370 | 67.550 |
| rs145176383 | Gastric cancer | 6 | 127551697 | A/G | 0.014 | 0.040 | 0.197 | 0.838 | 34.681 |
| rs11752269 | Gastric cancer | 6 | 127543618 | T/C | 0.153 | 0.037 | 0.063 | 0.552 | 49.375 |
| rs4410741 | Gastric cancer | 6 | 127550349 | C/T | 0.958 | 0.034 | 0.112 | 0.763 | 177.635 |
| rs11154410 | Gastric cancer | 6 | 127778845 | T/C | 0.367 | 0.029 | 0.046 | 0.534 | 688.567 |
| rs147516795 | Gastric cancer | 6 | 127569516 | C/T | 0.045 | 0.024 | 0.110 | 0.825 | 32.188 |
| rs4897217 | Gastric cancer | 6 | 127876145 | G/T | 0.165 | 0.014 | 0.019 | 0.452 | 99.884 |
| rs145237956 | Gastric cancer | 6 | 127908568 | T/C | 0.013 | 0.012 | 0.206 | 0.952 | 29.366 |
| rs595663 | Gastric cancer | 6 | 127828839 | C/T | 0.624 | 0.009 | 0.016 | 0.577 | 166.765 |
| rs11154421 | Gastric cancer | 6 | 127936007 | A/G | 0.489 | 0.003 | 0.018 | 0.868 | 111.586 |
| rs72961013 | Gastric cancer | 6 | 127529780 | A/G | 0.067 | 0.001 | 0.089 | 0.995 | 25.796 |
| rs145881926 | Gastric cancer | 6 | 127389101 | A/G | 0.037 | -0.001 | 0.118 | 0.994 | 47.726 |
| rs514064 | Gastric cancer | 6 | 127809055 | A/C | 0.043 | -0.009 | 0.110 | 0.938 | 155.763 |
| rs6922652 | Gastric cancer | 6 | 127847750 | T/C | 0.041 | -0.010 | 0.112 | 0.932 | 32.749 |
| rs7762790 | Gastric cancer | 6 | 127733917 | G/T | 0.969 | -0.011 | 0.130 | 0.936 | 88.018 |
| rs1913076 | Gastric cancer | 6 | 127853663 | T/C | 0.512 | -0.012 | 0.018 | 0.522 | 62.052 |
| rs41285264 | Gastric cancer | 6 | 127609558 | G/T | 0.057 | -0.015 | 0.042 | 0.715 | 34.840 |
| rs6913350 | Gastric cancer | 6 | 127570560 | T/C | 0.705 | -0.022 | 0.017 | 0.186 | 958.667 |
| rs77061892 | Gastric cancer | 6 | 127906211 | A/G | 0.062 | -0.028 | 0.030 | 0.347 | 24.377 |
| rs112657916 | Gastric cancer | 6 | 127837357 | G/A | 0.044 | -0.030 | 0.110 | 0.789 | 31.461 |
| rs55995239 | Gastric cancer | 6 | 127843090 | T/C | 0.043 | -0.034 | 0.112 | 0.760 | 59.015 |
| rs9491795 | Gastric cancer | 6 | 127787295 | T/G | 0.234 | -0.037 | 0.017 | 0.031 | 21.551 |
| rs111299996 | Gastric cancer | 6 | 127910882 | T/C | 0.041 | -0.039 | 0.116 | 0.740 | 29.012 |
| rs6928924 | Gastric cancer | 6 | 127555194 | T/C | 0.610 | -0.047 | 0.018 | 0.010 | 563.433 |
| rs982306 | Gastric cancer | 6 | 127777039 | C/T | 0.691 | -0.049 | 0.018 | 0.005 | 542.499 |
| rs7773484 | Gastric cancer | 6 | 127670779 | C/T | 0.756 | -0.052 | 0.019 | 0.005 | 983.374 |
| rs77548823 | Gastric cancer | 6 | 127959969 | T/C | 0.035 | -0.059 | 0.121 | 0.626 | 61.814 |
| rs9791192 | Gastric cancer | 6 | 127542953 | T/C | 0.203 | -0.063 | 0.055 | 0.251 | 251.888 |
| rs9491850 | Gastric cancer | 6 | 127952069 | A/G | 0.010 | -0.090 | 0.222 | 0.687 | 21.836 |
| rs118183176 | Gastric cancer | 6 | 127561931 | T/C | 0.041 | -0.113 | 0.113 | 0.316 | 25.946 |
| rs77024033 | Gastric cancer | 6 | 127642742 | C/T | 0.013 | -0.129 | 0.202 | 0.525 | 30.628 |
| rs12195271 | Gastric cancer | 6 | 127786453 | A/G | 0.039 | -0.141 | 0.116 | 0.226 | 46.035 |
| rs144657866 | Gastric cancer | 6 | 127921609 | C/T | 0.013 | -0.157 | 0.208 | 0.451 | 22.904 |
| rs79594909 | Gastric cancer | 6 | 127490688 | T/C | 0.016 | -0.209 | 0.188 | 0.267 | 22.902 |
| rs76376173 | Gastric cancer | 6 | 127561417 | C/T | 0.016 | -0.231 | 0.098 | 0.018 | 27.790 |
| SYNE2 |  |  |  |  |  |  |  |  |  |
| rs76359288 | Gastric cancer | 14 | 64277217 | A/C | 0.013 | 0.247 | 0.196 | 0.208 | 21.654 |
| rs139495004 | Gastric cancer | 14 | 64292420 | T/C | 0.080 | 0.119 | 0.083 | 0.154 | 30.355 |
| rs1255997 | Gastric cancer | 14 | 64693317 | C/T | 0.929 | 0.087 | 0.086 | 0.311 | 57.651 |
| rs142933020 | Gastric cancer | 14 | 64970884 | A/G | 0.035 | 0.085 | 0.123 | 0.492 | 26.694 |
| rs140809692 | Gastric cancer | 14 | 64512706 | A/G | 0.064 | 0.080 | 0.094 | 0.393 | 49.811 |
| rs77700773 | Gastric cancer | 14 | 64752671 | T/G | 0.055 | 0.078 | 0.100 | 0.435 | 69.310 |
| rs61985961 | Gastric cancer | 14 | 64875372 | T/C | 0.099 | 0.070 | 0.075 | 0.353 | 32.717 |
| rs117042313 | Gastric cancer | 14 | 64769891 | G/A | 0.028 | 0.069 | 0.134 | 0.607 | 28.421 |
| rs116949928 | Gastric cancer | 14 | 64453699 | T/C | 0.094 | 0.069 | 0.078 | 0.380 | 25.024 |
| rs113982525 | Gastric cancer | 14 | 64438470 | G/A | 0.021 | 0.067 | 0.158 | 0.673 | 23.300 |
| rs55767709 | Gastric cancer | 14 | 64794216 | C/T | 0.238 | 0.054 | 0.056 | 0.335 | 68.633 |
| rs12435284 | Gastric cancer | 14 | 64740520 | T/C | 0.070 | 0.053 | 0.060 | 0.375 | 19.728 |
| rs138465627 | Gastric cancer | 14 | 64101649 | T/C | 0.099 | 0.053 | 0.077 | 0.494 | 27.211 |
| rs111332176 | Gastric cancer | 14 | 64230339 | A/G | 0.041 | 0.042 | 0.042 | 0.314 | 162.822 |
| rs11158532 | Gastric cancer | 14 | 64603204 | G/A | 0.057 | 0.040 | 0.028 | 0.147 | 42.024 |
| rs1256112 | Gastric cancer | 14 | 64814311 | T/C | 0.574 | 0.040 | 0.016 | 0.016 | 395.496 |
| rs58126300 | Gastric cancer | 14 | 64704541 | A/G | 0.074 | 0.037 | 0.073 | 0.612 | 49.398 |
| rs61985962 | Gastric cancer | 14 | 64878290 | T/C | 0.203 | 0.035 | 0.023 | 0.137 | 30.922 |
| rs56317344 | Gastric cancer | 14 | 64739526 | T/C | 0.016 | 0.031 | 0.191 | 0.873 | 40.571 |
| rs1256051 | Gastric cancer | 14 | 64720338 | G/A | 0.211 | 0.029 | 0.017 | 0.096 | 67.910 |
| rs1760988 | Gastric cancer | 14 | 64696813 | G/A | 0.568 | 0.029 | 0.016 | 0.075 | 664.166 |
| rs11158509 | Gastric cancer | 14 | 64271996 | G/T | 0.548 | 0.023 | 0.016 | 0.154 | 68.171 |
| rs28575623 | Gastric cancer | 14 | 64106402 | T/C | 0.049 | 0.019 | 0.042 | 0.655 | 253.604 |
| rs4899124 | Gastric cancer | 14 | 64406101 | C/T | 0.254 | 0.017 | 0.022 | 0.441 | 26.459 |
| rs143394299 | Gastric cancer | 14 | 64714338 | G/A | 0.079 | 0.017 | 0.084 | 0.840 | 44.676 |
| rs12431522 | Gastric cancer | 14 | 64567091 | G/A | 0.243 | 0.014 | 0.017 | 0.406 | 88.548 |
| rs1256142 | Gastric cancer | 14 | 64910794 | A/G | 0.495 | 0.011 | 0.016 | 0.483 | 22.827 |
| rs138645167 | Gastric cancer | 14 | 64641126 | G/A | 0.033 | 0.011 | 0.126 | 0.931 | 26.812 |
| rs4899136 | Gastric cancer | 14 | 64888533 | T/C | 0.976 | 0.011 | 0.148 | 0.943 | 51.021 |
| rs76993093 | Gastric cancer | 14 | 64899516 | T/C | 0.226 | 0.010 | 0.019 | 0.612 | 28.219 |
| rs148114281 | Gastric cancer | 14 | 64626959 | T/C | 0.028 | 0.005 | 0.138 | 0.970 | 23.631 |
| rs78104306 | Gastric cancer | 14 | 64543330 | C/A | 0.241 | 0.005 | 0.023 | 0.832 | 25.417 |
| rs45512391 | Gastric cancer | 14 | 64988830 | A/C | 0.106 | 0.004 | 0.072 | 0.961 | 56.542 |
| rs60045886 | Gastric cancer | 14 | 64565235 | T/C | 0.075 | 0.003 | 0.032 | 0.929 | 57.149 |
| rs12589834 | Gastric cancer | 14 | 64771405 | G/A | 0.221 | 0.002 | 0.028 | 0.953 | 267.764 |
| rs61984458 | Gastric cancer | 14 | 64861479 | T/C | 0.026 | 0.001 | 0.139 | 0.995 | 34.649 |
| rs17101765 | Gastric cancer | 14 | 64759869 | C/T | 0.273 | -0.001 | 0.019 | 0.969 | 227.288 |
| rs2781381 | Gastric cancer | 14 | 64646048 | C/T | 0.240 | -0.002 | 0.020 | 0.935 | 192.992 |
| rs2256191 | Gastric cancer | 14 | 64656855 | C/T | 0.329 | -0.002 | 0.017 | 0.905 | 184.752 |
| rs35852840 | Gastric cancer | 14 | 64595763 | A/C | 0.054 | -0.004 | 0.098 | 0.970 | 58.052 |
| rs1266817 | Gastric cancer | 14 | 64055403 | C/T | 0.460 | -0.004 | 0.019 | 0.815 | 41.188 |
| rs12897659 | Gastric cancer | 14 | 64241317 | G/A | 0.067 | -0.007 | 0.034 | 0.830 | 71.080 |
| rs12894963 | Gastric cancer | 14 | 64362363 | G/A | 0.077 | -0.011 | 0.027 | 0.688 | 61.877 |
| rs117117282 | Gastric cancer | 14 | 64047263 | G/T | 0.015 | -0.017 | 0.054 | 0.758 | 58.512 |
| rs7149410 | Gastric cancer | 14 | 64121392 | A/G | 0.270 | -0.020 | 0.018 | 0.264 | 46.474 |
| rs12586503 | Gastric cancer | 14 | 64498925 | C/T | 0.918 | -0.033 | 0.025 | 0.185 | 64.467 |
| rs73280659 | Gastric cancer | 14 | 64071886 | C/A | 0.259 | -0.049 | 0.068 | 0.469 | 23.088 |
| rs12889934 | Gastric cancer | 14 | 64213679 | A/C | 0.047 | -0.060 | 0.040 | 0.133 | 22.886 |
| rs145160226 | Gastric cancer | 14 | 64597400 | T/C | 0.080 | -0.071 | 0.084 | 0.393 | 25.678 |
| rs10140284 | Gastric cancer | 14 | 64882968 | C/T | 0.808 | -0.079 | 0.069 | 0.253 | 37.878 |
| rs113370836 | Gastric cancer | 14 | 64741163 | A/G | 0.023 | -0.085 | 0.151 | 0.575 | 27.318 |
| rs113410851 | Gastric cancer | 14 | 64878407 | T/C | 0.038 | -0.101 | 0.119 | 0.399 | 24.312 |
| rs17751301 | Gastric cancer | 14 | 64469828 | T/C | 0.068 | -0.121 | 0.088 | 0.171 | 89.467 |
| rs12884767 | Gastric cancer | 14 | 64839007 | C/A | 0.050 | -0.154 | 0.180 | 0.392 | 90.280 |
| rs142119488 | Gastric cancer | 14 | 64804831 | C/T | 0.045 | -0.159 | 0.197 | 0.420 | 30.100 |
| rs79987469 | Gastric cancer | 14 | 64646647 | T/C | 0.027 | -0.189 | 0.139 | 0.176 | 31.773 |
| PLEKHB2 |  |  |  |  |  |  |  |  |  |
| rs80304145 | Gastric cancer | 2 | 131915357 | A/G | 0.071 | 0.299 | 0.088 | 0.001 | 21.419 |
| rs145000671 | Gastric cancer | 2 | 132087331 | G/A | 0.059 | 0.249 | 0.100 | 0.012 | 30.280 |
| rs192213260 | Gastric cancer | 2 | 132133526 | T/C | 0.024 | 0.152 | 0.147 | 0.302 | 70.996 |
| rs74464080 | Gastric cancer | 2 | 131801416 | G/A | 0.025 | 0.122 | 0.141 | 0.385 | 66.914 |
| rs12475643 | Gastric cancer | 2 | 131659345 | T/C | 0.116 | 0.064 | 0.070 | 0.361 | 21.911 |
| rs111727878 | Gastric cancer | 2 | 131865037 | A/G | 0.040 | 0.059 | 0.114 | 0.603 | 19.633 |
| rs35904592 | Gastric cancer | 2 | 131887984 | G/A | 0.030 | 0.035 | 0.131 | 0.790 | 42.428 |
| rs72863203 | Gastric cancer | 2 | 131768080 | A/G | 0.092 | 0.027 | 0.024 | 0.260 | 59.716 |
| rs7419337 | Gastric cancer | 2 | 131777291 | T/C | 0.957 | 0.026 | 0.027 | 0.347 | 22.524 |
| rs62178928 | Gastric cancer | 2 | 131738119 | A/G | 0.815 | 0.021 | 0.025 | 0.414 | 64.797 |
| rs7340467 | Gastric cancer | 2 | 131693828 | A/G | 0.762 | 0.019 | 0.023 | 0.411 | 50.438 |
| rs12466662 | Gastric cancer | 2 | 131774186 | C/T | 0.233 | 0.007 | 0.019 | 0.707 | 41.120 |
| rs12998401 | Gastric cancer | 2 | 131790799 | A/G | 0.807 | 0.001 | 0.020 | 0.941 | 45.852 |
| rs10165540 | Gastric cancer | 2 | 131792098 | A/G | 0.080 | 0.000 | 0.026 | 0.995 | 194.016 |
| rs10185789 | Gastric cancer | 2 | 131755154 | T/C | 0.241 | -0.010 | 0.017 | 0.557 | 73.386 |
| rs918958 | Gastric cancer | 2 | 131869747 | A/G | 0.824 | -0.011 | 0.027 | 0.695 | 62.254 |
| rs7600253 | Gastric cancer | 2 | 131983633 | A/G | 0.652 | -0.021 | 0.020 | 0.290 | 100.359 |
| rs6727332 | Gastric cancer | 2 | 132137354 | T/G | 0.812 | -0.022 | 0.021 | 0.295 | 160.809 |
| rs55637978 | Gastric cancer | 2 | 132027891 | G/A | 0.854 | -0.027 | 0.023 | 0.235 | 267.580 |
| rs2272355 | Gastric cancer | 2 | 132164854 | C/T | 0.445 | -0.027 | 0.018 | 0.131 | 19.935 |
| rs272105 | Gastric cancer | 2 | 131604057 | C/A | 0.110 | -0.029 | 0.022 | 0.179 | 33.358 |
| rs116803649 | Gastric cancer | 2 | 131691239 | A/G | 0.024 | -0.036 | 0.154 | 0.815 | 32.084 |
| rs192025422 | Gastric cancer | 2 | 132078921 | T/C | 0.032 | -0.043 | 0.129 | 0.742 | 102.089 |
| rs143137049 | Gastric cancer | 2 | 131948293 | G/A | 0.029 | -0.069 | 0.137 | 0.613 | 95.119 |
| SMC6 |  |  |  |  |  |  |  |  |  |
| rs17380757 | Gastric cancer | 2 | 17818539 | A/G | 0.010 | 0.300 | 0.229 | 0.190 | 22.432 |
| rs144049374 | Gastric cancer | 2 | 17597779 | G/A | 0.015 | 0.290 | 0.192 | 0.130 | 183.689 |
| rs55809808 | Gastric cancer | 2 | 17549230 | C/T | 0.032 | 0.273 | 0.129 | 0.035 | 90.170 |
| rs181068758 | Gastric cancer | 2 | 18167947 | C/T | 0.019 | 0.160 | 0.171 | 0.348 | 44.032 |
| rs73921059 | Gastric cancer | 2 | 17914623 | G/A | 0.029 | 0.132 | 0.134 | 0.323 | 100.752 |
| rs78218998 | Gastric cancer | 2 | 18126876 | T/C | 0.056 | 0.126 | 0.098 | 0.196 | 33.365 |
| rs55909808 | Gastric cancer | 2 | 18117101 | C/T | 0.070 | 0.124 | 0.088 | 0.156 | 157.462 |
| rs17380100 | Gastric cancer | 2 | 17681504 | C/T | 0.067 | 0.122 | 0.090 | 0.176 | 130.901 |
| rs11688560 | Gastric cancer | 2 | 17617770 | C/A | 0.027 | 0.116 | 0.140 | 0.409 | 156.861 |
| rs76436637 | Gastric cancer | 2 | 17649370 | A/G | 0.048 | 0.116 | 0.106 | 0.276 | 247.581 |
| rs146484001 | Gastric cancer | 2 | 17728454 | A/G | 0.018 | 0.111 | 0.173 | 0.520 | 41.869 |
| rs4832517 | Gastric cancer | 2 | 18094112 | T/C | 0.022 | 0.092 | 0.042 | 0.026 | 50.396 |
| rs13010719 | Gastric cancer | 2 | 18046551 | T/C | 0.021 | 0.090 | 0.155 | 0.560 | 22.103 |
| rs114348667 | Gastric cancer | 2 | 17623685 | A/G | 0.020 | 0.089 | 0.159 | 0.577 | 34.120 |
| rs189735123 | Gastric cancer | 2 | 17912883 | G/A | 0.011 | 0.086 | 0.225 | 0.703 | 34.551 |
| rs2710661 | Gastric cancer | 2 | 17862966 | A/G | 0.259 | 0.084 | 0.040 | 0.036 | 34.000 |
| rs144107306 | Gastric cancer | 2 | 18083915 | G/A | 0.019 | 0.078 | 0.169 | 0.643 | 47.300 |
| rs62130899 | Gastric cancer | 2 | 17980834 | A/G | 0.087 | 0.078 | 0.080 | 0.334 | 181.621 |
| rs58126519 | Gastric cancer | 2 | 18090114 | A/G | 0.019 | 0.066 | 0.086 | 0.441 | 21.807 |
| rs74728324 | Gastric cancer | 2 | 17825692 | T/C | 0.008 | 0.065 | 0.254 | 0.799 | 72.831 |
| rs55676851 | Gastric cancer | 2 | 18124734 | T/C | 0.194 | 0.048 | 0.017 | 0.004 | 21.582 |
| rs6738866 | Gastric cancer | 2 | 17786257 | C/T | 0.937 | 0.047 | 0.092 | 0.609 | 1092.528 |
| rs72770434 | Gastric cancer | 2 | 18039763 | C/T | 0.023 | 0.047 | 0.151 | 0.754 | 34.173 |
| rs12467070 | Gastric cancer | 2 | 18037272 | G/A | 0.304 | 0.045 | 0.016 | 0.006 | 110.635 |
| rs6718282 | Gastric cancer | 2 | 18039651 | A/G | 0.161 | 0.045 | 0.017 | 0.009 | 352.549 |
| rs17315862 | Gastric cancer | 2 | 17993836 | C/T | 0.039 | 0.045 | 0.116 | 0.699 | 320.019 |
| rs67337720 | Gastric cancer | 2 | 17597414 | T/G | 0.038 | 0.045 | 0.119 | 0.706 | 65.046 |
| rs112579150 | Gastric cancer | 2 | 18164085 | C/T | 0.027 | 0.042 | 0.033 | 0.212 | 39.022 |
| rs62130915 | Gastric cancer | 2 | 17995017 | A/G | 0.042 | 0.041 | 0.111 | 0.710 | 36.902 |
| rs430583 | Gastric cancer | 2 | 17663441 | T/C | 0.463 | 0.028 | 0.023 | 0.225 | 1745.357 |
| rs2970936 | Gastric cancer | 2 | 17559802 | A/G | 0.341 | 0.022 | 0.034 | 0.521 | 32.416 |
| rs2710688 | Gastric cancer | 2 | 17763822 | C/T | 0.691 | 0.019 | 0.023 | 0.392 | 119.496 |
| rs17388422 | Gastric cancer | 2 | 18125150 | C/A | 0.110 | 0.013 | 0.042 | 0.758 | 106.261 |
| rs2061607 | Gastric cancer | 2 | 18076052 | G/A | 0.898 | 0.012 | 0.040 | 0.771 | 29.266 |
| rs72610788 | Gastric cancer | 2 | 17854118 | G/T | 0.245 | 0.010 | 0.019 | 0.619 | 1310.529 |
| rs10186459 | Gastric cancer | 2 | 18148175 | A/G | 0.474 | 0.008 | 0.017 | 0.633 | 80.251 |
| rs17379899 | Gastric cancer | 2 | 17616302 | T/C | 0.212 | 0.008 | 0.038 | 0.844 | 636.582 |
| rs2732938 | Gastric cancer | 2 | 18149846 | T/C | 0.079 | 0.008 | 0.022 | 0.738 | 25.285 |
| rs12470501 | Gastric cancer | 2 | 18185590 | C/T | 0.286 | 0.007 | 0.018 | 0.694 | 44.044 |
| rs7556886 | Gastric cancer | 2 | 17908130 | C/T | 0.631 | 0.002 | 0.021 | 0.915 | 3195.467 |
| rs113801851 | Gastric cancer | 2 | 17803601 | T/C | 0.039 | 0.002 | 0.117 | 0.987 | 425.031 |
| rs78421883 | Gastric cancer | 2 | 18036708 | T/C | 0.085 | 0.002 | 0.052 | 0.974 | 273.085 |
| rs35940501 | Gastric cancer | 2 | 17596690 | T/C | 0.084 | 0.001 | 0.058 | 0.986 | 181.236 |
| rs6727841 | Gastric cancer | 2 | 18013986 | C/T | 0.659 | -0.003 | 0.022 | 0.882 | 845.330 |
| rs7556983 | Gastric cancer | 2 | 17955255 | A/G | 0.099 | -0.004 | 0.027 | 0.883 | 77.326 |
| rs114353721 | Gastric cancer | 2 | 18184750 | A/G | 0.125 | -0.004 | 0.019 | 0.824 | 79.673 |
| rs365337 | Gastric cancer | 2 | 17621947 | T/C | 0.061 | -0.005 | 0.042 | 0.915 | 959.568 |
| rs2957565 | Gastric cancer | 2 | 18211663 | C/T | 0.194 | -0.005 | 0.019 | 0.786 | 78.172 |
| rs17314101 | Gastric cancer | 2 | 17557275 | T/G | 0.236 | -0.006 | 0.053 | 0.909 | 172.580 |
| rs1033297 | Gastric cancer | 2 | 17768612 | A/G | 0.263 | -0.006 | 0.061 | 0.917 | 2112.885 |
| rs386921 | Gastric cancer | 2 | 17623246 | T/C | 0.294 | -0.007 | 0.049 | 0.885 | 340.395 |
| rs12465254 | Gastric cancer | 2 | 18032792 | T/G | 0.412 | -0.019 | 0.025 | 0.456 | 462.563 |
| rs2114352 | Gastric cancer | 2 | 17822675 | G/A | 0.289 | -0.019 | 0.028 | 0.493 | 1393.457 |
| rs300151 | Gastric cancer | 2 | 17986997 | T/C | 0.604 | -0.020 | 0.026 | 0.444 | 250.498 |
| rs57385262 | Gastric cancer | 2 | 18118652 | G/T | 0.261 | -0.026 | 0.032 | 0.412 | 48.715 |
| rs6723332 | Gastric cancer | 2 | 17561906 | T/C | 0.515 | -0.026 | 0.022 | 0.229 | 1134.391 |
| rs10177831 | Gastric cancer | 2 | 18080382 | A/G | 0.503 | -0.028 | 0.016 | 0.080 | 44.005 |
| rs13003075 | Gastric cancer | 2 | 17626606 | A/C | 0.371 | -0.032 | 0.056 | 0.571 | 167.187 |
| rs138859246 | Gastric cancer | 2 | 18061441 | G/A | 0.008 | -0.032 | 0.250 | 0.899 | 23.120 |
| rs143570534 | Gastric cancer | 2 | 18068630 | A/G | 0.138 | -0.032 | 0.017 | 0.060 | 108.040 |
| rs114914021 | Gastric cancer | 2 | 17899291 | T/C | 0.026 | -0.039 | 0.142 | 0.785 | 48.450 |
| rs111478506 | Gastric cancer | 2 | 18200574 | A/G | 0.049 | -0.040 | 0.106 | 0.704 | 21.453 |
| rs1471342 | Gastric cancer | 2 | 18122198 | T/C | 0.367 | -0.041 | 0.016 | 0.013 | 23.137 |
| rs76858146 | Gastric cancer | 2 | 18038652 | A/G | 0.034 | -0.041 | 0.038 | 0.280 | 240.463 |
| rs72782551 | Gastric cancer | 2 | 18219932 | T/C | 0.054 | -0.042 | 0.100 | 0.676 | 25.913 |
| rs74403704 | Gastric cancer | 2 | 17990531 | C/T | 0.144 | -0.042 | 0.064 | 0.509 | 27.172 |
| rs12614549 | Gastric cancer | 2 | 18046715 | T/C | 0.256 | -0.046 | 0.018 | 0.009 | 83.203 |
| rs35890406 | Gastric cancer | 2 | 17743103 | C/T | 0.065 | -0.047 | 0.068 | 0.494 | 213.053 |
| rs146177537 | Gastric cancer | 2 | 18081917 | T/C | 0.047 | -0.050 | 0.105 | 0.638 | 55.223 |
| rs142695956 | Gastric cancer | 2 | 18168339 | G/A | 0.083 | -0.055 | 0.030 | 0.065 | 120.378 |
| rs75115837 | Gastric cancer | 2 | 17872205 | T/C | 0.038 | -0.056 | 0.117 | 0.633 | 224.295 |
| rs62130874 | Gastric cancer | 2 | 17866767 | T/C | 0.036 | -0.061 | 0.123 | 0.617 | 59.796 |
| rs148008218 | Gastric cancer | 2 | 17686320 | G/A | 0.012 | -0.063 | 0.203 | 0.757 | 185.436 |
| rs114350872 | Gastric cancer | 2 | 18091890 | C/A | 0.019 | -0.064 | 0.173 | 0.713 | 25.701 |
| rs78821391 | Gastric cancer | 2 | 17787453 | T/C | 0.025 | -0.064 | 0.144 | 0.656 | 289.634 |
| rs111964567 | Gastric cancer | 2 | 17981072 | T/G | 0.018 | -0.069 | 0.160 | 0.668 | 169.768 |
| rs73919098 | Gastric cancer | 2 | 17768661 | T/C | 0.094 | -0.073 | 0.103 | 0.480 | 28.732 |
| rs6740104 | Gastric cancer | 2 | 17789728 | A/G | 0.014 | -0.073 | 0.186 | 0.695 | 60.584 |
| rs13413088 | Gastric cancer | 2 | 17992503 | C/T | 0.017 | -0.074 | 0.175 | 0.671 | 85.689 |
| rs74822267 | Gastric cancer | 2 | 18207635 | C/T | 0.015 | -0.077 | 0.181 | 0.672 | 57.372 |
| rs78144901 | Gastric cancer | 2 | 17930118 | T/C | 0.025 | -0.078 | 0.145 | 0.593 | 275.158 |
| rs4832504 | Gastric cancer | 2 | 18034741 | C/A | 0.943 | -0.085 | 0.116 | 0.466 | 52.966 |
| rs4832492 | Gastric cancer | 2 | 17557444 | A/G | 0.172 | -0.099 | 0.060 | 0.102 | 183.079 |
| rs11096483 | Gastric cancer | 2 | 17970917 | C/T | 0.030 | -0.105 | 0.133 | 0.431 | 63.298 |
| rs145773379 | Gastric cancer | 2 | 18142576 | T/C | 0.016 | -0.163 | 0.182 | 0.372 | 23.371 |
| rs150293354 | Gastric cancer | 2 | 17957798 | G/A | 0.019 | -0.178 | 0.164 | 0.279 | 48.514 |
| rs73918952 | Gastric cancer | 2 | 18015731 | T/G | 0.078 | -0.178 | 0.083 | 0.032 | 77.287 |
| rs187059752 | Gastric cancer | 2 | 17662458 | T/C | 0.014 | -0.186 | 0.194 | 0.338 | 164.853 |
| rs149467486 | Gastric cancer | 2 | 17898735 | G/T | 0.042 | -0.197 | 0.111 | 0.076 | 31.105 |
| rs13432060 | Gastric cancer | 2 | 17572609 | G/A | 0.047 | -0.202 | 0.106 | 0.056 | 20.406 |
| rs138906197 | Gastric cancer | 2 | 18089629 | C/T | 0.082 | -0.204 | 0.081 | 0.012 | 41.899 |
| rs138707329 | Gastric cancer | 2 | 17740590 | A/G | 0.039 | -0.212 | 0.115 | 0.066 | 24.666 |
| rs111384209 | Gastric cancer | 2 | 17942304 | A/C | 0.039 | -0.217 | 0.114 | 0.057 | 150.654 |
| rs76956316 | Gastric cancer | 2 | 17597668 | G/A | 0.041 | -0.222 | 0.113 | 0.050 | 30.487 |
| rs143563114 | Gastric cancer | 2 | 17990536 | T/C | 0.022 | -0.281 | 0.151 | 0.062 | 101.956 |
| rs115580380 | Gastric cancer | 2 | 17672354 | C/T | 0.022 | -0.347 | 0.153 | 0.023 | 151.824 |
| rs141380821 | Gastric cancer | 2 | 17845025 | C/T | 0.012 | -0.451 | 0.203 | 0.027 | 249.984 |
| STAM2 |  |  |  |  |  |  |  |  |  |
| rs12992391 | Gastric cancer | 2 | 153220086 | C/T | 0.074 | 0.256 | 0.102 | 0.013 | 52.402 |
| rs35641787 | Gastric cancer | 2 | 153007710 | C/T | 0.082 | 0.247 | 0.100 | 0.013 | 62.968 |
| rs184200509 | Gastric cancer | 2 | 153102130 | G/A | 0.014 | 0.114 | 0.194 | 0.557 | 58.373 |
| rs144544027 | Gastric cancer | 2 | 152766032 | T/G | 0.016 | 0.105 | 0.180 | 0.561 | 41.514 |
| rs74936354 | Gastric cancer | 2 | 152980018 | A/C | 0.051 | 0.086 | 0.101 | 0.396 | 73.137 |
| rs113109771 | Gastric cancer | 2 | 153269604 | G/A | 0.029 | 0.079 | 0.132 | 0.549 | 50.647 |
| rs115190500 | Gastric cancer | 2 | 152745209 | A/G | 0.017 | 0.069 | 0.176 | 0.695 | 23.307 |
| rs116826288 | Gastric cancer | 2 | 152850929 | A/G | 0.047 | 0.068 | 0.066 | 0.300 | 34.760 |
| rs148507338 | Gastric cancer | 2 | 152897101 | A/G | 0.020 | 0.066 | 0.161 | 0.683 | 91.657 |
| rs10181200 | Gastric cancer | 2 | 152754955 | T/C | 0.051 | 0.061 | 0.101 | 0.546 | 26.609 |
| rs59000518 | Gastric cancer | 2 | 152733542 | A/C | 0.018 | 0.055 | 0.219 | 0.801 | 29.792 |
| rs34584371 | Gastric cancer | 2 | 153037808 | A/G | 0.113 | 0.053 | 0.022 | 0.016 | 163.079 |
| rs10202294 | Gastric cancer | 2 | 152970576 | C/T | 0.025 | 0.052 | 0.051 | 0.303 | 39.569 |
| rs12618892 | Gastric cancer | 2 | 153222924 | T/C | 0.474 | 0.042 | 0.028 | 0.135 | 230.428 |
| rs78254888 | Gastric cancer | 2 | 153252278 | T/C | 0.095 | 0.042 | 0.023 | 0.068 | 49.282 |
| rs139447012 | Gastric cancer | 2 | 152868182 | T/C | 0.040 | 0.038 | 0.113 | 0.735 | 41.269 |
| rs111294304 | Gastric cancer | 2 | 152738523 | C/T | 0.045 | 0.031 | 0.111 | 0.777 | 62.620 |
| rs115824190 | Gastric cancer | 2 | 153099065 | A/G | 0.014 | 0.031 | 0.192 | 0.870 | 54.221 |
| rs12693293 | Gastric cancer | 2 | 153118734 | T/C | 0.402 | 0.017 | 0.021 | 0.425 | 498.941 |
| rs115054729 | Gastric cancer | 2 | 153292612 | T/C | 0.005 | 0.017 | 0.589 | 0.978 | 37.091 |
| rs7369093 | Gastric cancer | 2 | 153016513 | G/A | 0.465 | 0.011 | 0.021 | 0.618 | 680.895 |
| rs147160239 | Gastric cancer | 2 | 152955856 | A/G | 0.023 | 0.009 | 0.150 | 0.955 | 39.409 |
| rs6433952 | Gastric cancer | 2 | 153144375 | C/T | 0.502 | 0.008 | 0.022 | 0.733 | 34.654 |
| rs73967855 | Gastric cancer | 2 | 153248999 | A/G | 0.125 | 0.008 | 0.022 | 0.726 | 150.463 |
| rs2345897 | Gastric cancer | 2 | 153097659 | G/A | 0.861 | 0.006 | 0.034 | 0.859 | 740.874 |
| rs72868013 | Gastric cancer | 2 | 152708356 | T/C | 0.076 | 0.005 | 0.084 | 0.955 | 92.545 |
| rs12477734 | Gastric cancer | 2 | 152873993 | A/G | 0.293 | 0.001 | 0.017 | 0.951 | 222.194 |
| rs11682378 | Gastric cancer | 2 | 152864170 | C/T | 0.173 | -0.001 | 0.028 | 0.968 | 405.296 |
| rs10803937 | Gastric cancer | 2 | 152931638 | T/C | 0.555 | -0.001 | 0.022 | 0.949 | 41.890 |
| rs62175108 | Gastric cancer | 2 | 152915711 | T/C | 0.019 | -0.003 | 0.103 | 0.975 | 33.131 |
| rs3768655 | Gastric cancer | 2 | 152714023 | G/T | 0.189 | -0.006 | 0.017 | 0.696 | 39.286 |
| rs72860422 | Gastric cancer | 2 | 153127809 | G/A | 0.035 | -0.007 | 0.120 | 0.952 | 47.213 |
| rs11893938 | Gastric cancer | 2 | 152836455 | G/A | 0.206 | -0.012 | 0.016 | 0.453 | 110.421 |
| rs13383195 | Gastric cancer | 2 | 152760024 | C/T | 0.659 | -0.015 | 0.017 | 0.385 | 145.237 |
| rs58219183 | Gastric cancer | 2 | 153232781 | C/T | 0.125 | -0.018 | 0.026 | 0.504 | 433.010 |
| rs6756645 | Gastric cancer | 2 | 152762720 | G/T | 0.195 | -0.022 | 0.024 | 0.372 | 258.807 |
| rs72859833 | Gastric cancer | 2 | 152965141 | C/T | 0.136 | -0.023 | 0.036 | 0.525 | 783.668 |
| rs116304885 | Gastric cancer | 2 | 152902751 | A/G | 0.014 | -0.026 | 0.190 | 0.893 | 27.774 |
| rs78528713 | Gastric cancer | 2 | 153196389 | G/A | 0.037 | -0.026 | 0.117 | 0.823 | 86.689 |
| rs148368444 | Gastric cancer | 2 | 153063657 | T/C | 0.016 | -0.028 | 0.181 | 0.877 | 40.020 |
| rs10930853 | Gastric cancer | 2 | 152802817 | T/C | 0.911 | -0.041 | 0.021 | 0.058 | 51.177 |
| rs138103745 | Gastric cancer | 2 | 153076631 | T/C | 0.038 | -0.054 | 0.117 | 0.645 | 80.165 |
| rs62177368 | Gastric cancer | 2 | 153100688 | C/T | 0.028 | -0.055 | 0.137 | 0.688 | 39.461 |
| rs183381115 | Gastric cancer | 2 | 153053298 | A/G | 0.033 | -0.056 | 0.128 | 0.660 | 112.840 |
| rs148835268 | Gastric cancer | 2 | 152938128 | T/C | 0.037 | -0.059 | 0.090 | 0.514 | 226.331 |
| rs62179558 | Gastric cancer | 2 | 153218548 | G/A | 0.043 | -0.062 | 0.111 | 0.576 | 23.127 |
| rs186444939 | Gastric cancer | 2 | 152900957 | T/C | 0.054 | -0.081 | 0.100 | 0.419 | 51.057 |
| rs112129288 | Gastric cancer | 2 | 152716821 | T/C | 0.052 | -0.095 | 0.101 | 0.347 | 37.504 |
| rs148051888 | Gastric cancer | 2 | 152995545 | T/C | 0.018 | -0.118 | 0.172 | 0.493 | 46.687 |
| rs141931778 | Gastric cancer | 2 | 152909222 | A/G | 0.012 | -0.144 | 0.218 | 0.510 | 44.119 |
| rs114549473 | Gastric cancer | 2 | 152802571 | T/G | 0.019 | -0.152 | 0.171 | 0.375 | 38.623 |
| rs150487920 | Gastric cancer | 2 | 152907580 | T/C | 0.013 | -0.155 | 0.202 | 0.443 | 23.746 |
| rs75239367 | Gastric cancer | 2 | 153282016 | G/T | 0.022 | -0.159 | 0.154 | 0.301 | 41.011 |
| rs147625363 | Gastric cancer | 2 | 152733665 | A/G | 0.025 | -0.160 | 0.189 | 0.397 | 66.298 |
| rs140453553 | Gastric cancer | 2 | 152815505 | A/G | 0.021 | -0.178 | 0.164 | 0.278 | 31.548 |
| rs114468449 | Gastric cancer | 2 | 153095662 | T/C | 0.016 | -0.182 | 0.127 | 0.154 | 44.814 |
| TMEM106B |  |  |  |  |  |  |  |  |  |
| rs62448706 | Gastric cancer | 7 | 12321602 | A/C | 0.054 | 0.448 | 0.128 | 0.000 | 33.871 |
| rs62449483 | Gastric cancer | 7 | 12510742 | T/C | 0.055 | 0.284 | 0.101 | 0.005 | 30.945 |
| rs11773624 | Gastric cancer | 7 | 12023864 | A/G | 0.074 | 0.224 | 0.085 | 0.009 | 34.216 |
| rs62433256 | Gastric cancer | 7 | 12178544 | A/C | 0.057 | 0.216 | 0.098 | 0.027 | 39.655 |
| rs56242606 | Gastric cancer | 7 | 12421909 | C/T | 0.077 | 0.061 | 0.084 | 0.466 | 27.901 |
| rs76353014 | Gastric cancer | 7 | 12445066 | G/A | 0.038 | 0.060 | 0.117 | 0.608 | 36.906 |
| rs1440035 | Gastric cancer | 7 | 12386420 | C/T | 0.021 | 0.059 | 0.155 | 0.706 | 26.433 |
| rs117045293 | Gastric cancer | 7 | 12577134 | A/C | 0.040 | 0.046 | 0.115 | 0.690 | 20.079 |
| rs12699337 | Gastric cancer | 7 | 12283464 | C/T | 0.078 | 0.039 | 0.084 | 0.645 | 110.788 |
| rs138610878 | Gastric cancer | 7 | 12120991 | A/G | 0.017 | 0.020 | 0.178 | 0.911 | 39.675 |
| rs10258615 | Gastric cancer | 7 | 12243680 | T/C | 0.183 | 0.013 | 0.024 | 0.602 | 48.722 |
| rs62433210 | Gastric cancer | 7 | 12077889 | G/A | 0.144 | 0.010 | 0.021 | 0.629 | 38.456 |
| rs7810945 | Gastric cancer | 7 | 12197922 | G/A | 0.472 | 0.008 | 0.019 | 0.674 | 196.155 |
| rs1861279 | Gastric cancer | 7 | 12169531 | G/A | 0.485 | 0.002 | 0.016 | 0.884 | 33.914 |
| rs4291161 | Gastric cancer | 7 | 12568567 | A/C | 0.650 | -0.003 | 0.016 | 0.832 | 43.561 |
| rs12536822 | Gastric cancer | 7 | 12196391 | G/A | 0.255 | -0.004 | 0.021 | 0.839 | 111.703 |
| rs12699363 | Gastric cancer | 7 | 12387387 | A/G | 0.074 | -0.008 | 0.087 | 0.924 | 40.623 |
| rs6460915 | Gastric cancer | 7 | 12330255 | A/G | 0.591 | -0.009 | 0.016 | 0.556 | 64.911 |
| rs1595804 | Gastric cancer | 7 | 12487380 | G/A | 0.564 | -0.012 | 0.016 | 0.444 | 59.720 |
| rs12699392 | Gastric cancer | 7 | 12561888 | C/T | 0.158 | -0.013 | 0.061 | 0.828 | 27.688 |
| rs73056720 | Gastric cancer | 7 | 12508304 | A/G | 0.184 | -0.015 | 0.017 | 0.404 | 23.810 |
| rs10950386 | Gastric cancer | 7 | 12161751 | T/C | 0.122 | -0.016 | 0.029 | 0.587 | 42.041 |
| rs6960010 | Gastric cancer | 7 | 12129179 | T/C | 0.287 | -0.017 | 0.022 | 0.450 | 37.956 |
| rs2908742 | Gastric cancer | 7 | 12016050 | T/C | 0.746 | -0.025 | 0.038 | 0.508 | 22.673 |
| rs182393305 | Gastric cancer | 7 | 12291458 | A/C | 0.020 | -0.034 | 0.191 | 0.859 | 35.754 |
| rs117108792 | Gastric cancer | 7 | 12107772 | A/G | 0.011 | -0.051 | 0.219 | 0.818 | 19.859 |
| rs145475937 | Gastric cancer | 7 | 12272001 | G/A | 0.021 | -0.170 | 0.157 | 0.279 | 19.657 |
| rs78890701 | Gastric cancer | 7 | 12120380 | T/C | 0.015 | -0.271 | 0.186 | 0.145 | 34.865 |
| NDUFA4 |  |  |  |  |  |  |  |  |  |
| rs1629587 | Gastric cancer | 7 | 11065278 | T/C | 0.518 | 0.061 | 0.044 | 0.173 | 27.846 |
| rs55965831 | Gastric cancer | 7 | 10979881 | C/T | 0.036 | 0.041 | 0.121 | 0.736 | 22.907 |
| rs10278471 | Gastric cancer | 7 | 10907315 | A/G | 0.660 | 0.028 | 0.020 | 0.155 | 33.316 |
| rs7357204 | Gastric cancer | 7 | 10928266 | G/T | 0.528 | 0.021 | 0.020 | 0.297 | 38.069 |
| rs218970 | Gastric cancer | 7 | 10986944 | G/A | 0.216 | 0.011 | 0.022 | 0.605 | 36.624 |
| rs6953367 | Gastric cancer | 7 | 10957253 | C/T | 0.549 | -0.010 | 0.016 | 0.543 | 45.673 |
| rs7811275 | Gastric cancer | 7 | 11153833 | T/C | 0.036 | -0.023 | 0.121 | 0.846 | 30.610 |
| rs7357146 | Gastric cancer | 7 | 11012852 | T/C | 0.100 | -0.072 | 0.075 | 0.335 | 26.181 |
| MCTP2 |  |  |  |  |  |  |  |  |  |
| rs60113814 | Gastric cancer | 15 | 94624488 | A/G | 0.135 | 0.300 | 0.117 | 0.010 | 21.581 |
| rs111581307 | Gastric cancer | 15 | 94749297 | T/C | 0.027 | 0.258 | 0.147 | 0.078 | 35.605 |
| rs145553671 | Gastric cancer | 15 | 94671251 | T/G | 0.021 | 0.257 | 0.157 | 0.101 | 29.273 |
| rs11858500 | Gastric cancer | 15 | 94818209 | A/G | 0.060 | 0.111 | 0.094 | 0.238 | 37.787 |
| rs62017568 | Gastric cancer | 15 | 94725280 | T/C | 0.072 | 0.087 | 0.041 | 0.031 | 81.937 |
| rs72765429 | Gastric cancer | 15 | 94624210 | T/C | 0.070 | 0.052 | 0.158 | 0.744 | 41.356 |
| rs12911308 | Gastric cancer | 15 | 94802899 | G/A | 0.171 | 0.051 | 0.059 | 0.386 | 48.614 |
| rs28565137 | Gastric cancer | 15 | 94749470 | T/C | 0.168 | 0.049 | 0.035 | 0.163 | 33.504 |
| rs12595780 | Gastric cancer | 15 | 94776265 | T/G | 0.199 | 0.048 | 0.057 | 0.401 | 40.295 |
| rs76983840 | Gastric cancer | 15 | 94905666 | A/C | 0.110 | 0.034 | 0.020 | 0.098 | 34.981 |
| rs73456153 | Gastric cancer | 15 | 94675082 | A/G | 0.085 | 0.024 | 0.038 | 0.527 | 29.648 |
| rs74740981 | Gastric cancer | 15 | 94702358 | A/C | 0.049 | 0.023 | 0.102 | 0.826 | 43.225 |
| rs58548127 | Gastric cancer | 15 | 94720163 | C/T | 0.211 | 0.022 | 0.055 | 0.693 | 45.717 |
| rs62015953 | Gastric cancer | 15 | 94714320 | G/A | 0.076 | 0.021 | 0.062 | 0.741 | 198.855 |
| rs62017702 | Gastric cancer | 15 | 95050089 | T/C | 0.189 | 0.016 | 0.021 | 0.447 | 48.521 |
| rs12904449 | Gastric cancer | 15 | 94815970 | A/C | 0.426 | 0.015 | 0.017 | 0.377 | 62.092 |
| rs1433943 | Gastric cancer | 15 | 94866380 | C/T | 0.585 | 0.015 | 0.017 | 0.377 | 42.871 |
| rs1563361 | Gastric cancer | 15 | 94829625 | T/C | 0.405 | 0.014 | 0.018 | 0.438 | 22.433 |
| rs7165881 | Gastric cancer | 15 | 94670432 | G/T | 0.472 | 0.014 | 0.016 | 0.378 | 86.603 |
| rs17711817 | Gastric cancer | 15 | 94742408 | T/C | 0.068 | 0.014 | 0.022 | 0.529 | 59.043 |
| rs7181323 | Gastric cancer | 15 | 95113239 | T/C | 0.588 | 0.010 | 0.019 | 0.594 | 30.770 |
| rs56695207 | Gastric cancer | 15 | 94645630 | A/C | 0.258 | 0.009 | 0.017 | 0.611 | 57.673 |
| rs72649567 | Gastric cancer | 15 | 94624477 | A/G | 0.126 | 0.008 | 0.121 | 0.946 | 32.962 |
| rs11856773 | Gastric cancer | 15 | 95119927 | T/G | 0.155 | 0.007 | 0.063 | 0.908 | 30.864 |
| rs8026826 | Gastric cancer | 15 | 94803988 | C/T | 0.655 | 0.006 | 0.017 | 0.712 | 81.691 |
| rs12437799 | Gastric cancer | 15 | 94714594 | G/A | 0.218 | 0.004 | 0.041 | 0.919 | 107.508 |
| rs2060375 | Gastric cancer | 15 | 94901418 | C/T | 0.411 | 0.002 | 0.016 | 0.905 | 41.789 |
| rs7173798 | Gastric cancer | 15 | 94719249 | C/T | 0.340 | 0.001 | 0.020 | 0.947 | 43.378 |
| rs1842268 | Gastric cancer | 15 | 94672562 | T/C | 0.139 | 0.001 | 0.020 | 0.957 | 31.009 |
| rs11853883 | Gastric cancer | 15 | 94719916 | A/G | 0.335 | -0.001 | 0.019 | 0.958 | 57.788 |
| rs3743299 | Gastric cancer | 15 | 95024591 | T/C | 0.132 | -0.001 | 0.031 | 0.972 | 109.636 |
| rs8036334 | Gastric cancer | 15 | 94684390 | C/T | 0.278 | -0.001 | 0.022 | 0.953 | 117.117 |
| rs1424695 | Gastric cancer | 15 | 95014655 | C/T | 0.601 | -0.003 | 0.016 | 0.840 | 504.663 |
| rs12101859 | Gastric cancer | 15 | 94801343 | G/A | 0.470 | -0.005 | 0.017 | 0.772 | 32.928 |
| rs11635804 | Gastric cancer | 15 | 94871188 | C/T | 0.294 | -0.007 | 0.019 | 0.697 | 37.963 |
| rs12900180 | Gastric cancer | 15 | 94713013 | G/A | 0.545 | -0.008 | 0.016 | 0.629 | 168.335 |
| rs1981905 | Gastric cancer | 15 | 95093984 | G/A | 0.619 | -0.009 | 0.016 | 0.580 | 25.112 |
| rs6496017 | Gastric cancer | 15 | 95045906 | A/C | 0.524 | -0.012 | 0.017 | 0.476 | 171.501 |
| rs1378596 | Gastric cancer | 15 | 94704198 | C/T | 0.829 | -0.013 | 0.021 | 0.537 | 56.800 |
| rs996441 | Gastric cancer | 15 | 95086643 | T/G | 0.832 | -0.013 | 0.022 | 0.568 | 154.773 |
| rs6497172 | Gastric cancer | 15 | 94663438 | C/T | 0.391 | -0.013 | 0.017 | 0.438 | 30.522 |
| rs8037281 | Gastric cancer | 15 | 94956921 | C/T | 0.149 | -0.013 | 0.040 | 0.736 | 50.512 |
| rs180949208 | Gastric cancer | 15 | 94819275 | A/G | 0.049 | -0.015 | 0.108 | 0.889 | 25.899 |
| rs1080340 | Gastric cancer | 15 | 94676120 | G/T | 0.279 | -0.015 | 0.050 | 0.760 | 22.656 |
| rs62028067 | Gastric cancer | 15 | 94649941 | G/A | 0.152 | -0.016 | 0.025 | 0.524 | 48.990 |
| rs1080474 | Gastric cancer | 15 | 94726890 | G/A | 0.583 | -0.016 | 0.017 | 0.343 | 33.465 |
| rs6497177 | Gastric cancer | 15 | 94741598 | G/T | 0.795 | -0.016 | 0.028 | 0.561 | 20.368 |
| rs1433939 | Gastric cancer | 15 | 94968219 | T/C | 0.761 | -0.017 | 0.024 | 0.496 | 21.752 |
| rs1350092 | Gastric cancer | 15 | 94704086 | C/T | 0.143 | -0.017 | 0.028 | 0.545 | 151.305 |
| rs1031563 | Gastric cancer | 15 | 94748728 | G/A | 0.663 | -0.017 | 0.017 | 0.305 | 102.774 |
| rs4326999 | Gastric cancer | 15 | 95070370 | G/T | 0.415 | -0.017 | 0.016 | 0.275 | 37.765 |
| rs11856931 | Gastric cancer | 15 | 94715634 | T/C | 0.252 | -0.024 | 0.020 | 0.233 | 673.447 |
| rs11629840 | Gastric cancer | 15 | 94977176 | A/G | 0.238 | -0.029 | 0.020 | 0.144 | 57.577 |
| rs2304294 | Gastric cancer | 15 | 95001206 | C/T | 0.164 | -0.032 | 0.022 | 0.137 | 81.088 |
| rs8029074 | Gastric cancer | 15 | 94727654 | T/C | 0.938 | -0.042 | 0.027 | 0.119 | 41.324 |
| rs78852085 | Gastric cancer | 15 | 94749388 | A/G | 0.048 | -0.046 | 0.112 | 0.685 | 36.829 |
| rs11074277 | Gastric cancer | 15 | 95003251 | A/G | 0.031 | -0.048 | 0.128 | 0.707 | 39.557 |
| rs995762 | Gastric cancer | 15 | 94652054 | C/T | 0.860 | -0.058 | 0.065 | 0.370 | 32.710 |
| rs62017603 | Gastric cancer | 15 | 94749304 | A/G | 0.080 | -0.060 | 0.090 | 0.502 | 25.853 |
| rs72751339 | Gastric cancer | 15 | 94965410 | T/C | 0.062 | -0.064 | 0.092 | 0.488 | 38.683 |
| rs117049126 | Gastric cancer | 15 | 95023660 | C/T | 0.024 | -0.065 | 0.150 | 0.664 | 24.846 |
| rs111352825 | Gastric cancer | 15 | 94641234 | A/G | 0.029 | -0.074 | 0.136 | 0.589 | 30.780 |
| rs118191640 | Gastric cancer | 15 | 94678184 | A/C | 0.030 | -0.075 | 0.134 | 0.578 | 24.933 |
| rs12899267 | Gastric cancer | 15 | 94784606 | G/A | 0.158 | -0.092 | 0.062 | 0.136 | 65.120 |
| rs1424710 | Gastric cancer | 15 | 95072432 | G/A | 0.046 | -0.113 | 0.107 | 0.288 | 34.738 |
| rs62017660 | Gastric cancer | 15 | 94990118 | G/T | 0.035 | -0.124 | 0.123 | 0.316 | 25.602 |
| rs77465116 | Gastric cancer | 15 | 94804887 | T/C | 0.053 | -0.134 | 0.100 | 0.182 | 24.432 |
| rs66548062 | Gastric cancer | 15 | 94767385 | T/C | 0.058 | -0.148 | 0.176 | 0.402 | 38.344 |
| rs149629483 | Gastric cancer | 15 | 94958805 | G/A | 0.030 | -0.208 | 0.130 | 0.108 | 22.101 |
| CCDC88A |  |  |  |  |  |  |  |  |  |
| rs183468002 | Gastric cancer | 2 | 55556029 | A/C | 0.011 | 0.238 | 0.211 | 0.258 | 30.246 |
| rs74910171 | Gastric cancer | 2 | 55475987 | G/T | 0.023 | 0.176 | 0.149 | 0.236 | 21.742 |
| rs114715805 | Gastric cancer | 2 | 55880729 | T/G | 0.016 | 0.166 | 0.177 | 0.348 | 25.473 |
| rs115122235 | Gastric cancer | 2 | 55337171 | A/G | 0.035 | 0.156 | 0.122 | 0.200 | 54.643 |
| rs115020553 | Gastric cancer | 2 | 55275560 | A/G | 0.026 | 0.053 | 0.139 | 0.705 | 42.986 |
| rs13401937 | Gastric cancer | 2 | 55252214 | G/T | 0.071 | 0.025 | 0.069 | 0.714 | 58.205 |
| rs2589058 | Gastric cancer | 2 | 55510233 | C/T | 0.919 | 0.023 | 0.032 | 0.475 | 84.313 |
| rs782642 | Gastric cancer | 2 | 55928444 | T/G | 0.410 | 0.022 | 0.022 | 0.316 | 20.625 |
| rs2968781 | Gastric cancer | 2 | 55341952 | G/T | 0.854 | 0.015 | 0.019 | 0.438 | 20.439 |
| rs2920962 | Gastric cancer | 2 | 55508326 | T/C | 0.112 | 0.013 | 0.019 | 0.486 | 125.568 |
| rs150412347 | Gastric cancer | 2 | 55512936 | A/G | 0.151 | 0.011 | 0.018 | 0.535 | 456.075 |
| rs60376483 | Gastric cancer | 2 | 55506076 | G/T | 0.118 | 0.009 | 0.018 | 0.610 | 153.097 |
| rs7349405 | Gastric cancer | 2 | 55391294 | A/C | 0.166 | 0.007 | 0.018 | 0.685 | 79.759 |
| rs66728407 | Gastric cancer | 2 | 55521039 | G/A | 0.177 | 0.006 | 0.017 | 0.708 | 41.419 |
| rs2589096 | Gastric cancer | 2 | 55479163 | C/T | 0.685 | 0.005 | 0.016 | 0.741 | 36.817 |
| rs59783675 | Gastric cancer | 2 | 55492154 | A/G | 0.156 | 0.004 | 0.018 | 0.805 | 94.736 |
| rs2576696 | Gastric cancer | 2 | 55505681 | G/T | 0.794 | -0.001 | 0.017 | 0.951 | 152.453 |
| rs1045613 | Gastric cancer | 2 | 55516090 | T/C | 0.306 | -0.002 | 0.016 | 0.884 | 94.109 |
| rs12471379 | Gastric cancer | 2 | 55308451 | A/G | 0.311 | -0.003 | 0.018 | 0.889 | 33.840 |
| rs77147393 | Gastric cancer | 2 | 55379042 | T/C | 0.028 | -0.007 | 0.055 | 0.902 | 49.591 |
| rs6747649 | Gastric cancer | 2 | 55651140 | A/C | 0.338 | -0.009 | 0.016 | 0.588 | 101.444 |
| rs2920854 | Gastric cancer | 2 | 55351010 | C/T | 0.592 | -0.013 | 0.016 | 0.420 | 25.512 |
| rs6545489 | Gastric cancer | 2 | 55632750 | C/T | 0.862 | -0.020 | 0.019 | 0.287 | 515.747 |
| rs6718247 | Gastric cancer | 2 | 55663338 | A/G | 0.908 | -0.020 | 0.020 | 0.307 | 146.778 |
| rs76876962 | Gastric cancer | 2 | 55796095 | A/G | 0.040 | -0.020 | 0.047 | 0.669 | 129.857 |
| rs2627761 | Gastric cancer | 2 | 55933014 | T/C | 0.742 | -0.022 | 0.016 | 0.180 | 54.486 |
| rs13003270 | Gastric cancer | 2 | 55796409 | C/T | 0.235 | -0.036 | 0.017 | 0.034 | 27.302 |
| rs114386931 | Gastric cancer | 2 | 55591017 | C/T | 0.085 | -0.041 | 0.082 | 0.612 | 31.018 |
| rs115514755 | Gastric cancer | 2 | 55333445 | T/C | 0.024 | -0.136 | 0.148 | 0.355 | 68.640 |
| rs115114810 | Gastric cancer | 2 | 55902237 | G/A | 0.019 | -0.207 | 0.165 | 0.210 | 83.147 |
| rs17432206 | Gastric cancer | 2 | 55469930 | C/T | 0.020 | -0.245 | 0.162 | 0.129 | 63.066 |
| rs2903704 | Gastric cancer | 2 | 55805440 | C/T | 0.980 | -0.289 | 0.157 | 0.066 | 48.165 |
| rs782616 | Gastric cancer | 2 | 55909188 | G/T | 0.980 | -0.311 | 0.161 | 0.053 | 39.382 |
| ARHGAP21 |  |  |  |  |  |  |  |  |  |
| rs11815592 | Gastric cancer | 10 | 24994155 | A/G | 0.140 | 0.062 | 0.064 | 0.329 | 80.836 |
| rs1045873 | Gastric cancer | 10 | 25137772 | C/A | 0.360 | 0.036 | 0.019 | 0.058 | 32.133 |
| rs10764495 | Gastric cancer | 10 | 25062755 | C/T | 0.301 | 0.033 | 0.023 | 0.154 | 207.716 |
| rs16925127 | Gastric cancer | 10 | 25179312 | A/G | 0.069 | 0.025 | 0.022 | 0.246 | 30.444 |
| rs10828674 | Gastric cancer | 10 | 24859010 | G/A | 0.624 | 0.020 | 0.018 | 0.261 | 41.161 |
| rs35579040 | Gastric cancer | 10 | 24860804 | G/A | 0.193 | 0.020 | 0.024 | 0.406 | 47.292 |
| rs11014230 | Gastric cancer | 10 | 25071469 | A/G | 0.762 | 0.018 | 0.026 | 0.481 | 25.834 |
| rs4748968 | Gastric cancer | 10 | 25008912 | C/T | 0.773 | 0.015 | 0.018 | 0.400 | 96.327 |
| rs10734055 | Gastric cancer | 10 | 24996710 | T/C | 0.468 | 0.013 | 0.017 | 0.433 | 208.017 |
| rs7091639 | Gastric cancer | 10 | 25211324 | C/A | 0.237 | 0.007 | 0.017 | 0.673 | 36.705 |
| rs6482407 | Gastric cancer | 10 | 24882761 | A/G | 0.463 | 0.007 | 0.017 | 0.696 | 205.343 |
| rs12241993 | Gastric cancer | 10 | 25013050 | A/G | 0.255 | 0.005 | 0.016 | 0.759 | 27.064 |
| rs2001237 | Gastric cancer | 10 | 24863020 | C/T | 0.366 | 0.003 | 0.016 | 0.866 | 202.852 |
| rs71493396 | Gastric cancer | 10 | 25018251 | T/C | 0.046 | 0.003 | 0.040 | 0.950 | 20.246 |
| rs7901000 | Gastric cancer | 10 | 25184541 | G/T | 0.874 | 0.000 | 0.019 | 0.997 | 24.354 |
| rs10764508 | Gastric cancer | 10 | 25181404 | C/T | 0.435 | 0.000 | 0.016 | 0.989 | 19.626 |
| rs10828673 | Gastric cancer | 10 | 24852177 | A/G | 0.364 | -0.001 | 0.016 | 0.950 | 43.812 |
| rs10828731 | Gastric cancer | 10 | 25231515 | T/C | 0.476 | -0.002 | 0.016 | 0.909 | 28.294 |
| rs2804396 | Gastric cancer | 10 | 24854070 | T/G | 0.466 | -0.006 | 0.016 | 0.704 | 25.385 |
| rs4747502 | Gastric cancer | 10 | 25101633 | G/A | 0.624 | -0.011 | 0.016 | 0.493 | 31.436 |
| rs11593521 | Gastric cancer | 10 | 25081855 | C/T | 0.237 | -0.031 | 0.016 | 0.054 | 50.457 |
| rs61854259 | Gastric cancer | 10 | 24884510 | G/A | 0.079 | -0.033 | 0.029 | 0.260 | 23.954 |
| rs7477503 | Gastric cancer | 10 | 24903987 | C/T | 0.877 | -0.034 | 0.068 | 0.621 | 67.819 |
| rs12762735 | Gastric cancer | 10 | 24824565 | A/G | 0.363 | -0.036 | 0.022 | 0.107 | 28.707 |
| rs16925138 | Gastric cancer | 10 | 25181968 | G/A | 0.067 | -0.036 | 0.053 | 0.491 | 57.915 |
| rs12246876 | Gastric cancer | 10 | 25227403 | C/T | 0.225 | -0.038 | 0.041 | 0.358 | 32.091 |
| rs12415644 | Gastric cancer | 10 | 24882558 | G/A | 0.074 | -0.040 | 0.086 | 0.641 | 45.624 |
| rs12767180 | Gastric cancer | 10 | 25233083 | A/G | 0.137 | -0.043 | 0.035 | 0.218 | 49.032 |
| rs34180426 | Gastric cancer | 10 | 25030061 | C/T | 0.073 | -0.053 | 0.085 | 0.534 | 40.216 |
| rs2225781 | Gastric cancer | 10 | 25059475 | G/A | 0.936 | -0.059 | 0.091 | 0.514 | 30.358 |
| rs77846986 | Gastric cancer | 10 | 24974815 | G/A | 0.057 | -0.063 | 0.097 | 0.518 | 22.538 |
| rs111702766 | Gastric cancer | 10 | 24952028 | T/C | 0.039 | -0.092 | 0.115 | 0.425 | 19.781 |
| rs72793551 | Gastric cancer | 10 | 25040901 | C/A | 0.051 | -0.118 | 0.102 | 0.249 | 21.087 |
| rs74640492 | Gastric cancer | 10 | 25170302 | G/T | 0.029 | -0.156 | 0.135 | 0.247 | 26.260 |
| ZNHIT6 |  |  |  |  |  |  |  |  |  |
| rs146579293 | Gastric cancer | 1 | 86417798 | C/T | 0.018 | 0.481 | 0.175 | 0.006 | 19.697 |
| rs115352121 | Gastric cancer | 1 | 86054046 | T/C | 0.014 | 0.320 | 0.202 | 0.113 | 42.591 |
| rs140723277 | Gastric cancer | 1 | 86046600 | G/T | 0.014 | 0.310 | 0.197 | 0.115 | 26.547 |
| rs114031885 | Gastric cancer | 1 | 86379315 | A/G | 0.021 | 0.299 | 0.159 | 0.060 | 47.340 |
| rs141375517 | Gastric cancer | 1 | 86255018 | C/T | 0.013 | 0.282 | 0.203 | 0.166 | 41.090 |
| rs77754659 | Gastric cancer | 1 | 85955124 | A/G | 0.057 | 0.279 | 0.099 | 0.005 | 47.442 |
| rs61783744 | Gastric cancer | 1 | 86142451 | G/T | 0.039 | 0.217 | 0.119 | 0.069 | 87.417 |
| rs116021569 | Gastric cancer | 1 | 86180879 | A/G | 0.023 | 0.209 | 0.150 | 0.165 | 28.339 |
| rs35234617 | Gastric cancer | 1 | 86049527 | A/G | 0.031 | 0.169 | 0.128 | 0.188 | 31.194 |
| rs17400906 | Gastric cancer | 1 | 86228001 | A/C | 0.030 | 0.162 | 0.130 | 0.214 | 24.374 |
| rs114781207 | Gastric cancer | 1 | 86067818 | A/G | 0.012 | 0.134 | 0.208 | 0.520 | 24.647 |
| rs71654706 | Gastric cancer | 1 | 85983660 | A/G | 0.086 | 0.119 | 0.080 | 0.138 | 31.774 |
| rs77480927 | Gastric cancer | 1 | 86448174 | T/C | 0.013 | 0.108 | 0.213 | 0.610 | 27.119 |
| rs17128018 | Gastric cancer | 1 | 86101704 | G/T | 0.061 | 0.108 | 0.092 | 0.239 | 99.842 |
| rs115447761 | Gastric cancer | 1 | 86325732 | A/G | 0.039 | 0.075 | 0.138 | 0.588 | 40.221 |
| rs142918006 | Gastric cancer | 1 | 86399538 | C/A | 0.027 | 0.059 | 0.136 | 0.664 | 33.941 |
| rs142017786 | Gastric cancer | 1 | 86141757 | T/C | 0.038 | 0.049 | 0.118 | 0.674 | 58.895 |
| rs61783134 | Gastric cancer | 1 | 86242080 | T/G | 0.107 | 0.046 | 0.073 | 0.529 | 237.882 |
| rs75241249 | Gastric cancer | 1 | 86322310 | A/G | 0.031 | 0.046 | 0.047 | 0.324 | 33.942 |
| rs114636322 | Gastric cancer | 1 | 86328486 | C/T | 0.021 | 0.044 | 0.163 | 0.788 | 25.188 |
| rs145616941 | Gastric cancer | 1 | 86013356 | C/T | 0.048 | 0.041 | 0.104 | 0.697 | 53.780 |
| rs61787165 | Gastric cancer | 1 | 86181860 | C/T | 0.188 | 0.033 | 0.034 | 0.329 | 364.241 |
| rs78468242 | Gastric cancer | 1 | 86192219 | G/A | 0.041 | 0.028 | 0.113 | 0.804 | 64.324 |
| rs11161712 | Gastric cancer | 1 | 86430497 | A/G | 0.012 | 0.026 | 0.172 | 0.878 | 30.681 |
| rs17398091 | Gastric cancer | 1 | 86072996 | A/G | 0.189 | 0.023 | 0.030 | 0.440 | 314.577 |
| rs570121 | Gastric cancer | 1 | 85901598 | T/C | 0.704 | 0.022 | 0.017 | 0.191 | 27.222 |
| rs17127810 | Gastric cancer | 1 | 85954509 | C/T | 0.105 | 0.022 | 0.073 | 0.765 | 89.229 |
| rs12736249 | Gastric cancer | 1 | 86433237 | T/C | 0.178 | 0.015 | 0.029 | 0.598 | 437.088 |
| rs116313857 | Gastric cancer | 1 | 86371963 | A/C | 0.084 | 0.014 | 0.025 | 0.571 | 40.808 |
| rs1524002 | Gastric cancer | 1 | 86011289 | A/G | 0.152 | 0.013 | 0.028 | 0.637 | 108.370 |
| rs184339 | Gastric cancer | 1 | 86305704 | T/G | 0.168 | 0.011 | 0.023 | 0.650 | 179.815 |
| rs72946655 | Gastric cancer | 1 | 86140343 | C/T | 0.073 | 0.010 | 0.022 | 0.659 | 53.566 |
| rs7530839 | Gastric cancer | 1 | 86312788 | T/C | 0.178 | 0.010 | 0.029 | 0.741 | 449.579 |
| rs116540842 | Gastric cancer | 1 | 86426460 | A/G | 0.076 | 0.009 | 0.032 | 0.781 | 67.623 |
| rs4949910 | Gastric cancer | 1 | 86058907 | A/C | 0.151 | 0.008 | 0.017 | 0.636 | 109.061 |
| rs6661976 | Gastric cancer | 1 | 85960515 | T/C | 0.450 | 0.008 | 0.017 | 0.642 | 45.046 |
| rs10873708 | Gastric cancer | 1 | 86067742 | A/G | 0.313 | 0.007 | 0.017 | 0.679 | 184.084 |
| rs36056288 | Gastric cancer | 1 | 86106292 | C/T | 0.100 | 0.006 | 0.025 | 0.813 | 122.301 |
| rs67597676 | Gastric cancer | 1 | 86235043 | A/G | 0.101 | 0.005 | 0.025 | 0.845 | 118.074 |
| rs9700177 | Gastric cancer | 1 | 86151358 | T/C | 0.475 | 0.004 | 0.016 | 0.822 | 1086.687 |
| rs17390740 | Gastric cancer | 1 | 86014981 | G/A | 0.141 | 0.003 | 0.025 | 0.898 | 210.161 |
| rs76710587 | Gastric cancer | 1 | 86201744 | T/C | 0.165 | -0.001 | 0.018 | 0.947 | 126.236 |
| rs10493770 | Gastric cancer | 1 | 86034864 | C/T | 0.150 | -0.002 | 0.019 | 0.931 | 37.037 |
| rs366214 | Gastric cancer | 1 | 86250471 | C/T | 0.477 | -0.004 | 0.017 | 0.828 | 492.330 |
| rs116670580 | Gastric cancer | 1 | 86099059 | T/C | 0.020 | -0.004 | 0.163 | 0.978 | 22.684 |
| rs7543409 | Gastric cancer | 1 | 86049823 | T/C | 0.454 | -0.005 | 0.016 | 0.730 | 1024.853 |
| rs7354850 | Gastric cancer | 1 | 86094010 | A/G | 0.224 | -0.006 | 0.018 | 0.745 | 392.626 |
| rs1842580 | Gastric cancer | 1 | 86289328 | G/A | 0.056 | -0.006 | 0.097 | 0.950 | 69.361 |
| rs1524004 | Gastric cancer | 1 | 86011057 | C/T | 0.342 | -0.008 | 0.017 | 0.632 | 51.984 |
| rs145927758 | Gastric cancer | 1 | 86237860 | G/A | 0.092 | -0.008 | 0.025 | 0.737 | 53.154 |
| rs12086058 | Gastric cancer | 1 | 86040054 | G/A | 0.423 | -0.010 | 0.016 | 0.523 | 35.957 |
| rs522075 | Gastric cancer | 1 | 86446523 | T/C | 0.531 | -0.011 | 0.016 | 0.489 | 154.958 |
| rs12031047 | Gastric cancer | 1 | 86463196 | G/A | 0.486 | -0.012 | 0.016 | 0.451 | 178.095 |
| rs2297140 | Gastric cancer | 1 | 86046561 | A/C | 0.505 | -0.014 | 0.016 | 0.370 | 20.026 |
| rs72710977 | Gastric cancer | 1 | 86344146 | T/C | 0.098 | -0.016 | 0.023 | 0.483 | 42.640 |
| rs6656229 | Gastric cancer | 1 | 86012143 | T/G | 0.613 | -0.017 | 0.019 | 0.369 | 97.873 |
| rs551379 | Gastric cancer | 1 | 85958322 | T/C | 0.301 | -0.017 | 0.019 | 0.373 | 67.238 |
| rs1378226 | Gastric cancer | 1 | 86038738 | G/A | 0.873 | -0.018 | 0.022 | 0.415 | 134.122 |
| rs116357557 | Gastric cancer | 1 | 86416747 | A/G | 0.022 | -0.022 | 0.152 | 0.884 | 23.801 |
| rs74541713 | Gastric cancer | 1 | 86098767 | C/T | 0.032 | -0.024 | 0.126 | 0.848 | 38.975 |
| rs686272 | Gastric cancer | 1 | 86433866 | T/C | 0.264 | -0.025 | 0.019 | 0.195 | 34.792 |
| rs41289759 | Gastric cancer | 1 | 86172032 | A/G | 0.029 | -0.027 | 0.134 | 0.837 | 20.537 |
| rs511373 | Gastric cancer | 1 | 85965534 | A/G | 0.120 | -0.031 | 0.069 | 0.647 | 22.582 |
| rs75030935 | Gastric cancer | 1 | 86011931 | A/G | 0.074 | -0.032 | 0.031 | 0.303 | 94.718 |
| rs61783058 | Gastric cancer | 1 | 85931756 | T/C | 0.055 | -0.032 | 0.069 | 0.638 | 23.427 |
| rs79466005 | Gastric cancer | 1 | 86177188 | A/C | 0.045 | -0.043 | 0.028 | 0.124 | 29.816 |
| rs113882752 | Gastric cancer | 1 | 85972032 | T/C | 0.037 | -0.044 | 0.119 | 0.714 | 55.227 |
| rs61783136 | Gastric cancer | 1 | 86263736 | A/G | 0.070 | -0.048 | 0.032 | 0.134 | 45.380 |
| rs12136353 | Gastric cancer | 1 | 86335362 | A/C | 0.115 | -0.051 | 0.058 | 0.379 | 82.900 |
| rs1094353 | Gastric cancer | 1 | 86109090 | A/C | 0.963 | -0.055 | 0.036 | 0.127 | 68.920 |
| rs138985640 | Gastric cancer | 1 | 86017838 | A/G | 0.023 | -0.074 | 0.090 | 0.414 | 27.839 |
| rs142716944 | Gastric cancer | 1 | 86107564 | T/C | 0.010 | -0.086 | 0.238 | 0.719 | 26.856 |
| rs114069383 | Gastric cancer | 1 | 86202990 | C/T | 0.075 | -0.086 | 0.084 | 0.307 | 749.608 |
| rs17394404 | Gastric cancer | 1 | 86095948 | A/C | 0.075 | -0.091 | 0.085 | 0.284 | 756.038 |
| rs17400787 | Gastric cancer | 1 | 86226352 | A/G | 0.161 | -0.091 | 0.061 | 0.137 | 182.747 |
| rs79159470 | Gastric cancer | 1 | 86407510 | C/T | 0.014 | -0.093 | 0.190 | 0.626 | 142.284 |
| rs821398 | Gastric cancer | 1 | 86105729 | C/T | 0.159 | -0.094 | 0.062 | 0.128 | 195.417 |
| rs41301261 | Gastric cancer | 1 | 85816224 | A/G | 0.030 | -0.097 | 0.090 | 0.285 | 54.578 |
| rs147534785 | Gastric cancer | 1 | 86165922 | C/T | 0.032 | -0.124 | 0.128 | 0.334 | 48.785 |
| rs61783714 | Gastric cancer | 1 | 86041612 | A/G | 0.031 | -0.131 | 0.132 | 0.323 | 31.273 |
| rs76408018 | Gastric cancer | 1 | 86265690 | T/C | 0.020 | -0.152 | 0.158 | 0.336 | 215.056 |
| rs115494310 | Gastric cancer | 1 | 86144211 | C/T | 0.018 | -0.181 | 0.165 | 0.274 | 22.287 |
| rs61785164 | Gastric cancer | 1 | 85963348 | A/G | 0.029 | -0.184 | 0.134 | 0.170 | 69.071 |
| rs147595171 | Gastric cancer | 1 | 86141748 | A/G | 0.019 | -0.197 | 0.167 | 0.239 | 218.126 |
| rs141429918 | Gastric cancer | 1 | 86401952 | C/T | 0.021 | -0.208 | 0.192 | 0.279 | 39.885 |
| rs151298349 | Gastric cancer | 1 | 86465268 | C/T | 0.026 | -0.326 | 0.144 | 0.023 | 151.327 |
| rs149950999 | Gastric cancer | 1 | 86322338 | T/C | 0.013 | -0.364 | 0.204 | 0.074 | 73.819 |
| DYRK2 |  |  |  |  |  |  |  |  |  |
| rs10748078 | Gastric cancer | 12 | 67866181 | G/A | 0.629 | 0.023 | 0.016 | 0.149 | 23.145 |
| rs10506556 | Gastric cancer | 12 | 67909566 | G/A | 0.073 | -0.017 | 0.087 | 0.843 | 24.256 |
| rs1882033 | Gastric cancer | 12 | 67819376 | G/A | 0.404 | -0.030 | 0.020 | 0.136 | 35.787 |
| VPS26A |  |  |  |  |  |  |  |  |  |
| rs142210339 | Gastric cancer | 10 | 70914628 | C/T | 0.007 | 0.397 | 0.291 | 0.173 | 29.474 |
| rs41279650 | Gastric cancer | 10 | 71005807 | A/G | 0.026 | 0.260 | 0.143 | 0.069 | 19.581 |
| rs72812190 | Gastric cancer | 10 | 70989210 | T/C | 0.158 | 0.169 | 0.061 | 0.006 | 46.652 |
| rs41299234 | Gastric cancer | 10 | 70672804 | T/C | 0.024 | 0.156 | 0.148 | 0.294 | 79.762 |
| rs149171905 | Gastric cancer | 10 | 70949592 | T/C | 0.098 | 0.109 | 0.079 | 0.168 | 113.546 |
| rs61868307 | Gastric cancer | 10 | 70803835 | G/A | 0.085 | 0.070 | 0.081 | 0.386 | 81.958 |
| rs79937321 | Gastric cancer | 10 | 70989918 | T/C | 0.091 | 0.056 | 0.057 | 0.326 | 21.847 |
| rs74233638 | Gastric cancer | 10 | 70935909 | A/G | 0.028 | 0.037 | 0.031 | 0.230 | 175.295 |
| rs12570170 | Gastric cancer | 10 | 70801833 | A/G | 0.150 | 0.034 | 0.018 | 0.054 | 29.381 |
| rs2855023 | Gastric cancer | 10 | 70846441 | A/G | 0.332 | 0.014 | 0.016 | 0.381 | 123.290 |
| rs2394527 | Gastric cancer | 10 | 70825489 | A/G | 0.267 | 0.014 | 0.017 | 0.402 | 44.100 |
| rs4603200 | Gastric cancer | 10 | 71169826 | C/T | 0.456 | 0.006 | 0.018 | 0.744 | 41.757 |
| rs10998460 | Gastric cancer | 10 | 70613280 | A/G | 0.196 | 0.005 | 0.021 | 0.806 | 43.870 |
| rs12268341 | Gastric cancer | 10 | 70981632 | G/A | 0.102 | 0.001 | 0.025 | 0.971 | 40.514 |
| rs6480384 | Gastric cancer | 10 | 70890757 | T/C | 0.761 | 0.000 | 0.016 | 0.996 | 257.507 |
| rs12240360 | Gastric cancer | 10 | 70869547 | T/C | 0.056 | -0.001 | 0.036 | 0.988 | 27.670 |
| rs61868712 | Gastric cancer | 10 | 71011147 | T/C | 0.260 | -0.001 | 0.020 | 0.956 | 60.608 |
| rs4145930 | Gastric cancer | 10 | 70998738 | A/G | 0.838 | -0.001 | 0.018 | 0.944 | 49.567 |
| rs55772345 | Gastric cancer | 10 | 71010696 | A/G | 0.237 | -0.002 | 0.023 | 0.941 | 54.581 |
| rs10998639 | Gastric cancer | 10 | 70967081 | C/T | 0.257 | -0.003 | 0.024 | 0.908 | 26.964 |
| rs7091301 | Gastric cancer | 10 | 70991847 | C/T | 0.627 | -0.003 | 0.020 | 0.884 | 31.456 |
| rs7097078 | Gastric cancer | 10 | 71084361 | A/G | 0.460 | -0.003 | 0.017 | 0.843 | 21.774 |
| rs116913403 | Gastric cancer | 10 | 71013976 | A/G | 0.024 | -0.009 | 0.148 | 0.952 | 58.252 |
| rs1163170 | Gastric cancer | 10 | 70606157 | T/C | 0.447 | -0.011 | 0.016 | 0.493 | 29.638 |
| rs35893997 | Gastric cancer | 10 | 70775959 | T/C | 0.059 | -0.011 | 0.098 | 0.912 | 29.836 |
| rs61868680 | Gastric cancer | 10 | 70996052 | T/C | 0.156 | -0.012 | 0.023 | 0.586 | 33.326 |
| rs12269215 | Gastric cancer | 10 | 70969743 | T/C | 0.051 | -0.015 | 0.040 | 0.707 | 21.115 |
| rs10509306 | Gastric cancer | 10 | 70645697 | T/C | 0.133 | -0.018 | 0.024 | 0.460 | 64.702 |
| rs12415599 | Gastric cancer | 10 | 70815810 | A/G | 0.183 | -0.019 | 0.024 | 0.436 | 54.740 |
| rs12766376 | Gastric cancer | 10 | 70745824 | T/C | 0.140 | -0.019 | 0.025 | 0.440 | 38.858 |
| rs2246961 | Gastric cancer | 10 | 70783128 | A/C | 0.519 | -0.019 | 0.016 | 0.227 | 26.291 |
| rs75558280 | Gastric cancer | 10 | 70759507 | A/G | 0.047 | -0.022 | 0.105 | 0.833 | 33.308 |
| rs2487710 | Gastric cancer | 10 | 70782126 | A/C | 0.101 | -0.024 | 0.026 | 0.366 | 38.379 |
| rs147625539 | Gastric cancer | 10 | 70795924 | A/G | 0.011 | -0.026 | 0.216 | 0.905 | 26.190 |
| rs41278534 | Gastric cancer | 10 | 70652559 | C/A | 0.033 | -0.032 | 0.129 | 0.807 | 20.500 |
| rs10998646 | Gastric cancer | 10 | 70976702 | T/G | 0.471 | -0.033 | 0.027 | 0.223 | 285.989 |
| rs10998503 | Gastric cancer | 10 | 70720518 | A/G | 0.030 | -0.035 | 0.157 | 0.825 | 136.932 |
| rs117201755 | Gastric cancer | 10 | 70685976 | G/A | 0.015 | -0.037 | 0.223 | 0.867 | 19.614 |
| rs138159849 | Gastric cancer | 10 | 70890100 | C/T | 0.025 | -0.044 | 0.145 | 0.764 | 31.431 |
| rs117372387 | Gastric cancer | 10 | 71019884 | T/C | 0.015 | -0.053 | 0.187 | 0.778 | 20.636 |
| rs117679105 | Gastric cancer | 10 | 70901423 | T/C | 0.023 | -0.054 | 0.150 | 0.719 | 128.911 |
| rs72812197 | Gastric cancer | 10 | 70991788 | A/G | 0.171 | -0.062 | 0.038 | 0.102 | 84.879 |
| rs78505997 | Gastric cancer | 10 | 71016609 | T/C | 0.075 | -0.068 | 0.084 | 0.420 | 39.684 |
| rs113486223 | Gastric cancer | 10 | 70810107 | C/A | 0.204 | -0.072 | 0.067 | 0.283 | 44.233 |
| rs10998535 | Gastric cancer | 10 | 70805373 | T/C | 0.164 | -0.082 | 0.044 | 0.063 | 119.242 |
| rs10998786 | Gastric cancer | 10 | 71208324 | G/A | 0.080 | -0.111 | 0.082 | 0.177 | 25.519 |
| rs72807614 | Gastric cancer | 10 | 70898757 | A/G | 0.086 | -0.117 | 0.081 | 0.149 | 114.758 |
| rs10998456 | Gastric cancer | 10 | 70600905 | A/G | 0.049 | -0.170 | 0.104 | 0.101 | 47.139 |
| rs117447349 | Gastric cancer | 10 | 71071841 | A/G | 0.017 | -0.210 | 0.174 | 0.226 | 45.798 |
| rs34878143 | Gastric cancer | 10 | 70830526 | C/T | 0.023 | -0.275 | 0.121 | 0.023 | 258.715 |
| NOP16 |  |  |  |  |  |  |  |  |  |
| rs141048893 | Gastric cancer | 5 | 175815591 | G/A | 0.012 | 0.259 | 0.207 | 0.210 | 138.912 |
| rs151190553 | Gastric cancer | 5 | 175627251 | T/C | 0.051 | 0.237 | 0.182 | 0.193 | 35.435 |
| rs2303668 | Gastric cancer | 5 | 175824324 | C/T | 0.258 | 0.121 | 0.062 | 0.052 | 223.880 |
| rs115075460 | Gastric cancer | 5 | 175597694 | A/G | 0.016 | 0.108 | 0.179 | 0.544 | 31.311 |
| rs13180761 | Gastric cancer | 5 | 175586684 | A/G | 0.024 | 0.078 | 0.147 | 0.598 | 93.842 |
| rs1065206 | Gastric cancer | 5 | 175811233 | T/C | 0.055 | 0.043 | 0.099 | 0.666 | 586.717 |
| rs12522767 | Gastric cancer | 5 | 175988599 | G/A | 0.701 | 0.037 | 0.017 | 0.032 | 23.504 |
| rs13168897 | Gastric cancer | 5 | 175821562 | C/T | 0.147 | 0.032 | 0.026 | 0.220 | 150.301 |
| rs80335911 | Gastric cancer | 5 | 175827883 | T/C | 0.019 | 0.027 | 0.164 | 0.870 | 152.913 |
| rs13158884 | Gastric cancer | 5 | 175806176 | T/G | 0.247 | 0.018 | 0.017 | 0.282 | 386.748 |
| rs73341044 | Gastric cancer | 5 | 175968827 | A/G | 0.040 | 0.017 | 0.116 | 0.884 | 65.206 |
| rs6556264 | Gastric cancer | 5 | 175759350 | G/A | 0.536 | 0.012 | 0.016 | 0.441 | 80.972 |
| rs17832192 | Gastric cancer | 5 | 175815215 | A/G | 0.018 | 0.011 | 0.170 | 0.946 | 21.587 |
| rs7442946 | Gastric cancer | 5 | 175843890 | C/T | 0.475 | 0.006 | 0.016 | 0.724 | 190.723 |
| rs28768716 | Gastric cancer | 5 | 175908862 | C/T | 0.163 | 0.005 | 0.025 | 0.831 | 28.469 |
| rs12514451 | Gastric cancer | 5 | 175910252 | C/T | 0.501 | 0.003 | 0.016 | 0.848 | 71.494 |
| rs62403343 | Gastric cancer | 5 | 175686929 | A/C | 0.080 | -0.002 | 0.100 | 0.987 | 420.217 |
| rs145558590 | Gastric cancer | 5 | 175713997 | A/G | 0.012 | -0.015 | 0.213 | 0.943 | 92.245 |
| rs147533356 | Gastric cancer | 5 | 175592333 | A/G | 0.029 | -0.024 | 0.134 | 0.856 | 26.093 |
| rs73803606 | Gastric cancer | 5 | 175788955 | T/C | 0.043 | -0.039 | 0.048 | 0.416 | 37.342 |
| rs140908454 | Gastric cancer | 5 | 175972940 | T/C | 0.019 | -0.056 | 0.165 | 0.734 | 104.784 |
| rs13176426 | Gastric cancer | 5 | 176040202 | A/C | 0.044 | -0.059 | 0.111 | 0.591 | 32.696 |
| rs186038037 | Gastric cancer | 5 | 175821724 | G/A | 0.030 | -0.061 | 0.131 | 0.639 | 51.528 |
| rs113070993 | Gastric cancer | 5 | 175864652 | G/T | 0.137 | -0.066 | 0.066 | 0.319 | 29.045 |
| rs13169024 | Gastric cancer | 5 | 175821626 | C/T | 0.075 | -0.081 | 0.089 | 0.366 | 30.677 |
| rs114649577 | Gastric cancer | 5 | 175987625 | A/G | 0.023 | -0.323 | 0.153 | 0.035 | 20.203 |
| FAM171A1 |  |  |  |  |  |  |  |  |  |
| rs79518149 | Gastric cancer | 10 | 15264816 | A/G | 0.015 | 0.398 | 0.191 | 0.037 | 117.488 |
| rs142753686 | Gastric cancer | 10 | 15373373 | C/T | 0.013 | 0.229 | 0.196 | 0.243 | 30.344 |
| rs182568385 | Gastric cancer | 10 | 15051633 | T/C | 0.033 | 0.138 | 0.126 | 0.274 | 24.751 |
| rs140272131 | Gastric cancer | 10 | 15132730 | T/C | 0.016 | 0.133 | 0.181 | 0.462 | 26.302 |
| rs118167032 | Gastric cancer | 10 | 15237970 | T/C | 0.040 | 0.100 | 0.117 | 0.395 | 24.126 |
| rs12354702 | Gastric cancer | 10 | 15205825 | T/C | 0.030 | 0.084 | 0.077 | 0.276 | 126.021 |
| rs192815849 | Gastric cancer | 10 | 15003703 | T/C | 0.046 | 0.075 | 0.128 | 0.558 | 21.568 |
| rs112415423 | Gastric cancer | 10 | 15093224 | A/G | 0.092 | 0.045 | 0.080 | 0.576 | 59.298 |
| rs79810469 | Gastric cancer | 10 | 15252763 | A/G | 0.050 | 0.040 | 0.103 | 0.702 | 143.590 |
| rs7096608 | Gastric cancer | 10 | 15199753 | C/T | 0.110 | 0.039 | 0.031 | 0.210 | 183.343 |
| rs12415975 | Gastric cancer | 10 | 15529694 | C/T | 0.177 | 0.039 | 0.030 | 0.197 | 27.710 |
| rs77595277 | Gastric cancer | 10 | 15298781 | T/C | 0.009 | 0.036 | 0.247 | 0.885 | 21.717 |
| rs7092478 | Gastric cancer | 10 | 15303983 | A/G | 0.092 | 0.029 | 0.080 | 0.714 | 44.668 |
| rs55706505 | Gastric cancer | 10 | 15024708 | T/G | 0.045 | 0.027 | 0.107 | 0.802 | 44.967 |
| rs11593133 | Gastric cancer | 10 | 14995149 | A/G | 0.282 | 0.022 | 0.019 | 0.253 | 25.770 |
| rs140367799 | Gastric cancer | 10 | 14980221 | A/G | 0.019 | 0.020 | 0.174 | 0.909 | 39.016 |
| rs7902605 | Gastric cancer | 10 | 15513874 | A/G | 0.876 | 0.019 | 0.022 | 0.392 | 25.325 |
| rs11259579 | Gastric cancer | 10 | 15295262 | T/C | 0.085 | 0.018 | 0.037 | 0.635 | 36.582 |
| rs10752349 | Gastric cancer | 10 | 15097616 | T/G | 0.712 | 0.017 | 0.034 | 0.607 | 55.027 |
| rs148048665 | Gastric cancer | 10 | 14979427 | A/G | 0.039 | 0.016 | 0.122 | 0.893 | 23.036 |
| rs6602799 | Gastric cancer | 10 | 15067768 | G/A | 0.465 | 0.016 | 0.017 | 0.336 | 29.193 |
| rs10737093 | Gastric cancer | 10 | 15185503 | T/C | 0.790 | 0.015 | 0.016 | 0.361 | 111.869 |
| rs7921084 | Gastric cancer | 10 | 15267411 | T/C | 0.341 | 0.015 | 0.016 | 0.346 | 387.571 |
| rs11259577 | Gastric cancer | 10 | 15292213 | A/G | 0.285 | 0.015 | 0.016 | 0.356 | 932.862 |
| rs4375345 | Gastric cancer | 10 | 15270635 | A/G | 0.317 | 0.013 | 0.018 | 0.467 | 21.609 |
| rs61842446 | Gastric cancer | 10 | 15107826 | C/T | 0.036 | 0.012 | 0.119 | 0.920 | 83.723 |
| rs4750597 | Gastric cancer | 10 | 15214583 | C/T | 0.419 | 0.012 | 0.016 | 0.466 | 349.280 |
| rs10906835 | Gastric cancer | 10 | 15180108 | A/C | 0.213 | 0.008 | 0.020 | 0.684 | 72.689 |
| rs4488097 | Gastric cancer | 10 | 15228299 | C/T | 0.222 | 0.006 | 0.019 | 0.772 | 202.581 |
| rs7072433 | Gastric cancer | 10 | 15231980 | C/T | 0.540 | 0.003 | 0.016 | 0.844 | 457.525 |
| rs11259606 | Gastric cancer | 10 | 15373313 | T/C | 0.049 | 0.003 | 0.102 | 0.978 | 40.432 |
| rs9423933 | Gastric cancer | 10 | 15271162 | G/A | 0.882 | 0.002 | 0.031 | 0.952 | 53.132 |
| rs11259576 | Gastric cancer | 10 | 15287493 | T/C | 0.114 | 0.002 | 0.029 | 0.960 | 49.042 |
| rs117164512 | Gastric cancer | 10 | 15298845 | T/C | 0.028 | 0.001 | 0.140 | 0.996 | 22.852 |
| rs75502583 | Gastric cancer | 10 | 15268878 | A/C | 0.181 | -0.001 | 0.019 | 0.953 | 31.891 |
| rs7098117 | Gastric cancer | 10 | 15518556 | G/T | 0.727 | -0.002 | 0.025 | 0.951 | 20.805 |
| rs12763978 | Gastric cancer | 10 | 15238886 | A/C | 0.390 | -0.002 | 0.016 | 0.900 | 1337.627 |
| rs4379735 | Gastric cancer | 10 | 15243361 | G/A | 0.232 | -0.003 | 0.017 | 0.863 | 308.827 |
| rs11259586 | Gastric cancer | 10 | 15310317 | G/T | 0.451 | -0.004 | 0.016 | 0.807 | 49.430 |
| rs77618325 | Gastric cancer | 10 | 15268153 | G/A | 0.037 | -0.005 | 0.028 | 0.872 | 511.727 |
| rs7906952 | Gastric cancer | 10 | 14985302 | C/T | 0.780 | -0.005 | 0.023 | 0.829 | 90.814 |
| rs4261189 | Gastric cancer | 10 | 15029753 | T/C | 0.338 | -0.007 | 0.017 | 0.676 | 88.315 |
| rs80273362 | Gastric cancer | 10 | 15252680 | T/C | 0.019 | -0.008 | 0.169 | 0.965 | 62.598 |
| rs11592551 | Gastric cancer | 10 | 15203947 | G/A | 0.085 | -0.008 | 0.082 | 0.922 | 76.391 |
| rs12354460 | Gastric cancer | 10 | 15305218 | T/C | 0.583 | -0.010 | 0.016 | 0.564 | 26.863 |
| rs10906854 | Gastric cancer | 10 | 15235658 | C/T | 0.414 | -0.010 | 0.017 | 0.541 | 133.127 |
| rs4584473 | Gastric cancer | 10 | 15266819 | T/C | 0.297 | -0.012 | 0.018 | 0.514 | 143.694 |
| rs45445395 | Gastric cancer | 10 | 15341120 | A/G | 0.149 | -0.014 | 0.033 | 0.673 | 60.963 |
| rs143127940 | Gastric cancer | 10 | 15408227 | C/T | 0.015 | -0.014 | 0.189 | 0.940 | 23.937 |
| rs7358063 | Gastric cancer | 10 | 15223432 | T/C | 0.804 | -0.016 | 0.021 | 0.449 | 194.414 |
| rs10796266 | Gastric cancer | 10 | 15251137 | C/T | 0.807 | -0.016 | 0.019 | 0.402 | 98.449 |
| rs78404782 | Gastric cancer | 10 | 15073741 | T/C | 0.020 | -0.019 | 0.161 | 0.905 | 36.889 |
| rs7898588 | Gastric cancer | 10 | 15023482 | T/G | 0.255 | -0.022 | 0.023 | 0.338 | 72.573 |
| rs10906852 | Gastric cancer | 10 | 15227664 | T/C | 0.452 | -0.023 | 0.017 | 0.175 | 35.517 |
| rs78366867 | Gastric cancer | 10 | 15472169 | T/C | 0.453 | -0.023 | 0.018 | 0.193 | 24.008 |
| rs10796267 | Gastric cancer | 10 | 15292006 | C/T | 0.393 | -0.025 | 0.019 | 0.180 | 415.045 |
| rs10796265 | Gastric cancer | 10 | 15228363 | G/A | 0.332 | -0.026 | 0.017 | 0.124 | 346.048 |
| rs61844197 | Gastric cancer | 10 | 15208024 | T/C | 0.112 | -0.027 | 0.026 | 0.302 | 67.844 |
| rs78457076 | Gastric cancer | 10 | 15187928 | T/C | 0.021 | -0.027 | 0.159 | 0.864 | 64.712 |
| rs72777980 | Gastric cancer | 10 | 15525677 | A/G | 0.031 | -0.031 | 0.130 | 0.813 | 20.726 |
| rs11259457 | Gastric cancer | 10 | 15107077 | A/G | 0.255 | -0.036 | 0.017 | 0.037 | 34.190 |
| rs112703914 | Gastric cancer | 10 | 15235657 | A/G | 0.060 | -0.047 | 0.040 | 0.243 | 147.975 |
| rs41284463 | Gastric cancer | 10 | 15175007 | G/A | 0.026 | -0.049 | 0.141 | 0.727 | 47.501 |
| rs80042086 | Gastric cancer | 10 | 15358569 | T/C | 0.044 | -0.051 | 0.109 | 0.643 | 56.800 |
| rs117093307 | Gastric cancer | 10 | 15227380 | A/G | 0.042 | -0.059 | 0.110 | 0.593 | 96.275 |
| rs113752228 | Gastric cancer | 10 | 15068301 | T/G | 0.064 | -0.062 | 0.039 | 0.114 | 24.901 |
| rs78106675 | Gastric cancer | 10 | 15124606 | C/T | 0.057 | -0.070 | 0.038 | 0.070 | 79.439 |
| rs35283424 | Gastric cancer | 10 | 15467330 | A/G | 0.075 | -0.073 | 0.086 | 0.402 | 48.715 |
| rs142348973 | Gastric cancer | 10 | 15224726 | T/C | 0.015 | -0.082 | 0.188 | 0.662 | 28.717 |
| rs117040982 | Gastric cancer | 10 | 15131133 | T/G | 0.136 | -0.086 | 0.084 | 0.308 | 20.393 |
| rs61842703 | Gastric cancer | 10 | 15386768 | C/T | 0.057 | -0.087 | 0.096 | 0.361 | 57.937 |
| rs79099768 | Gastric cancer | 10 | 15296492 | T/G | 0.021 | -0.089 | 0.160 | 0.577 | 31.014 |
| rs79858949 | Gastric cancer | 10 | 15204436 | A/G | 0.025 | -0.097 | 0.143 | 0.498 | 169.883 |
| rs138410783 | Gastric cancer | 10 | 15493068 | T/C | 0.042 | -0.121 | 0.113 | 0.284 | 52.602 |
| rs76761646 | Gastric cancer | 10 | 15527088 | A/G | 0.064 | -0.129 | 0.091 | 0.153 | 61.370 |
| rs77855078 | Gastric cancer | 10 | 15426631 | T/C | 0.016 | -0.139 | 0.145 | 0.339 | 28.536 |
| rs12411755 | Gastric cancer | 10 | 15377970 | C/T | 0.017 | -0.162 | 0.173 | 0.349 | 27.112 |
| rs74401662 | Gastric cancer | 10 | 15274708 | C/T | 0.047 | -0.168 | 0.106 | 0.112 | 161.885 |
| rs4414122 | Gastric cancer | 10 | 15226701 | C/T | 0.941 | -0.180 | 0.095 | 0.059 | 83.183 |
| rs144041380 | Gastric cancer | 10 | 15130549 | A/G | 0.019 | -0.222 | 0.167 | 0.185 | 65.235 |
| rs143200493 | Gastric cancer | 10 | 15333157 | G/A | 0.012 | -0.250 | 0.206 | 0.226 | 27.222 |
| rs144123077 | Gastric cancer | 10 | 15073536 | A/G | 0.025 | -0.437 | 0.143 | 0.002 | 122.498 |
| UBE2D1 |  |  |  |  |  |  |  |  |  |
| rs117330845 | Gastric cancer | 10 | 60326609 | G/T | 0.008 | 0.459 | 0.251 | 0.067 | 39.955 |
| rs113791707 | Gastric cancer | 10 | 59880651 | A/G | 0.011 | 0.376 | 0.220 | 0.088 | 26.377 |
| rs77352043 | Gastric cancer | 10 | 60130789 | T/C | 0.011 | 0.309 | 0.214 | 0.150 | 30.605 |
| rs1658433 | Gastric cancer | 10 | 60329012 | A/G | 0.026 | 0.264 | 0.141 | 0.061 | 67.549 |
| rs11006024 | Gastric cancer | 10 | 59800804 | A/G | 0.025 | 0.226 | 0.143 | 0.115 | 21.544 |
| rs150886376 | Gastric cancer | 10 | 60131137 | C/T | 0.022 | 0.222 | 0.155 | 0.153 | 136.883 |
| rs75585286 | Gastric cancer | 10 | 60167300 | G/A | 0.008 | 0.187 | 0.256 | 0.467 | 83.743 |
| rs140813029 | Gastric cancer | 10 | 60104814 | G/A | 0.057 | 0.146 | 0.095 | 0.125 | 139.133 |
| rs141474325 | Gastric cancer | 10 | 60307347 | T/C | 0.010 | 0.134 | 0.163 | 0.411 | 70.293 |
| rs78325627 | Gastric cancer | 10 | 60146061 | C/T | 0.021 | 0.118 | 0.163 | 0.469 | 73.219 |
| rs80063617 | Gastric cancer | 10 | 60129387 | G/A | 0.047 | 0.118 | 0.107 | 0.274 | 24.649 |
| rs61874053 | Gastric cancer | 10 | 60377438 | T/C | 0.115 | 0.103 | 0.071 | 0.148 | 213.586 |
| rs148894582 | Gastric cancer | 10 | 60380180 | G/A | 0.010 | 0.100 | 0.230 | 0.665 | 24.574 |
| rs149495396 | Gastric cancer | 10 | 59913976 | C/T | 0.013 | 0.093 | 0.204 | 0.650 | 35.186 |
| rs11815860 | Gastric cancer | 10 | 59918833 | G/A | 0.056 | 0.086 | 0.096 | 0.373 | 105.897 |
| rs12355138 | Gastric cancer | 10 | 60140709 | A/G | 0.208 | 0.069 | 0.055 | 0.212 | 1658.780 |
| rs2162464 | Gastric cancer | 10 | 60226124 | T/C | 0.077 | 0.062 | 0.083 | 0.454 | 430.249 |
| rs72800569 | Gastric cancer | 10 | 60342485 | C/T | 0.022 | 0.059 | 0.149 | 0.694 | 45.559 |
| rs12768525 | Gastric cancer | 10 | 60295397 | G/T | 0.115 | 0.054 | 0.070 | 0.436 | 271.877 |
| rs146241998 | Gastric cancer | 10 | 60203694 | T/G | 0.074 | 0.051 | 0.088 | 0.561 | 346.924 |
| rs117669234 | Gastric cancer | 10 | 60158598 | C/T | 0.022 | 0.047 | 0.152 | 0.759 | 116.971 |
| rs112325861 | Gastric cancer | 10 | 60262718 | G/A | 0.035 | 0.047 | 0.122 | 0.702 | 136.764 |
| rs12256572 | Gastric cancer | 10 | 59823697 | C/T | 0.072 | 0.040 | 0.092 | 0.666 | 50.250 |
| rs113263447 | Gastric cancer | 10 | 60015555 | T/C | 0.075 | 0.025 | 0.085 | 0.768 | 290.072 |
| rs117129341 | Gastric cancer | 10 | 59827326 | A/G | 0.028 | 0.022 | 0.138 | 0.876 | 132.736 |
| rs112841905 | Gastric cancer | 10 | 60199980 | A/G | 0.029 | 0.021 | 0.135 | 0.874 | 209.280 |
| rs10826151 | Gastric cancer | 10 | 59855400 | C/A | 0.535 | 0.020 | 0.018 | 0.261 | 169.011 |
| rs17635794 | Gastric cancer | 10 | 59907497 | T/C | 0.096 | 0.019 | 0.022 | 0.385 | 132.171 |
| rs72791504 | Gastric cancer | 10 | 59843994 | A/G | 0.058 | 0.019 | 0.096 | 0.843 | 94.989 |
| rs145605055 | Gastric cancer | 10 | 60170759 | G/T | 0.022 | 0.018 | 0.161 | 0.913 | 114.304 |
| rs56356712 | Gastric cancer | 10 | 60211412 | C/T | 0.046 | 0.017 | 0.107 | 0.874 | 76.459 |
| rs2277256 | Gastric cancer | 10 | 60147934 | C/T | 0.044 | 0.010 | 0.041 | 0.800 | 202.456 |
| rs12264219 | Gastric cancer | 10 | 60230988 | A/G | 0.331 | 0.008 | 0.016 | 0.610 | 175.158 |
| rs28734985 | Gastric cancer | 10 | 60084051 | G/A | 0.090 | 0.008 | 0.028 | 0.773 | 38.351 |
| rs10763548 | Gastric cancer | 10 | 60245684 | G/A | 0.613 | 0.008 | 0.018 | 0.667 | 221.562 |
| rs10826143 | Gastric cancer | 10 | 59797131 | T/C | 0.602 | 0.008 | 0.017 | 0.658 | 124.474 |
| rs4468302 | Gastric cancer | 10 | 60164572 | C/T | 0.978 | 0.006 | 0.150 | 0.966 | 67.030 |
| rs7088198 | Gastric cancer | 10 | 60024832 | G/T | 0.051 | 0.006 | 0.101 | 0.954 | 109.071 |
| rs10826205 | Gastric cancer | 10 | 60371606 | A/G | 0.392 | 0.004 | 0.019 | 0.856 | 239.832 |
| rs80303277 | Gastric cancer | 10 | 60090899 | C/A | 0.211 | 0.003 | 0.018 | 0.853 | 164.684 |
| rs79332978 | Gastric cancer | 10 | 60292587 | G/A | 0.022 | 0.002 | 0.152 | 0.991 | 70.401 |
| rs148804182 | Gastric cancer | 10 | 60239869 | T/C | 0.026 | -0.001 | 0.146 | 0.997 | 102.560 |
| rs56387347 | Gastric cancer | 10 | 60354649 | T/G | 0.343 | -0.001 | 0.016 | 0.938 | 179.346 |
| rs1769004 | Gastric cancer | 10 | 59936998 | G/A | 0.390 | -0.001 | 0.016 | 0.935 | 98.776 |
| rs1427215 | Gastric cancer | 10 | 60229347 | A/G | 0.185 | -0.002 | 0.025 | 0.941 | 704.679 |
| rs7095923 | Gastric cancer | 10 | 60376952 | A/G | 0.039 | -0.002 | 0.046 | 0.958 | 70.114 |
| rs60656861 | Gastric cancer | 10 | 60079516 | T/C | 0.205 | -0.003 | 0.017 | 0.859 | 297.527 |
| rs2590366 | Gastric cancer | 10 | 60039293 | A/C | 0.391 | -0.003 | 0.016 | 0.835 | 95.273 |
| rs7089827 | Gastric cancer | 10 | 60157611 | T/C | 0.139 | -0.005 | 0.018 | 0.799 | 683.027 |
| rs79696318 | Gastric cancer | 10 | 60160637 | T/C | 0.094 | -0.005 | 0.018 | 0.766 | 25.839 |
| rs117181540 | Gastric cancer | 10 | 60233117 | A/G | 0.031 | -0.010 | 0.130 | 0.937 | 27.712 |
| rs139675989 | Gastric cancer | 10 | 60147776 | G/A | 0.016 | -0.010 | 0.187 | 0.956 | 108.566 |
| rs55935031 | Gastric cancer | 10 | 59861006 | A/C | 0.068 | -0.011 | 0.043 | 0.804 | 33.243 |
| rs11006205 | Gastric cancer | 10 | 60374087 | C/A | 0.095 | -0.011 | 0.028 | 0.685 | 270.892 |
| rs79646547 | Gastric cancer | 10 | 60364980 | T/C | 0.062 | -0.015 | 0.094 | 0.877 | 39.947 |
| rs12571250 | Gastric cancer | 10 | 60285619 | A/G | 0.083 | -0.016 | 0.023 | 0.498 | 119.579 |
| rs150718843 | Gastric cancer | 10 | 60207216 | C/T | 0.043 | -0.016 | 0.109 | 0.884 | 198.545 |
| rs2893777 | Gastric cancer | 10 | 60203919 | T/C | 0.688 | -0.018 | 0.017 | 0.295 | 141.917 |
| rs145668791 | Gastric cancer | 10 | 60361185 | G/A | 0.017 | -0.018 | 0.173 | 0.919 | 19.944 |
| rs4390300 | Gastric cancer | 10 | 60144207 | A/G | 0.461 | -0.022 | 0.016 | 0.162 | 7703.667 |
| rs17613571 | Gastric cancer | 10 | 59980752 | T/C | 0.024 | -0.023 | 0.155 | 0.884 | 55.018 |
| rs7907087 | Gastric cancer | 10 | 60006748 | G/A | 0.174 | -0.028 | 0.023 | 0.230 | 31.324 |
| rs80084991 | Gastric cancer | 10 | 59805791 | A/G | 0.023 | -0.029 | 0.159 | 0.855 | 46.998 |
| rs112375844 | Gastric cancer | 10 | 60107624 | A/G | 0.121 | -0.030 | 0.069 | 0.658 | 368.292 |
| rs189420211 | Gastric cancer | 10 | 60355343 | G/A | 0.014 | -0.038 | 0.194 | 0.845 | 51.877 |
| rs11594915 | Gastric cancer | 10 | 59831840 | T/C | 0.133 | -0.038 | 0.066 | 0.561 | 347.993 |
| rs1211325 | Gastric cancer | 10 | 59944457 | C/A | 0.406 | -0.039 | 0.019 | 0.041 | 37.761 |
| rs4948291 | Gastric cancer | 10 | 60100089 | T/C | 0.278 | -0.042 | 0.024 | 0.084 | 1349.394 |
| rs79613400 | Gastric cancer | 10 | 60243002 | G/A | 0.057 | -0.042 | 0.096 | 0.663 | 39.102 |
| rs150266451 | Gastric cancer | 10 | 60105830 | A/G | 0.028 | -0.046 | 0.139 | 0.742 | 141.648 |
| rs9971282 | Gastric cancer | 10 | 60142116 | T/C | 0.167 | -0.048 | 0.020 | 0.019 | 1014.544 |
| rs72797429 | Gastric cancer | 10 | 60145774 | G/A | 0.010 | -0.048 | 0.230 | 0.835 | 36.907 |
| rs117345913 | Gastric cancer | 10 | 60395834 | G/A | 0.027 | -0.051 | 0.136 | 0.709 | 37.174 |
| rs1625716 | Gastric cancer | 10 | 59960083 | G/T | 0.069 | -0.059 | 0.027 | 0.031 | 197.920 |
| rs16912322 | Gastric cancer | 10 | 60240744 | G/T | 0.075 | -0.059 | 0.086 | 0.488 | 47.791 |
| rs74573704 | Gastric cancer | 10 | 60221431 | T/C | 0.031 | -0.060 | 0.128 | 0.642 | 99.658 |
| rs73288325 | Gastric cancer | 10 | 60135028 | G/A | 0.019 | -0.060 | 0.164 | 0.715 | 86.027 |
| rs117768168 | Gastric cancer | 10 | 60220550 | C/A | 0.012 | -0.063 | 0.211 | 0.765 | 25.053 |
| rs72797428 | Gastric cancer | 10 | 60118664 | A/G | 0.014 | -0.068 | 0.206 | 0.740 | 30.394 |
| rs16912173 | Gastric cancer | 10 | 60141574 | C/T | 0.020 | -0.070 | 0.159 | 0.661 | 179.989 |
| rs17693054 | Gastric cancer | 10 | 60056178 | G/A | 0.039 | -0.071 | 0.115 | 0.537 | 249.812 |
| rs72800551 | Gastric cancer | 10 | 60310904 | G/T | 0.057 | -0.078 | 0.098 | 0.423 | 20.907 |
| rs17710296 | Gastric cancer | 10 | 59955178 | T/C | 0.040 | -0.080 | 0.114 | 0.483 | 229.320 |
| rs147600477 | Gastric cancer | 10 | 60219357 | C/T | 0.010 | -0.080 | 0.235 | 0.733 | 63.021 |
| rs55867717 | Gastric cancer | 10 | 59868926 | T/C | 0.046 | -0.083 | 0.107 | 0.437 | 46.142 |
| rs117283045 | Gastric cancer | 10 | 59908864 | T/G | 0.031 | -0.084 | 0.135 | 0.533 | 101.992 |
| rs4948514 | Gastric cancer | 10 | 60210174 | C/T | 0.075 | -0.085 | 0.088 | 0.334 | 390.055 |
| rs1905456 | Gastric cancer | 10 | 60095143 | C/T | 0.964 | -0.088 | 0.121 | 0.467 | 97.111 |
| rs76617049 | Gastric cancer | 10 | 60221006 | T/C | 0.043 | -0.098 | 0.110 | 0.372 | 30.211 |
| rs80258040 | Gastric cancer | 10 | 59972502 | C/T | 0.048 | -0.104 | 0.106 | 0.327 | 144.998 |
| rs141538312 | Gastric cancer | 10 | 59827281 | T/C | 0.015 | -0.106 | 0.188 | 0.573 | 77.681 |
| rs118028997 | Gastric cancer | 10 | 60371386 | T/C | 0.019 | -0.107 | 0.164 | 0.514 | 73.452 |
| rs11819252 | Gastric cancer | 10 | 60398160 | A/G | 0.029 | -0.110 | 0.134 | 0.410 | 52.088 |
| rs183799361 | Gastric cancer | 10 | 60267651 | C/T | 0.036 | -0.114 | 0.119 | 0.336 | 92.569 |
| rs1201754 | Gastric cancer | 10 | 59899585 | G/A | 0.943 | -0.121 | 0.098 | 0.217 | 74.377 |
| rs76294626 | Gastric cancer | 10 | 60001515 | A/G | 0.048 | -0.128 | 0.105 | 0.225 | 165.215 |
| rs79497150 | Gastric cancer | 10 | 60076961 | G/A | 0.017 | -0.130 | 0.173 | 0.452 | 91.712 |
| rs4948476 | Gastric cancer | 10 | 60084621 | A/G | 0.041 | -0.140 | 0.115 | 0.222 | 100.289 |
| rs142878832 | Gastric cancer | 10 | 60225437 | G/A | 0.013 | -0.143 | 0.202 | 0.479 | 53.917 |
| rs77410133 | Gastric cancer | 10 | 60326732 | T/G | 0.014 | -0.144 | 0.188 | 0.444 | 19.541 |
| rs118048712 | Gastric cancer | 10 | 59967593 | T/C | 0.017 | -0.151 | 0.174 | 0.386 | 92.591 |
| rs77630441 | Gastric cancer | 10 | 60418276 | G/A | 0.013 | -0.212 | 0.201 | 0.290 | 20.616 |
| rs1339587 | Gastric cancer | 10 | 59978905 | G/A | 0.015 | -0.219 | 0.185 | 0.235 | 93.886 |
| rs117246644 | Gastric cancer | 10 | 60163779 | T/C | 0.020 | -0.280 | 0.162 | 0.084 | 92.047 |
| rs116865804 | Gastric cancer | 10 | 59883492 | C/T | 0.020 | -0.292 | 0.163 | 0.073 | 29.413 |
| rs117494284 | Gastric cancer | 10 | 60359969 | A/C | 0.022 | -0.299 | 0.152 | 0.048 | 25.665 |
| TMEM30A |  |  |  |  |  |  |  |  |  |
| rs77927642 | Gastric cancer | 6 | 76013726 | C/T | 0.033 | 0.278 | 0.124 | 0.026 | 48.592 |
| rs116857161 | Gastric cancer | 6 | 76114799 | G/A | 0.038 | 0.179 | 0.116 | 0.123 | 75.520 |
| rs117483456 | Gastric cancer | 6 | 76000222 | T/C | 0.039 | 0.160 | 0.116 | 0.167 | 29.384 |
| rs17710795 | Gastric cancer | 6 | 75877166 | G/A | 0.117 | 0.146 | 0.124 | 0.238 | 99.291 |
| rs56321644 | Gastric cancer | 6 | 75991390 | T/G | 0.081 | 0.075 | 0.082 | 0.363 | 63.746 |
| rs9341521 | Gastric cancer | 6 | 76167360 | A/C | 0.198 | 0.069 | 0.068 | 0.312 | 49.237 |
| rs62414186 | Gastric cancer | 6 | 76088531 | A/G | 0.015 | 0.046 | 0.189 | 0.807 | 29.816 |
| rs11753997 | Gastric cancer | 6 | 75852777 | T/C | 0.095 | 0.043 | 0.081 | 0.599 | 51.126 |
| rs7766533 | Gastric cancer | 6 | 76279158 | A/G | 0.033 | 0.043 | 0.126 | 0.736 | 46.964 |
| rs13207335 | Gastric cancer | 6 | 76101047 | C/T | 0.067 | 0.038 | 0.089 | 0.674 | 46.828 |
| rs184766 | Gastric cancer | 6 | 75857907 | T/C | 0.251 | 0.038 | 0.021 | 0.067 | 123.906 |
| rs9360898 | Gastric cancer | 6 | 75953705 | G/T | 0.202 | 0.034 | 0.020 | 0.083 | 213.546 |
| rs9293999 | Gastric cancer | 6 | 76096230 | A/G | 0.218 | 0.023 | 0.020 | 0.228 | 177.856 |
| rs79466348 | Gastric cancer | 6 | 75976637 | A/C | 0.033 | 0.021 | 0.127 | 0.872 | 44.537 |
| rs240388 | Gastric cancer | 6 | 75972699 | G/A | 0.931 | 0.019 | 0.024 | 0.424 | 886.047 |
| rs6899513 | Gastric cancer | 6 | 76226430 | A/G | 0.278 | 0.017 | 0.034 | 0.611 | 45.550 |
| rs118062348 | Gastric cancer | 6 | 76140097 | T/C | 0.035 | 0.012 | 0.122 | 0.920 | 22.644 |
| rs4275021 | Gastric cancer | 6 | 76029760 | A/G | 0.575 | 0.012 | 0.016 | 0.447 | 276.469 |
| rs2748254 | Gastric cancer | 6 | 76014987 | G/A | 0.880 | 0.009 | 0.024 | 0.702 | 157.939 |
| rs56349005 | Gastric cancer | 6 | 76127908 | T/C | 0.180 | 0.006 | 0.027 | 0.826 | 83.748 |
| rs2842453 | Gastric cancer | 6 | 75928751 | C/A | 0.366 | -0.002 | 0.016 | 0.899 | 88.101 |
| rs1332019 | Gastric cancer | 6 | 75844700 | A/C | 0.285 | -0.005 | 0.020 | 0.800 | 21.899 |
| rs240374 | Gastric cancer | 6 | 75994151 | G/A | 0.088 | -0.014 | 0.024 | 0.552 | 98.067 |
| rs10755339 | Gastric cancer | 6 | 75999881 | T/C | 0.240 | -0.021 | 0.026 | 0.425 | 207.949 |
| rs13198422 | Gastric cancer | 6 | 75932139 | T/G | 0.213 | -0.028 | 0.028 | 0.309 | 27.439 |
| rs72877517 | Gastric cancer | 6 | 76183844 | A/G | 0.065 | -0.029 | 0.091 | 0.751 | 45.948 |
| rs79685806 | Gastric cancer | 6 | 75923886 | A/C | 0.044 | -0.030 | 0.033 | 0.368 | 21.065 |
| rs35116474 | Gastric cancer | 6 | 75879018 | C/T | 0.067 | -0.045 | 0.089 | 0.617 | 28.899 |
| rs72877560 | Gastric cancer | 6 | 76223725 | A/G | 0.077 | -0.050 | 0.040 | 0.206 | 63.233 |
| rs72883866 | Gastric cancer | 6 | 76070219 | A/C | 0.081 | -0.052 | 0.040 | 0.192 | 98.074 |
| rs9352210 | Gastric cancer | 6 | 76161550 | G/A | 0.481 | -0.064 | 0.054 | 0.235 | 315.753 |
| rs17409678 | Gastric cancer | 6 | 76105426 | A/G | 0.007 | -0.083 | 0.267 | 0.757 | 44.729 |
| rs79830845 | Gastric cancer | 6 | 76157150 | T/C | 0.027 | -0.090 | 0.138 | 0.514 | 21.470 |
| rs45596238 | Gastric cancer | 6 | 75965294 | A/C | 0.026 | -0.096 | 0.143 | 0.500 | 34.947 |
| rs113597915 | Gastric cancer | 6 | 75910374 | A/G | 0.067 | -0.329 | 0.158 | 0.038 | 27.276 |
| UBE2N |  |  |  |  |  |  |  |  |  |
| rs12830302 | Gastric cancer | 12 | 93812026 | T/C | 0.118 | 0.077 | 0.069 | 0.265 | 55.798 |
| rs11831347 | Gastric cancer | 12 | 93916714 | T/C | 0.089 | 0.061 | 0.079 | 0.441 | 35.280 |
| rs1846392 | Gastric cancer | 12 | 93889816 | A/C | 0.654 | 0.011 | 0.016 | 0.498 | 20.672 |
| rs10859492 | Gastric cancer | 12 | 93839087 | C/T | 0.478 | 0.001 | 0.016 | 0.956 | 41.691 |
| rs10777512 | Gastric cancer | 12 | 93859770 | C/T | 0.308 | -0.005 | 0.017 | 0.794 | 65.131 |
| PRR13 |  |  |  |  |  |  |  |  |  |
| rs145764255 | Gastric cancer | 12 | 53660182 | T/C | 0.025 | 0.167 | 0.182 | 0.360 | 33.891 |
| rs113283857 | Gastric cancer | 12 | 53920858 | A/G | 0.015 | 0.165 | 0.188 | 0.380 | 21.968 |
| rs75851863 | Gastric cancer | 12 | 53669376 | C/T | 0.056 | 0.155 | 0.098 | 0.113 | 38.528 |
| rs111337715 | Gastric cancer | 12 | 53800148 | T/C | 0.077 | 0.075 | 0.083 | 0.371 | 43.319 |
| rs1800634 | Gastric cancer | 12 | 54063493 | C/T | 0.716 | 0.063 | 0.050 | 0.205 | 21.746 |
| rs11612128 | Gastric cancer | 12 | 53632499 | G/A | 0.058 | 0.060 | 0.098 | 0.539 | 66.090 |
| rs17765360 | Gastric cancer | 12 | 54102784 | G/A | 0.218 | 0.050 | 0.028 | 0.071 | 28.557 |
| rs10783589 | Gastric cancer | 12 | 54013452 | C/T | 0.472 | 0.023 | 0.021 | 0.264 | 50.217 |
| rs4759289 | Gastric cancer | 12 | 54094324 | T/C | 0.340 | 0.023 | 0.017 | 0.164 | 21.972 |
| rs17751196 | Gastric cancer | 12 | 53718446 | G/A | 0.269 | 0.021 | 0.018 | 0.247 | 69.733 |
| rs3741658 | Gastric cancer | 12 | 54109733 | G/A | 0.352 | 0.010 | 0.016 | 0.529 | 32.972 |
| rs7975351 | Gastric cancer | 12 | 53902190 | A/G | 0.477 | 0.008 | 0.045 | 0.853 | 80.868 |
| rs116876770 | Gastric cancer | 12 | 53884232 | G/A | 0.031 | 0.007 | 0.129 | 0.958 | 36.085 |
| rs4759082 | Gastric cancer | 12 | 53734506 | T/C | 0.453 | 0.001 | 0.017 | 0.949 | 57.115 |
| rs11170488 | Gastric cancer | 12 | 53626940 | T/C | 0.309 | 0.000 | 0.016 | 0.988 | 23.590 |
| rs8938 | Gastric cancer | 12 | 53936878 | T/G | 0.117 | -0.009 | 0.028 | 0.736 | 236.841 |
| rs7313459 | Gastric cancer | 12 | 53878012 | G/A | 0.041 | -0.013 | 0.112 | 0.911 | 42.772 |
| rs11829355 | Gastric cancer | 12 | 53733283 | A/G | 0.193 | -0.019 | 0.019 | 0.311 | 190.831 |
| rs11170558 | Gastric cancer | 12 | 53835035 | T/G | 0.181 | -0.021 | 0.020 | 0.274 | 365.092 |
| rs12818931 | Gastric cancer | 12 | 54072180 | A/G | 0.116 | -0.026 | 0.027 | 0.335 | 199.397 |
| rs2683521 | Gastric cancer | 12 | 53799714 | A/G | 0.588 | -0.044 | 0.023 | 0.063 | 93.710 |
| rs191643352 | Gastric cancer | 12 | 53744158 | A/G | 0.014 | -0.048 | 0.187 | 0.799 | 32.506 |
| rs142356142 | Gastric cancer | 12 | 53678876 | C/T | 0.013 | -0.060 | 0.206 | 0.769 | 68.233 |
| ACBD6 |  |  |  |  |  |  |  |  |  |
| rs187433129 | Gastric cancer | 1 | 180487710 | G/A | 0.019 | 0.369 | 0.211 | 0.081 | 36.285 |
| rs115450958 | Gastric cancer | 1 | 180512237 | T/G | 0.030 | 0.189 | 0.165 | 0.251 | 27.144 |
| rs75468761 | Gastric cancer | 1 | 180250338 | G/T | 0.039 | 0.114 | 0.072 | 0.111 | 50.850 |
| rs76937829 | Gastric cancer | 1 | 180413890 | C/T | 0.041 | 0.100 | 0.071 | 0.158 | 54.635 |
| rs145551067 | Gastric cancer | 1 | 180242426 | G/A | 0.031 | 0.097 | 0.130 | 0.458 | 82.690 |
| rs114884529 | Gastric cancer | 1 | 180528177 | T/C | 0.022 | 0.083 | 0.061 | 0.176 | 56.306 |
| rs822724 | Gastric cancer | 1 | 180228611 | A/G | 0.061 | 0.054 | 0.049 | 0.274 | 79.916 |
| rs357045 | Gastric cancer | 1 | 180238844 | T/C | 0.073 | 0.049 | 0.044 | 0.270 | 237.729 |
| rs75219682 | Gastric cancer | 1 | 180719436 | T/C | 0.086 | 0.036 | 0.080 | 0.658 | 28.209 |
| rs4454504 | Gastric cancer | 1 | 180227844 | T/C | 0.064 | 0.029 | 0.049 | 0.560 | 64.672 |
| rs2254465 | Gastric cancer | 1 | 180257692 | C/A | 0.957 | 0.028 | 0.064 | 0.668 | 89.257 |
| rs2764447 | Gastric cancer | 1 | 180227223 | G/A | 0.225 | 0.026 | 0.018 | 0.150 | 21.090 |
| rs10914005 | Gastric cancer | 1 | 180445616 | T/C | 0.592 | 0.020 | 0.016 | 0.210 | 582.917 |
| rs77794519 | Gastric cancer | 1 | 180476996 | G/A | 0.080 | 0.018 | 0.083 | 0.829 | 72.954 |
| rs142452264 | Gastric cancer | 1 | 180306171 | G/T | 0.602 | 0.015 | 0.016 | 0.353 | 580.015 |
| rs7535761 | Gastric cancer | 1 | 180528198 | A/C | 0.585 | 0.014 | 0.020 | 0.498 | 67.858 |
| rs146286448 | Gastric cancer | 1 | 180538976 | A/G | 0.062 | 0.007 | 0.093 | 0.940 | 36.149 |
| rs7521151 | Gastric cancer | 1 | 180498916 | C/T | 0.104 | 0.006 | 0.026 | 0.832 | 101.008 |
| rs12025423 | Gastric cancer | 1 | 180269587 | C/T | 0.243 | 0.005 | 0.018 | 0.781 | 138.807 |
| rs10798783 | Gastric cancer | 1 | 180567428 | A/G | 0.641 | 0.004 | 0.016 | 0.787 | 76.073 |
| rs7543669 | Gastric cancer | 1 | 180242659 | T/G | 0.208 | 0.001 | 0.017 | 0.976 | 22.664 |
| rs357059 | Gastric cancer | 1 | 180227186 | G/A | 0.479 | -0.007 | 0.017 | 0.696 | 102.091 |
| rs61811564 | Gastric cancer | 1 | 180276958 | T/C | 0.028 | -0.008 | 0.136 | 0.952 | 46.355 |
| rs6677578 | Gastric cancer | 1 | 180534231 | A/G | 0.879 | -0.018 | 0.019 | 0.326 | 49.619 |
| rs357050 | Gastric cancer | 1 | 180234729 | C/T | 0.255 | -0.024 | 0.025 | 0.354 | 196.400 |
| rs61809302 | Gastric cancer | 1 | 180597303 | A/G | 0.100 | -0.031 | 0.074 | 0.673 | 22.315 |
| rs946634 | Gastric cancer | 1 | 180492477 | A/G | 0.939 | -0.034 | 0.043 | 0.426 | 175.263 |
| rs10798764 | Gastric cancer | 1 | 180387655 | G/A | 0.938 | -0.036 | 0.043 | 0.398 | 181.194 |
| rs4403592 | Gastric cancer | 1 | 180379692 | C/T | 0.063 | -0.040 | 0.030 | 0.188 | 116.202 |
| rs61809139 | Gastric cancer | 1 | 180228029 | T/C | 0.149 | -0.047 | 0.027 | 0.084 | 19.619 |
| rs147422799 | Gastric cancer | 1 | 180527139 | A/G | 0.014 | -0.049 | 0.191 | 0.798 | 20.265 |
| rs112362558 | Gastric cancer | 1 | 180359815 | A/G | 0.128 | -0.060 | 0.039 | 0.126 | 159.416 |
| rs61809110 | Gastric cancer | 1 | 180190121 | A/G | 0.101 | -0.063 | 0.075 | 0.399 | 31.388 |
| rs141409763 | Gastric cancer | 1 | 180275264 | C/A | 0.034 | -0.073 | 0.129 | 0.572 | 24.852 |
| rs112449051 | Gastric cancer | 1 | 180266077 | T/C | 0.095 | -0.074 | 0.076 | 0.332 | 87.765 |
| rs12145742 | Gastric cancer | 1 | 180388318 | A/G | 0.156 | -0.078 | 0.062 | 0.211 | 88.871 |
| rs17372764 | Gastric cancer | 1 | 180491279 | A/G | 0.093 | -0.086 | 0.077 | 0.266 | 88.157 |
| rs822723 | Gastric cancer | 1 | 180228968 | C/T | 0.858 | -0.087 | 0.039 | 0.026 | 71.883 |
| rs143547893 | Gastric cancer | 1 | 180371848 | T/C | 0.014 | -0.093 | 0.192 | 0.627 | 24.436 |
| rs115129266 | Gastric cancer | 1 | 180404725 | C/A | 0.035 | -0.105 | 0.121 | 0.387 | 22.803 |
| rs111276087 | Gastric cancer | 1 | 180353045 | C/T | 0.026 | -0.202 | 0.144 | 0.161 | 26.148 |
| TMCO1 |  |  |  |  |  |  |  |  |  |
| rs76656809 | Gastric cancer | 1 | 165802184 | A/G | 0.146 | 0.128 | 0.065 | 0.048 | 26.631 |
| rs77291389 | Gastric cancer | 1 | 165738583 | A/G | 0.015 | 0.109 | 0.156 | 0.487 | 28.330 |
| rs6426936 | Gastric cancer | 1 | 165684707 | C/T | 0.901 | 0.057 | 0.055 | 0.297 | 45.168 |
| rs56385641 | Gastric cancer | 1 | 165465823 | A/G | 0.080 | 0.027 | 0.030 | 0.358 | 22.966 |
| rs12047474 | Gastric cancer | 1 | 165679738 | C/T | 0.233 | 0.017 | 0.018 | 0.362 | 25.406 |
| rs75582843 | Gastric cancer | 1 | 165748493 | C/T | 0.718 | 0.003 | 0.019 | 0.887 | 92.501 |
| rs143863391 | Gastric cancer | 1 | 165786950 | A/G | 0.094 | -0.001 | 0.094 | 0.996 | 31.147 |
| rs10918271 | Gastric cancer | 1 | 165704732 | T/C | 0.264 | -0.003 | 0.020 | 0.880 | 25.775 |
| rs3186873 | Gastric cancer | 1 | 165693539 | C/T | 0.385 | -0.004 | 0.016 | 0.810 | 67.250 |
| rs10800110 | Gastric cancer | 1 | 165522014 | T/C | 0.654 | -0.027 | 0.018 | 0.127 | 32.063 |
| rs12079614 | Gastric cancer | 1 | 165799852 | G/A | 0.591 | -0.030 | 0.025 | 0.230 | 27.475 |
| rs12408101 | Gastric cancer | 1 | 165667781 | G/T | 0.281 | -0.132 | 0.050 | 0.008 | 25.294 |
| GOLPH3L |  |  |  |  |  |  |  |  |  |
| rs112580485 | Gastric cancer | 1 | 150626292 | A/G | 0.077 | 0.084 | 0.084 | 0.318 | 33.227 |
| rs17661357 | Gastric cancer | 1 | 150923099 | C/T | 0.077 | 0.056 | 0.083 | 0.503 | 44.645 |
| rs114529840 | Gastric cancer | 1 | 150457517 | G/T | 0.063 | 0.045 | 0.091 | 0.624 | 21.543 |
| rs149932571 | Gastric cancer | 1 | 150353670 | A/G | 0.064 | 0.041 | 0.090 | 0.649 | 21.201 |
| rs116173394 | Gastric cancer | 1 | 150609429 | A/G | 0.060 | 0.040 | 0.093 | 0.670 | 26.787 |
| rs74503525 | Gastric cancer | 1 | 150866833 | A/G | 0.010 | 0.033 | 0.230 | 0.886 | 93.606 |
| rs12046899 | Gastric cancer | 1 | 150336468 | T/G | 0.206 | 0.020 | 0.020 | 0.307 | 25.891 |
| rs11803940 | Gastric cancer | 1 | 150685835 | G/A | 0.394 | 0.011 | 0.016 | 0.497 | 93.460 |
| rs2089082 | Gastric cancer | 1 | 150800090 | G/T | 0.364 | 0.010 | 0.016 | 0.517 | 137.147 |
| rs11204682 | Gastric cancer | 1 | 150595537 | T/G | 0.187 | 0.010 | 0.025 | 0.686 | 24.226 |
| rs34985895 | Gastric cancer | 1 | 150932501 | G/A | 0.393 | 0.010 | 0.016 | 0.544 | 111.407 |
| rs10847 | Gastric cancer | 1 | 150782797 | T/C | 0.235 | 0.007 | 0.022 | 0.730 | 32.398 |
| rs72704686 | Gastric cancer | 1 | 150897800 | A/G | 0.080 | 0.007 | 0.032 | 0.835 | 37.110 |
| rs12406660 | Gastric cancer | 1 | 150836439 | C/T | 0.206 | 0.001 | 0.018 | 0.963 | 24.560 |
| rs12124948 | Gastric cancer | 1 | 150527294 | T/C | 0.439 | -0.014 | 0.016 | 0.365 | 36.216 |
| rs11204671 | Gastric cancer | 1 | 150554458 | C/T | 0.660 | -0.015 | 0.017 | 0.370 | 69.584 |
| rs56411234 | Gastric cancer | 1 | 150532376 | A/G | 0.029 | -0.024 | 0.136 | 0.862 | 24.932 |
| rs111690082 | Gastric cancer | 1 | 150572097 | T/C | 0.009 | -0.044 | 0.240 | 0.856 | 121.652 |
| rs6694394 | Gastric cancer | 1 | 150427934 | C/T | 0.221 | -0.083 | 0.054 | 0.121 | 30.980 |
| rs72704620 | Gastric cancer | 1 | 150738596 | A/G | 0.068 | -0.085 | 0.090 | 0.345 | 42.267 |
| rs116275309 | Gastric cancer | 1 | 150863650 | A/G | 0.045 | -0.088 | 0.106 | 0.409 | 28.611 |
| rs78646592 | Gastric cancer | 1 | 150599849 | T/C | 0.026 | -0.107 | 0.141 | 0.448 | 27.470 |
| rs75904649 | Gastric cancer | 1 | 150759873 | T/C | 0.009 | -0.112 | 0.233 | 0.632 | 102.732 |
| rs113673199 | Gastric cancer | 1 | 150330645 | G/A | 0.007 | -0.212 | 0.284 | 0.455 | 73.474 |
| STAT2 |  |  |  |  |  |  |  |  |  |
| rs117867773 | Gastric cancer | 12 | 56881614 | A/G | 0.056 | 0.147 | 0.097 | 0.129 | 179.113 |
| rs74703593 | Gastric cancer | 12 | 56585248 | T/C | 0.042 | 0.133 | 0.114 | 0.242 | 284.144 |
| rs187630978 | Gastric cancer | 12 | 56574256 | T/C | 0.011 | 0.119 | 0.284 | 0.675 | 91.474 |
| rs79222157 | Gastric cancer | 12 | 56480474 | A/C | 0.059 | 0.102 | 0.094 | 0.281 | 331.375 |
| rs77740732 | Gastric cancer | 12 | 57043143 | G/A | 0.058 | 0.089 | 0.097 | 0.361 | 88.420 |
| rs2933243 | Gastric cancer | 12 | 56860577 | A/G | 0.183 | 0.088 | 0.057 | 0.124 | 57.295 |
| rs61937687 | Gastric cancer | 12 | 56727242 | C/T | 0.083 | 0.070 | 0.082 | 0.395 | 27.066 |
| rs7312770 | Gastric cancer | 12 | 56467587 | T/C | 0.581 | 0.011 | 0.019 | 0.544 | 59.135 |
| rs150955176 | Gastric cancer | 12 | 56706421 | C/T | 0.057 | 0.005 | 0.039 | 0.894 | 568.049 |
| rs2292238 | Gastric cancer | 12 | 56493822 | C/A | 0.387 | 0.004 | 0.016 | 0.811 | 38.777 |
| rs117617338 | Gastric cancer | 12 | 56542132 | A/G | 0.015 | -0.002 | 0.095 | 0.980 | 50.034 |
| rs79269008 | Gastric cancer | 12 | 56614680 | G/A | 0.024 | -0.003 | 0.144 | 0.986 | 171.762 |
| rs34287626 | Gastric cancer | 12 | 56809264 | T/C | 0.467 | -0.003 | 0.016 | 0.872 | 27.527 |
| rs10876879 | Gastric cancer | 12 | 56609885 | A/G | 0.331 | -0.015 | 0.018 | 0.387 | 25.791 |
| rs79699236 | Gastric cancer | 12 | 56509546 | T/C | 0.017 | -0.016 | 0.173 | 0.926 | 59.693 |
| rs11171796 | Gastric cancer | 12 | 56692710 | T/C | 0.358 | -0.021 | 0.017 | 0.212 | 49.034 |
| rs56245751 | Gastric cancer | 12 | 56795415 | C/T | 0.180 | -0.022 | 0.025 | 0.383 | 111.465 |
| rs61939014 | Gastric cancer | 12 | 56622261 | T/C | 0.231 | -0.033 | 0.028 | 0.236 | 49.406 |
| rs73113010 | Gastric cancer | 12 | 56781920 | T/C | 0.021 | -0.106 | 0.156 | 0.496 | 81.119 |
| POLR3G |  |  |  |  |  |  |  |  |  |
| rs67124431 | Gastric cancer | 5 | 89851835 | C/T | 0.146 | 0.040 | 0.064 | 0.533 | 21.080 |
| rs974939 | Gastric cancer | 5 | 89632472 | G/T | 0.816 | 0.034 | 0.024 | 0.159 | 58.480 |
| rs7716979 | Gastric cancer | 5 | 89819560 | C/A | 0.369 | 0.023 | 0.018 | 0.210 | 141.665 |
| rs77236023 | Gastric cancer | 5 | 90061812 | T/C | 0.313 | 0.022 | 0.019 | 0.232 | 60.664 |
| rs250372 | Gastric cancer | 5 | 89785412 | T/G | 0.635 | 0.007 | 0.016 | 0.690 | 66.333 |
| rs12187771 | Gastric cancer | 5 | 89994983 | G/A | 0.202 | -0.001 | 0.017 | 0.958 | 31.848 |
| rs1474119 | Gastric cancer | 5 | 90002885 | A/G | 0.440 | -0.008 | 0.016 | 0.622 | 46.745 |
| rs62375060 | Gastric cancer | 5 | 89810208 | T/C | 0.118 | -0.013 | 0.023 | 0.587 | 26.250 |
| rs10942605 | Gastric cancer | 5 | 89999954 | C/T | 0.641 | -0.019 | 0.020 | 0.349 | 88.316 |
| rs140615294 | Gastric cancer | 5 | 89875683 | G/T | 0.016 | -0.113 | 0.175 | 0.519 | 20.634 |
| ETV7 |  |  |  |  |  |  |  |  |  |
| rs141475156 | Gastric cancer | 6 | 36472042 | C/T | 0.014 | 0.189 | 0.197 | 0.337 | 55.468 |
| rs111367622 | Gastric cancer | 6 | 36495769 | T/C | 0.052 | 0.183 | 0.101 | 0.071 | 204.602 |
| rs12201516 | Gastric cancer | 6 | 36389225 | G/A | 0.049 | 0.159 | 0.105 | 0.129 | 242.366 |
| rs112548021 | Gastric cancer | 6 | 36459233 | C/T | 0.043 | 0.158 | 0.116 | 0.173 | 150.077 |
| rs192902704 | Gastric cancer | 6 | 36494993 | G/A | 0.032 | 0.138 | 0.154 | 0.370 | 31.967 |
| rs145537395 | Gastric cancer | 6 | 36557789 | A/G | 0.013 | 0.120 | 0.194 | 0.535 | 115.287 |
| rs588496 | Gastric cancer | 6 | 36476047 | A/G | 0.979 | 0.117 | 0.159 | 0.461 | 170.305 |
| rs114892399 | Gastric cancer | 6 | 36446105 | A/G | 0.022 | 0.114 | 0.153 | 0.458 | 137.703 |
| rs148687731 | Gastric cancer | 6 | 36416549 | A/C | 0.007 | 0.108 | 0.204 | 0.594 | 77.303 |
| rs6457937 | Gastric cancer | 6 | 36654351 | C/T | 0.974 | 0.096 | 0.143 | 0.500 | 121.838 |
| rs72852324 | Gastric cancer | 6 | 36522766 | G/A | 0.015 | 0.079 | 0.182 | 0.663 | 57.590 |
| rs147922698 | Gastric cancer | 6 | 36580439 | T/C | 0.028 | 0.074 | 0.138 | 0.593 | 49.136 |
| rs114403297 | Gastric cancer | 6 | 36561376 | T/C | 0.012 | 0.068 | 0.151 | 0.653 | 41.981 |
| rs35071464 | Gastric cancer | 6 | 36514343 | A/G | 0.023 | 0.062 | 0.148 | 0.673 | 88.417 |
| rs72848551 | Gastric cancer | 6 | 36260529 | G/A | 0.065 | 0.062 | 0.024 | 0.009 | 22.424 |
| rs9394348 | Gastric cancer | 6 | 36373675 | T/C | 0.105 | 0.058 | 0.019 | 0.002 | 225.601 |
| rs9368936 | Gastric cancer | 6 | 36476767 | C/T | 0.105 | 0.057 | 0.019 | 0.003 | 258.532 |
| rs62403759 | Gastric cancer | 6 | 36380369 | A/G | 0.025 | 0.049 | 0.074 | 0.508 | 22.505 |
| rs147041201 | Gastric cancer | 6 | 36593876 | A/C | 0.022 | 0.047 | 0.153 | 0.760 | 33.584 |
| rs881647 | Gastric cancer | 6 | 36350814 | A/G | 0.567 | 0.046 | 0.016 | 0.004 | 3846.467 |
| rs75658816 | Gastric cancer | 6 | 36340647 | G/T | 0.035 | 0.044 | 0.124 | 0.722 | 31.395 |
| rs6928048 | Gastric cancer | 6 | 36345180 | A/G | 0.482 | 0.044 | 0.017 | 0.008 | 1473.018 |
| rs7758422 | Gastric cancer | 6 | 36344370 | T/C | 0.706 | 0.043 | 0.021 | 0.043 | 184.776 |
| rs115430384 | Gastric cancer | 6 | 36264472 | A/G | 0.038 | 0.042 | 0.118 | 0.722 | 21.388 |
| rs71569332 | Gastric cancer | 6 | 36347754 | C/T | 0.023 | 0.041 | 0.153 | 0.786 | 95.102 |
| rs11550973 | Gastric cancer | 6 | 36654964 | G/A | 0.017 | 0.040 | 0.181 | 0.824 | 24.704 |
| rs3176329 | Gastric cancer | 6 | 36647463 | G/T | 0.913 | 0.040 | 0.029 | 0.173 | 60.588 |
| rs3734339 | Gastric cancer | 6 | 36562982 | C/T | 0.143 | 0.035 | 0.022 | 0.100 | 48.165 |
| rs78323290 | Gastric cancer | 6 | 36297641 | A/G | 0.132 | 0.035 | 0.081 | 0.670 | 53.439 |
| rs187721802 | Gastric cancer | 6 | 36305387 | T/C | 0.016 | 0.034 | 0.182 | 0.854 | 21.505 |
| rs6906529 | Gastric cancer | 6 | 36360987 | G/A | 0.807 | 0.031 | 0.019 | 0.107 | 1152.172 |
| rs7758498 | Gastric cancer | 6 | 36344213 | A/G | 0.176 | 0.027 | 0.020 | 0.182 | 120.555 |
| rs114909292 | Gastric cancer | 6 | 36315002 | T/C | 0.027 | 0.019 | 0.139 | 0.890 | 34.521 |
| rs73730104 | Gastric cancer | 6 | 36617780 | C/T | 0.063 | 0.019 | 0.053 | 0.718 | 67.771 |
| rs17360657 | Gastric cancer | 6 | 36516027 | C/T | 0.034 | 0.019 | 0.123 | 0.879 | 127.130 |
| rs113994697 | Gastric cancer | 6 | 36457966 | C/T | 0.010 | 0.018 | 0.232 | 0.937 | 20.539 |
| rs12199580 | Gastric cancer | 6 | 36270130 | A/C | 0.380 | 0.017 | 0.018 | 0.346 | 59.267 |
| rs13202984 | Gastric cancer | 6 | 36581149 | G/A | 0.280 | 0.017 | 0.046 | 0.711 | 1139.064 |
| rs117183149 | Gastric cancer | 6 | 36486333 | C/A | 0.014 | 0.017 | 0.199 | 0.933 | 47.363 |
| rs116616466 | Gastric cancer | 6 | 36429277 | G/A | 0.036 | 0.013 | 0.127 | 0.918 | 83.331 |
| rs10947603 | Gastric cancer | 6 | 36344292 | C/T | 0.512 | 0.012 | 0.048 | 0.806 | 72.375 |
| rs146313687 | Gastric cancer | 6 | 36579514 | T/C | 0.052 | 0.011 | 0.101 | 0.917 | 161.145 |
| rs13208903 | Gastric cancer | 6 | 36268998 | A/G | 0.722 | 0.008 | 0.018 | 0.634 | 22.627 |
| rs116713091 | Gastric cancer | 6 | 36360229 | G/T | 0.046 | 0.008 | 0.107 | 0.942 | 130.979 |
| rs114910336 | Gastric cancer | 6 | 36386070 | C/T | 0.052 | 0.007 | 0.101 | 0.942 | 165.152 |
| rs12201851 | Gastric cancer | 6 | 36271845 | A/C | 0.120 | 0.006 | 0.032 | 0.843 | 41.111 |
| rs7382195 | Gastric cancer | 6 | 36621000 | A/G | 0.068 | 0.002 | 0.021 | 0.910 | 28.585 |
| rs113615426 | Gastric cancer | 6 | 36510596 | A/G | 0.058 | 0.002 | 0.117 | 0.986 | 140.752 |
| rs12664053 | Gastric cancer | 6 | 36586472 | T/C | 0.066 | 0.001 | 0.023 | 0.950 | 27.783 |
| rs2071797 | Gastric cancer | 6 | 36343333 | T/C | 0.128 | 0.000 | 0.019 | 0.990 | 58.818 |
| rs6915170 | Gastric cancer | 6 | 36621039 | G/A | 0.280 | 0.000 | 0.016 | 0.992 | 238.730 |
| rs78911376 | Gastric cancer | 6 | 36262940 | A/G | 0.087 | -0.002 | 0.032 | 0.961 | 28.949 |
| rs1801270 | Gastric cancer | 6 | 36651971 | A/C | 0.169 | -0.003 | 0.017 | 0.843 | 22.730 |
| rs4236051 | Gastric cancer | 6 | 36469821 | T/C | 0.043 | -0.003 | 0.024 | 0.891 | 43.821 |
| rs61212666 | Gastric cancer | 6 | 36344634 | A/C | 0.120 | -0.004 | 0.023 | 0.848 | 284.472 |
| rs6902994 | Gastric cancer | 6 | 36615675 | C/T | 0.877 | -0.006 | 0.024 | 0.814 | 27.749 |
| rs148260186 | Gastric cancer | 6 | 36641993 | A/C | 0.027 | -0.008 | 0.138 | 0.952 | 36.825 |
| rs143701697 | Gastric cancer | 6 | 36543179 | G/A | 0.047 | -0.010 | 0.110 | 0.930 | 179.606 |
| rs79601514 | Gastric cancer | 6 | 36361176 | T/C | 0.130 | -0.011 | 0.024 | 0.654 | 31.711 |
| rs3176321 | Gastric cancer | 6 | 36646807 | T/C | 0.019 | -0.012 | 0.168 | 0.943 | 38.122 |
| rs12202339 | Gastric cancer | 6 | 36289610 | G/A | 0.611 | -0.015 | 0.016 | 0.341 | 25.275 |
| rs11754995 | Gastric cancer | 6 | 36612323 | A/G | 0.290 | -0.019 | 0.021 | 0.362 | 472.411 |
| rs150507082 | Gastric cancer | 6 | 36351883 | T/C | 0.024 | -0.020 | 0.145 | 0.892 | 67.275 |
| rs11751412 | Gastric cancer | 6 | 36281463 | G/T | 0.341 | -0.020 | 0.016 | 0.205 | 73.600 |
| rs73417359 | Gastric cancer | 6 | 36518344 | C/T | 0.136 | -0.020 | 0.021 | 0.345 | 435.116 |
| rs9394361 | Gastric cancer | 6 | 36551370 | C/A | 0.302 | -0.021 | 0.016 | 0.195 | 1200.249 |
| rs1744657 | Gastric cancer | 6 | 36527630 | C/T | 0.048 | -0.022 | 0.065 | 0.738 | 249.944 |
| rs7776298 | Gastric cancer | 6 | 36548225 | G/A | 0.079 | -0.024 | 0.022 | 0.287 | 125.028 |
| rs111701607 | Gastric cancer | 6 | 36333168 | G/A | 0.062 | -0.025 | 0.098 | 0.798 | 89.365 |
| rs28525524 | Gastric cancer | 6 | 36289655 | C/A | 0.167 | -0.026 | 0.017 | 0.123 | 43.815 |
| rs12173412 | Gastric cancer | 6 | 36230078 | T/G | 0.042 | -0.026 | 0.037 | 0.482 | 25.010 |
| rs13196885 | Gastric cancer | 6 | 36632688 | T/C | 0.454 | -0.029 | 0.019 | 0.121 | 170.898 |
| rs111414867 | Gastric cancer | 6 | 36589903 | T/C | 0.038 | -0.032 | 0.117 | 0.784 | 89.370 |
| rs74368467 | Gastric cancer | 6 | 36493120 | T/C | 0.024 | -0.042 | 0.144 | 0.769 | 63.237 |
| rs9470289 | Gastric cancer | 6 | 36451115 | C/A | 0.272 | -0.044 | 0.016 | 0.007 | 1116.582 |
| rs12203115 | Gastric cancer | 6 | 36490733 | G/A | 0.289 | -0.045 | 0.050 | 0.365 | 1240.201 |
| rs7740345 | Gastric cancer | 6 | 36344329 | C/T | 0.869 | -0.045 | 0.020 | 0.026 | 57.318 |
| rs79386604 | Gastric cancer | 6 | 36325325 | T/C | 0.061 | -0.047 | 0.094 | 0.616 | 25.795 |
| rs10947605 | Gastric cancer | 6 | 36374216 | C/T | 0.045 | -0.048 | 0.113 | 0.672 | 226.461 |
| rs144894654 | Gastric cancer | 6 | 36405861 | C/T | 0.128 | -0.048 | 0.030 | 0.112 | 670.455 |
| rs186368520 | Gastric cancer | 6 | 36405329 | T/C | 0.028 | -0.051 | 0.164 | 0.758 | 137.068 |
| rs12190328 | Gastric cancer | 6 | 36330513 | T/C | 0.054 | -0.052 | 0.065 | 0.429 | 23.443 |
| rs143000423 | Gastric cancer | 6 | 36591128 | A/C | 0.017 | -0.052 | 0.177 | 0.768 | 48.800 |
| rs35134930 | Gastric cancer | 6 | 36298690 | A/G | 0.034 | -0.055 | 0.130 | 0.672 | 28.136 |
| rs62403700 | Gastric cancer | 6 | 36292645 | G/T | 0.043 | -0.084 | 0.111 | 0.448 | 55.952 |
| rs79133352 | Gastric cancer | 6 | 36421731 | A/G | 0.035 | -0.086 | 0.075 | 0.249 | 233.347 |
| rs183369893 | Gastric cancer | 6 | 36535770 | G/A | 0.024 | -0.091 | 0.155 | 0.555 | 35.259 |
| rs76847127 | Gastric cancer | 6 | 36443643 | G/A | 0.022 | -0.100 | 0.123 | 0.415 | 77.602 |
| rs138694427 | Gastric cancer | 6 | 36549628 | A/G | 0.015 | -0.109 | 0.180 | 0.545 | 41.627 |
| rs76173736 | Gastric cancer | 6 | 36523873 | T/C | 0.019 | -0.123 | 0.163 | 0.450 | 40.653 |
| rs45613131 | Gastric cancer | 6 | 36284833 | A/G | 0.016 | -0.123 | 0.178 | 0.488 | 19.827 |
| rs185562809 | Gastric cancer | 6 | 36483955 | A/C | 0.017 | -0.128 | 0.169 | 0.449 | 42.069 |
| rs6937326 | Gastric cancer | 6 | 36364707 | A/G | 0.048 | -0.140 | 0.048 | 0.003 | 243.775 |
| rs190109922 | Gastric cancer | 6 | 36435303 | T/C | 0.011 | -0.146 | 0.227 | 0.520 | 36.707 |
| rs79511725 | Gastric cancer | 6 | 36601087 | G/A | 0.049 | -0.182 | 0.104 | 0.082 | 161.400 |
| UACA |  |  |  |  |  |  |  |  |  |
| rs62019067 | Gastric cancer | 15 | 71126029 | A/C | 0.020 | 0.121 | 0.157 | 0.441 | 22.728 |
| rs4777306 | Gastric cancer | 15 | 71055860 | G/T | 0.205 | 0.061 | 0.055 | 0.269 | 27.430 |
| rs77151411 | Gastric cancer | 15 | 71237035 | T/C | 0.027 | 0.044 | 0.135 | 0.746 | 55.142 |
| rs8029150 | Gastric cancer | 15 | 70937749 | T/C | 0.441 | 0.026 | 0.018 | 0.146 | 50.360 |
| rs920532 | Gastric cancer | 15 | 71231505 | C/T | 0.521 | 0.008 | 0.016 | 0.617 | 75.511 |
| rs11072218 | Gastric cancer | 15 | 71110504 | A/G | 0.473 | 0.004 | 0.016 | 0.820 | 145.552 |
| rs34298001 | Gastric cancer | 15 | 70897505 | A/G | 0.054 | -0.010 | 0.043 | 0.815 | 203.562 |
| rs112166746 | Gastric cancer | 15 | 71346553 | G/A | 0.050 | -0.019 | 0.101 | 0.853 | 93.886 |
| rs111959648 | Gastric cancer | 15 | 71229606 | A/G | 0.050 | -0.020 | 0.101 | 0.846 | 93.751 |
| rs28497234 | Gastric cancer | 15 | 71095723 | C/T | 0.063 | -0.020 | 0.093 | 0.826 | 87.374 |
| rs720251 | Gastric cancer | 15 | 70938445 | T/C | 0.081 | -0.053 | 0.039 | 0.172 | 382.788 |
| rs12905447 | Gastric cancer | 15 | 70906951 | G/A | 0.183 | -0.065 | 0.070 | 0.350 | 141.339 |
| rs62001713 | Gastric cancer | 15 | 70721473 | G/A | 0.034 | -0.091 | 0.123 | 0.463 | 26.279 |
| rs62019092 | Gastric cancer | 15 | 71188935 | A/G | 0.030 | -0.127 | 0.130 | 0.327 | 64.521 |
| rs62016877 | Gastric cancer | 15 | 70902510 | A/G | 0.021 | -0.183 | 0.122 | 0.135 | 120.815 |
| rs76166163 | Gastric cancer | 15 | 70767674 | T/C | 0.036 | -0.183 | 0.122 | 0.132 | 23.735 |
| rs147056668 | Gastric cancer | 15 | 70884693 | G/T | 0.056 | -0.200 | 0.099 | 0.043 | 60.033 |
| rs62016954 | Gastric cancer | 15 | 71043451 | C/T | 0.017 | -0.200 | 0.174 | 0.249 | 115.995 |
| rs62019063 | Gastric cancer | 15 | 71077752 | A/G | 0.029 | -0.203 | 0.132 | 0.125 | 101.202 |
| rs74488675 | Gastric cancer | 15 | 70649109 | A/G | 0.030 | -0.208 | 0.130 | 0.112 | 21.071 |
| rs78588389 | Gastric cancer | 15 | 70992028 | C/T | 0.038 | -0.230 | 0.120 | 0.055 | 93.399 |
| rs77986018 | Gastric cancer | 15 | 71258179 | C/T | 0.019 | -0.239 | 0.286 | 0.405 | 33.834 |
| TJAP1 |  |  |  |  |  |  |  |  |  |
| rs3734683 | Gastric cancer | 6 | 43316023 | G/A | 0.087 | -0.017 | 0.033 | 0.613 | 19.859 |
| rs113154221 | Gastric cancer | 6 | 43284865 | A/G | 0.019 | -0.123 | 0.115 | 0.285 | 25.342 |
| rs1111786 | Gastric cancer | 6 | 43496869 | A/G | 0.021 | -0.212 | 0.155 | 0.172 | 44.439 |
| rs11967462 | Gastric cancer | 6 | 43622925 | G/A | 0.022 | -0.222 | 0.154 | 0.150 | 44.656 |
| RAB3GAP2 |  |  |  |  |  |  |  |  |  |
| rs147643571 | Gastric cancer | 1 | 220478116 | C/T | 0.016 | 0.573 | 0.179 | 0.001 | 33.479 |
| rs113585940 | Gastric cancer | 1 | 220460873 | C/A | 0.010 | 0.403 | 0.231 | 0.080 | 47.687 |
| rs74938639 | Gastric cancer | 1 | 220371869 | T/C | 0.115 | 0.078 | 0.070 | 0.270 | 49.753 |
| rs76523895 | Gastric cancer | 1 | 220187143 | A/G | 0.113 | 0.053 | 0.072 | 0.463 | 41.200 |
| rs1618308 | Gastric cancer | 1 | 220114373 | C/T | 0.479 | 0.036 | 0.016 | 0.025 | 93.315 |
| rs75426550 | Gastric cancer | 1 | 220286984 | T/G | 0.087 | 0.032 | 0.022 | 0.155 | 56.858 |
| rs113735504 | Gastric cancer | 1 | 220473090 | C/T | 0.116 | 0.025 | 0.030 | 0.419 | 27.490 |
| rs34947552 | Gastric cancer | 1 | 220671961 | T/C | 0.082 | 0.022 | 0.082 | 0.788 | 22.600 |
| rs2808020 | Gastric cancer | 1 | 220455062 | G/T | 0.799 | 0.014 | 0.021 | 0.515 | 51.621 |
| rs6673738 | Gastric cancer | 1 | 220499020 | A/G | 0.234 | 0.010 | 0.021 | 0.623 | 104.718 |
| rs11584853 | Gastric cancer | 1 | 220579458 | C/T | 0.553 | 0.005 | 0.016 | 0.749 | 47.983 |
| rs4379680 | Gastric cancer | 1 | 220174740 | T/C | 0.442 | 0.003 | 0.018 | 0.877 | 238.508 |
| rs11118496 | Gastric cancer | 1 | 220286982 | T/G | 0.675 | -0.002 | 0.018 | 0.922 | 27.393 |
| rs146567439 | Gastric cancer | 1 | 220537919 | A/G | 0.058 | -0.007 | 0.098 | 0.947 | 88.669 |
| rs2808018 | Gastric cancer | 1 | 220444675 | T/G | 0.356 | -0.019 | 0.016 | 0.246 | 436.041 |
| rs2577130 | Gastric cancer | 1 | 220343816 | C/T | 0.439 | -0.022 | 0.017 | 0.180 | 509.581 |
| rs2647434 | Gastric cancer | 1 | 220160433 | G/A | 0.845 | -0.025 | 0.028 | 0.363 | 123.128 |
| rs17563262 | Gastric cancer | 1 | 220240862 | G/A | 0.178 | -0.028 | 0.021 | 0.199 | 128.322 |
| rs75315957 | Gastric cancer | 1 | 220118365 | T/G | 0.038 | -0.033 | 0.027 | 0.229 | 52.985 |
| rs139351391 | Gastric cancer | 1 | 220410896 | A/G | 0.020 | -0.036 | 0.045 | 0.428 | 105.693 |
| rs114000129 | Gastric cancer | 1 | 220286963 | T/C | 0.021 | -0.040 | 0.043 | 0.349 | 87.350 |
| rs12734813 | Gastric cancer | 1 | 220229789 | A/G | 0.070 | -0.044 | 0.087 | 0.618 | 29.391 |
| rs75318434 | Gastric cancer | 1 | 220725322 | C/A | 0.040 | -0.044 | 0.113 | 0.699 | 20.112 |
| rs2577128 | Gastric cancer | 1 | 220348831 | C/T | 0.893 | -0.044 | 0.073 | 0.543 | 25.085 |
| rs12732754 | Gastric cancer | 1 | 220482432 | A/G | 0.326 | -0.059 | 0.029 | 0.037 | 142.110 |
| rs116750137 | Gastric cancer | 1 | 220514491 | A/G | 0.019 | -0.076 | 0.104 | 0.461 | 80.853 |
| rs35775533 | Gastric cancer | 1 | 220307676 | G/A | 0.079 | -0.111 | 0.085 | 0.191 | 31.601 |
| rs34535491 | Gastric cancer | 1 | 220453309 | T/C | 0.071 | -0.116 | 0.089 | 0.193 | 19.544 |
| rs34080032 | Gastric cancer | 1 | 220103644 | C/T | 0.105 | -0.123 | 0.073 | 0.092 | 48.518 |
| rs192403233 | Gastric cancer | 1 | 220640633 | A/G | 0.020 | -0.123 | 0.164 | 0.451 | 40.284 |
| rs115125755 | Gastric cancer | 1 | 220495129 | C/T | 0.019 | -0.124 | 0.168 | 0.460 | 66.787 |
| rs12135468 | Gastric cancer | 1 | 220331846 | A/G | 0.078 | -0.126 | 0.084 | 0.132 | 38.379 |
| rs116319760 | Gastric cancer | 1 | 220320623 | T/C | 0.018 | -0.184 | 0.172 | 0.284 | 50.296 |
| rs115214196 | Gastric cancer | 1 | 220254306 | A/G | 0.018 | -0.325 | 0.299 | 0.277 | 33.428 |
| LILRB3 |  |  |  |  |  |  |  |  |  |
| rs148442614 | Gastric cancer | 19 | 54699967 | T/C | 0.019 | 0.531 | 0.306 | 0.083 | 70.939 |
| rs75814910 | Gastric cancer | 19 | 54812494 | A/C | 0.070 | 0.164 | 0.088 | 0.062 | 46.019 |
| rs79085660 | Gastric cancer | 19 | 54565435 | A/G | 0.022 | 0.151 | 0.155 | 0.332 | 21.728 |
| rs80047999 | Gastric cancer | 19 | 54656521 | T/C | 0.036 | 0.151 | 0.120 | 0.208 | 51.322 |
| rs76905990 | Gastric cancer | 19 | 54808431 | A/G | 0.086 | 0.100 | 0.080 | 0.212 | 25.988 |
| rs111256210 | Gastric cancer | 19 | 54679739 | C/T | 0.084 | 0.095 | 0.083 | 0.252 | 21.223 |
| rs3848620 | Gastric cancer | 19 | 54787418 | T/C | 0.358 | 0.069 | 0.047 | 0.137 | 61.841 |
| rs17207321 | Gastric cancer | 19 | 54709443 | G/T | 0.225 | 0.051 | 0.031 | 0.095 | 369.564 |
| rs35573925 | Gastric cancer | 19 | 54752836 | T/C | 0.070 | 0.043 | 0.107 | 0.685 | 19.623 |
| rs2361796 | Gastric cancer | 19 | 54754385 | G/T | 0.316 | 0.025 | 0.049 | 0.608 | 22.224 |
| rs397600 | Gastric cancer | 19 | 54791788 | G/A | 0.519 | 0.017 | 0.026 | 0.512 | 53.153 |
| rs116858035 | Gastric cancer | 19 | 54605597 | T/C | 0.053 | 0.016 | 0.101 | 0.874 | 19.547 |
| rs11668526 | Gastric cancer | 19 | 54749060 | T/C | 0.512 | 0.012 | 0.019 | 0.520 | 32.016 |
| rs17239607 | Gastric cancer | 19 | 54743217 | T/C | 0.279 | 0.012 | 0.052 | 0.820 | 46.722 |
| rs3810232 | Gastric cancer | 19 | 54704760 | C/A | 0.409 | 0.008 | 0.016 | 0.600 | 25.654 |
| rs1802057 | Gastric cancer | 19 | 54754589 | C/T | 0.017 | 0.005 | 0.078 | 0.952 | 22.108 |
| rs7260261 | Gastric cancer | 19 | 54785779 | T/C | 0.205 | 0.003 | 0.055 | 0.955 | 42.165 |
| rs112312277 | Gastric cancer | 19 | 54706844 | T/G | 0.057 | 0.002 | 0.099 | 0.983 | 276.650 |
| rs4341862 | Gastric cancer | 19 | 54736299 | A/C | 0.267 | -0.012 | 0.066 | 0.859 | 48.961 |
| rs1645784 | Gastric cancer | 19 | 54827040 | C/A | 0.135 | -0.017 | 0.068 | 0.800 | 31.190 |
| rs149200093 | Gastric cancer | 19 | 54753823 | G/A | 0.095 | -0.020 | 0.076 | 0.792 | 79.891 |
| rs11671231 | Gastric cancer | 19 | 54822205 | T/C | 0.013 | -0.024 | 0.375 | 0.949 | 21.166 |
| rs635711 | Gastric cancer | 19 | 54748760 | T/G | 0.505 | -0.026 | 0.018 | 0.160 | 161.638 |
| rs1761462 | Gastric cancer | 19 | 54824667 | C/T | 0.329 | -0.030 | 0.048 | 0.536 | 32.850 |
| rs606851 | Gastric cancer | 19 | 54750633 | A/G | 0.401 | -0.030 | 0.019 | 0.110 | 27.188 |
| rs389096 | Gastric cancer | 19 | 54764322 | G/A | 0.454 | -0.033 | 0.020 | 0.099 | 46.123 |
| rs404035 | Gastric cancer | 19 | 54752718 | A/C | 0.332 | -0.050 | 0.048 | 0.294 | 26.100 |
| rs2483832 | Gastric cancer | 19 | 54757676 | C/T | 0.841 | -0.059 | 0.041 | 0.154 | 104.565 |
| rs148119235 | Gastric cancer | 19 | 54729916 | A/G | 0.028 | -0.107 | 0.175 | 0.542 | 35.903 |
| rs2668839 | Gastric cancer | 19 | 54657815 | G/A | 0.055 | -0.406 | 0.178 | 0.023 | 27.166 |
| PARD6G |  |  |  |  |  |  |  |  |  |
| rs78582178 | Gastric cancer | 18 | 77963730 | C/T | 0.020 | 0.190 | 0.161 | 0.240 | 27.855 |
| rs77279912 | Gastric cancer | 18 | 77845071 | A/G | 0.020 | 0.180 | 0.160 | 0.260 | 26.007 |
| rs111257097 | Gastric cancer | 18 | 77883521 | A/G | 0.042 | 0.176 | 0.135 | 0.192 | 20.398 |
| rs7245133 | Gastric cancer | 18 | 77733860 | T/C | 0.020 | 0.162 | 0.158 | 0.306 | 27.892 |
| rs36083942 | Gastric cancer | 18 | 77894470 | G/A | 0.019 | 0.114 | 0.169 | 0.498 | 24.228 |
| rs117113493 | Gastric cancer | 18 | 77766460 | T/C | 0.018 | 0.105 | 0.171 | 0.541 | 24.405 |
| rs60861849 | Gastric cancer | 18 | 77819828 | T/C | 0.287 | 0.040 | 0.024 | 0.098 | 89.936 |
| rs7227729 | Gastric cancer | 18 | 77825934 | T/C | 0.526 | 0.039 | 0.021 | 0.066 | 49.798 |
| rs11665580 | Gastric cancer | 18 | 77937261 | C/T | 0.494 | 0.036 | 0.022 | 0.105 | 145.765 |
| rs9967045 | Gastric cancer | 18 | 77818987 | G/T | 0.647 | 0.033 | 0.016 | 0.042 | 135.713 |
| rs56350247 | Gastric cancer | 18 | 77997191 | C/T | 0.447 | 0.023 | 0.020 | 0.259 | 50.815 |
| rs11663146 | Gastric cancer | 18 | 77972066 | T/C | 0.224 | 0.009 | 0.055 | 0.867 | 46.524 |
| rs62101202 | Gastric cancer | 18 | 77709709 | C/T | 0.132 | -0.003 | 0.025 | 0.894 | 44.398 |
| rs4331399 | Gastric cancer | 18 | 77826246 | G/A | 0.939 | -0.005 | 0.030 | 0.882 | 22.470 |
| rs4081877 | Gastric cancer | 18 | 77721125 | G/A | 0.375 | -0.005 | 0.020 | 0.790 | 25.090 |
| rs72976343 | Gastric cancer | 18 | 77865159 | A/C | 0.161 | -0.008 | 0.061 | 0.899 | 34.242 |
| rs62101566 | Gastric cancer | 18 | 77923958 | C/T | 0.511 | -0.019 | 0.016 | 0.237 | 37.939 |
| rs12953470 | Gastric cancer | 18 | 77938205 | T/C | 0.142 | -0.020 | 0.023 | 0.399 | 41.477 |
| rs3865386 | Gastric cancer | 18 | 77771983 | A/C | 0.181 | -0.026 | 0.017 | 0.119 | 53.478 |
| rs12454507 | Gastric cancer | 18 | 77825981 | G/A | 0.393 | -0.027 | 0.018 | 0.139 | 27.128 |
| rs62101563 | Gastric cancer | 18 | 77914316 | T/C | 0.204 | -0.033 | 0.017 | 0.043 | 69.446 |
| rs12456851 | Gastric cancer | 18 | 78014582 | A/C | 0.233 | -0.034 | 0.018 | 0.050 | 54.845 |
| NXF1 |  |  |  |  |  |  |  |  |  |
| rs146837093 | Gastric cancer | 11 | 62764257 | C/T | 0.012 | 0.265 | 0.206 | 0.197 | 29.684 |
| rs56023117 | Gastric cancer | 11 | 62706182 | G/A | 0.275 | 0.108 | 0.050 | 0.029 | 75.438 |
| rs117926699 | Gastric cancer | 11 | 62515792 | C/T | 0.047 | 0.099 | 0.105 | 0.347 | 20.937 |
| rs143645326 | Gastric cancer | 11 | 62575783 | C/A | 0.017 | 0.090 | 0.122 | 0.460 | 34.513 |
| rs143203448 | Gastric cancer | 11 | 62513163 | A/C | 0.031 | 0.087 | 0.159 | 0.584 | 39.525 |
| rs1286672 | Gastric cancer | 11 | 62514001 | G/T | 0.691 | 0.084 | 0.059 | 0.154 | 61.626 |
| rs11600264 | Gastric cancer | 11 | 62420552 | C/T | 0.021 | 0.080 | 0.157 | 0.611 | 22.314 |
| rs113554344 | Gastric cancer | 11 | 62610758 | A/C | 0.053 | 0.070 | 0.102 | 0.493 | 37.519 |
| rs3888173 | Gastric cancer | 11 | 62346131 | T/C | 0.110 | 0.059 | 0.071 | 0.405 | 23.985 |
| rs2845672 | Gastric cancer | 11 | 62496923 | C/T | 0.212 | 0.037 | 0.055 | 0.501 | 78.236 |
| rs527481 | Gastric cancer | 11 | 62603912 | A/G | 0.247 | 0.027 | 0.021 | 0.194 | 150.292 |
| rs4693 | Gastric cancer | 11 | 62564009 | C/T | 0.609 | 0.026 | 0.016 | 0.106 | 627.022 |
| rs2187383 | Gastric cancer | 11 | 62775898 | C/A | 0.641 | 0.018 | 0.018 | 0.318 | 97.008 |
| rs17664979 | Gastric cancer | 11 | 62303822 | T/C | 0.063 | 0.016 | 0.091 | 0.861 | 36.050 |
| rs67754977 | Gastric cancer | 11 | 62781822 | G/A | 0.163 | 0.015 | 0.021 | 0.454 | 33.652 |
| rs7105855 | Gastric cancer | 11 | 62564413 | T/C | 0.282 | 0.013 | 0.018 | 0.478 | 195.759 |
| rs17157628 | Gastric cancer | 11 | 62675687 | A/G | 0.093 | 0.012 | 0.030 | 0.685 | 97.911 |
| rs11231264 | Gastric cancer | 11 | 62665416 | A/G | 0.271 | 0.010 | 0.020 | 0.600 | 155.891 |
| rs72927301 | Gastric cancer | 11 | 62441553 | T/C | 0.103 | -0.001 | 0.074 | 0.989 | 72.703 |
| rs7942154 | Gastric cancer | 11 | 62337163 | A/G | 0.213 | -0.002 | 0.032 | 0.961 | 26.162 |
| rs12804436 | Gastric cancer | 11 | 62664486 | T/C | 0.273 | -0.007 | 0.017 | 0.691 | 275.566 |
| rs11231168 | Gastric cancer | 11 | 62401620 | T/C | 0.397 | -0.013 | 0.018 | 0.478 | 95.857 |
| rs111438108 | Gastric cancer | 11 | 62730553 | T/C | 0.156 | -0.015 | 0.020 | 0.471 | 38.598 |
| rs12279355 | Gastric cancer | 11 | 62663508 | A/G | 0.178 | -0.015 | 0.020 | 0.441 | 47.840 |
| rs12272560 | Gastric cancer | 11 | 62684777 | A/G | 0.031 | -0.017 | 0.086 | 0.847 | 33.429 |
| rs1942499 | Gastric cancer | 11 | 62683525 | G/A | 0.334 | -0.021 | 0.017 | 0.217 | 101.660 |
| rs11231221 | Gastric cancer | 11 | 62559501 | T/C | 0.102 | -0.027 | 0.024 | 0.246 | 77.830 |
| rs2067479 | Gastric cancer | 11 | 62677352 | A/G | 0.059 | -0.028 | 0.095 | 0.770 | 22.875 |
| rs12794763 | Gastric cancer | 11 | 62625512 | G/T | 0.181 | -0.029 | 0.019 | 0.120 | 185.504 |
| rs486903 | Gastric cancer | 11 | 62293948 | A/G | 0.773 | -0.043 | 0.065 | 0.506 | 24.123 |
| rs151337032 | Gastric cancer | 11 | 62618286 | T/C | 0.026 | -0.059 | 0.140 | 0.672 | 35.825 |
| rs74385851 | Gastric cancer | 11 | 62685926 | T/C | 0.019 | -0.082 | 0.135 | 0.544 | 22.454 |
| rs7935682 | Gastric cancer | 11 | 62541627 | T/C | 0.036 | -0.082 | 0.123 | 0.504 | 26.983 |
| rs12803988 | Gastric cancer | 11 | 62539760 | A/G | 0.124 | -0.083 | 0.068 | 0.220 | 42.323 |
| rs138102068 | Gastric cancer | 11 | 62498613 | A/G | 0.033 | -0.102 | 0.128 | 0.425 | 22.969 |
| rs117762989 | Gastric cancer | 11 | 62603644 | T/C | 0.034 | -0.104 | 0.125 | 0.405 | 19.935 |
| rs76980804 | Gastric cancer | 11 | 62406056 | G/A | 0.044 | -0.146 | 0.132 | 0.269 | 30.558 |
| rs148248716 | Gastric cancer | 11 | 62451457 | T/C | 0.021 | -0.166 | 0.155 | 0.285 | 21.308 |
| rs184333012 | Gastric cancer | 11 | 62385607 | T/C | 0.019 | -0.217 | 0.209 | 0.301 | 22.008 |
| rs11604528 | Gastric cancer | 11 | 62435668 | A/G | 0.044 | -0.230 | 0.111 | 0.037 | 20.364 |
| rs79678172 | Gastric cancer | 11 | 62609779 | A/G | 0.026 | -0.257 | 0.140 | 0.066 | 47.968 |
| rs117162358 | Gastric cancer | 11 | 62344743 | T/C | 0.018 | -0.295 | 0.166 | 0.076 | 20.735 |
| HIF1A |  |  |  |  |  |  |  |  |  |
| rs143228053 | Gastric cancer | 14 | 61943748 | C/T | 0.022 | 0.144 | 0.157 | 0.360 | 83.241 |
| rs77899560 | Gastric cancer | 14 | 61944422 | G/A | 0.016 | 0.076 | 0.174 | 0.663 | 56.600 |
| rs710046 | Gastric cancer | 14 | 62119993 | G/A | 0.854 | 0.025 | 0.020 | 0.216 | 132.849 |
| rs55701546 | Gastric cancer | 14 | 62065991 | A/G | 0.158 | 0.025 | 0.018 | 0.180 | 25.235 |
| rs3783765 | Gastric cancer | 14 | 61968118 | T/C | 0.844 | 0.020 | 0.021 | 0.348 | 164.911 |
| rs912617 | Gastric cancer | 14 | 61933536 | T/G | 0.856 | 0.019 | 0.017 | 0.268 | 22.102 |
| rs7159288 | Gastric cancer | 14 | 61980098 | G/A | 0.137 | 0.015 | 0.017 | 0.380 | 20.218 |
| rs1957898 | Gastric cancer | 14 | 61953406 | C/T | 0.830 | 0.008 | 0.071 | 0.915 | 32.729 |
| rs12147178 | Gastric cancer | 14 | 61985758 | G/T | 0.108 | 0.004 | 0.022 | 0.854 | 27.760 |
| rs117865147 | Gastric cancer | 14 | 61937591 | A/G | 0.026 | -0.005 | 0.142 | 0.970 | 23.657 |
| rs7156083 | Gastric cancer | 14 | 62117489 | T/C | 0.119 | -0.013 | 0.018 | 0.463 | 26.955 |
| rs2255998 | Gastric cancer | 14 | 62051682 | A/G | 0.952 | -0.015 | 0.026 | 0.553 | 20.531 |
| rs2301106 | Gastric cancer | 14 | 62166563 | C/T | 0.091 | -0.015 | 0.034 | 0.648 | 22.343 |
| rs4899050 | Gastric cancer | 14 | 61943886 | G/A | 0.224 | -0.027 | 0.020 | 0.173 | 48.695 |
| rs17834526 | Gastric cancer | 14 | 61947466 | T/C | 0.094 | -0.040 | 0.083 | 0.628 | 25.839 |
| rs80137372 | Gastric cancer | 14 | 61991504 | A/G | 0.044 | -0.052 | 0.046 | 0.256 | 26.891 |
| rs138834791 | Gastric cancer | 14 | 62138875 | A/G | 0.014 | -0.118 | 0.200 | 0.555 | 77.602 |
| rs111522409 | Gastric cancer | 14 | 62150735 | G/A | 0.035 | -0.173 | 0.123 | 0.161 | 250.801 |
| rs76629437 | Gastric cancer | 14 | 62026895 | A/G | 0.020 | -0.196 | 0.159 | 0.219 | 32.490 |
| rs72716792 | Gastric cancer | 14 | 62333094 | G/A | 0.026 | -0.232 | 0.144 | 0.107 | 52.152 |
| rs117934878 | Gastric cancer | 14 | 62226682 | T/C | 0.020 | -0.291 | 0.164 | 0.075 | 65.054 |
| rs191551606 | Gastric cancer | 14 | 62191470 | T/G | 0.006 | -0.367 | 0.298 | 0.217 | 65.513 |
| SP100 |  |  |  |  |  |  |  |  |  |
| rs4368317 | Gastric cancer | 2 | 231268938 | T/G | 0.063 | 0.068 | 0.080 | 0.399 | 29.070 |
| rs111631211 | Gastric cancer | 2 | 231368049 | T/C | 0.090 | 0.061 | 0.080 | 0.446 | 83.172 |
| rs76866282 | Gastric cancer | 2 | 231390132 | G/A | 0.046 | 0.043 | 0.108 | 0.690 | 41.836 |
| rs11692640 | Gastric cancer | 2 | 231326188 | G/A | 0.178 | 0.034 | 0.022 | 0.134 | 72.539 |
| rs11689260 | Gastric cancer | 2 | 231235886 | T/C | 0.532 | 0.023 | 0.018 | 0.215 | 21.337 |
| rs1649877 | Gastric cancer | 2 | 231334010 | T/C | 0.241 | 0.020 | 0.024 | 0.417 | 84.213 |
| rs62193433 | Gastric cancer | 2 | 231377364 | C/T | 0.653 | 0.010 | 0.016 | 0.516 | 181.324 |
| rs12618382 | Gastric cancer | 2 | 231517205 | G/A | 0.187 | 0.005 | 0.024 | 0.835 | 28.733 |
| rs111852601 | Gastric cancer | 2 | 231294187 | A/G | 0.044 | 0.004 | 0.108 | 0.972 | 23.609 |
| rs9678598 | Gastric cancer | 2 | 231346602 | G/A | 0.145 | 0.003 | 0.023 | 0.897 | 22.024 |
| rs11692527 | Gastric cancer | 2 | 231268163 | T/C | 0.246 | -0.002 | 0.028 | 0.958 | 44.376 |
| rs74422832 | Gastric cancer | 2 | 231439938 | A/G | 0.174 | -0.003 | 0.017 | 0.861 | 26.994 |
| rs4302207 | Gastric cancer | 2 | 231413943 | A/C | 0.438 | -0.007 | 0.016 | 0.690 | 29.922 |
| rs12465858 | Gastric cancer | 2 | 231274035 | C/T | 0.313 | -0.007 | 0.019 | 0.719 | 22.241 |
| rs62195480 | Gastric cancer | 2 | 231420942 | G/T | 0.196 | -0.011 | 0.041 | 0.781 | 22.123 |
| rs16827319 | Gastric cancer | 2 | 231375974 | A/G | 0.084 | -0.022 | 0.028 | 0.417 | 204.724 |
| rs1649915 | Gastric cancer | 2 | 231426058 | A/C | 0.192 | -0.025 | 0.021 | 0.224 | 21.392 |
| rs78181435 | Gastric cancer | 2 | 231459666 | T/C | 0.102 | -0.026 | 0.074 | 0.729 | 35.083 |
| rs16827285 | Gastric cancer | 2 | 231341972 | A/G | 0.062 | -0.047 | 0.024 | 0.052 | 48.183 |
| rs78099795 | Gastric cancer | 2 | 231440947 | C/T | 0.088 | -0.054 | 0.051 | 0.288 | 21.934 |
| rs13010277 | Gastric cancer | 2 | 231458411 | C/T | 0.258 | -0.055 | 0.034 | 0.100 | 31.786 |
| rs192990696 | Gastric cancer | 2 | 231353098 | C/T | 0.009 | -0.062 | 0.296 | 0.834 | 23.788 |
| rs11898768 | Gastric cancer | 2 | 231419306 | G/A | 0.077 | -0.087 | 0.053 | 0.103 | 64.256 |
| rs79780028 | Gastric cancer | 2 | 231419905 | C/A | 0.029 | -0.117 | 0.135 | 0.385 | 37.763 |
| rs186030821 | Gastric cancer | 2 | 231394168 | T/C | 0.011 | -0.135 | 0.205 | 0.509 | 41.377 |
| rs72983756 | Gastric cancer | 2 | 231335094 | A/C | 0.044 | -0.138 | 0.107 | 0.201 | 41.205 |
| rs148761169 | Gastric cancer | 2 | 231409232 | T/C | 0.026 | -0.154 | 0.139 | 0.267 | 26.115 |
| SPATS2 |  |  |  |  |  |  |  |  |  |
| rs11169042 | Gastric cancer | 12 | 49870964 | G/A | 0.434 | 0.021 | 0.021 | 0.332 | 41.276 |
| rs2720305 | Gastric cancer | 12 | 50151977 | A/G | 0.426 | 0.017 | 0.018 | 0.342 | 29.335 |
| rs7488783 | Gastric cancer | 12 | 49760499 | T/C | 0.438 | 0.004 | 0.021 | 0.835 | 48.179 |
| rs7974890 | Gastric cancer | 12 | 49652647 | C/T | 0.856 | 0.001 | 0.065 | 0.982 | 20.689 |
| rs58095611 | Gastric cancer | 12 | 49799600 | A/G | 0.175 | -0.007 | 0.059 | 0.910 | 23.125 |
| rs1269814 | Gastric cancer | 12 | 49988307 | G/T | 0.569 | -0.009 | 0.019 | 0.640 | 34.779 |
| rs12424401 | Gastric cancer | 12 | 49613965 | A/G | 0.059 | -0.215 | 0.098 | 0.028 | 20.658 |
| KRR1 |  |  |  |  |  |  |  |  |  |
| rs12819985 | Gastric cancer | 12 | 75936817 | C/T | 0.057 | 0.124 | 0.096 | 0.193 | 28.366 |
| rs112663774 | Gastric cancer | 12 | 75969948 | A/G | 0.027 | 0.093 | 0.138 | 0.502 | 34.943 |
| rs12425122 | Gastric cancer | 12 | 75862495 | A/G | 0.142 | 0.075 | 0.024 | 0.001 | 84.120 |
| rs80294010 | Gastric cancer | 12 | 75714328 | A/C | 0.089 | 0.071 | 0.025 | 0.004 | 52.225 |
| rs1842320 | Gastric cancer | 12 | 75897349 | T/C | 0.938 | 0.056 | 0.079 | 0.483 | 100.105 |
| rs7301532 | Gastric cancer | 12 | 75693751 | A/G | 0.242 | 0.045 | 0.018 | 0.012 | 56.323 |
| rs56049502 | Gastric cancer | 12 | 75927767 | A/G | 0.145 | 0.041 | 0.024 | 0.087 | 68.764 |
| rs7294516 | Gastric cancer | 12 | 75775073 | C/A | 0.939 | 0.035 | 0.069 | 0.617 | 80.640 |
| rs11180583 | Gastric cancer | 12 | 75978112 | C/T | 0.096 | 0.007 | 0.026 | 0.795 | 41.970 |
| rs34952545 | Gastric cancer | 12 | 76027147 | A/G | 0.378 | 0.006 | 0.016 | 0.711 | 35.399 |
| rs7305433 | Gastric cancer | 12 | 75848374 | A/G | 0.411 | -0.011 | 0.016 | 0.492 | 62.524 |
| rs11180553 | Gastric cancer | 12 | 75910307 | G/A | 0.258 | -0.013 | 0.018 | 0.468 | 66.210 |
| rs4882638 | Gastric cancer | 12 | 76020894 | T/C | 0.148 | -0.015 | 0.019 | 0.428 | 20.734 |
| rs12579473 | Gastric cancer | 12 | 75712662 | G/A | 0.241 | -0.032 | 0.019 | 0.094 | 29.160 |
| rs11180599 | Gastric cancer | 12 | 76013089 | C/T | 0.909 | -0.050 | 0.077 | 0.520 | 69.644 |
| DNAJC15 |  |  |  |  |  |  |  |  |  |
| rs9594847 | Gastric cancer | 13 | 43564404 | T/C | 0.010 | 0.342 | 0.235 | 0.146 | 82.077 |
| rs74786802 | Gastric cancer | 13 | 43629891 | G/A | 0.009 | 0.323 | 0.250 | 0.197 | 40.336 |
| rs117027795 | Gastric cancer | 13 | 43631705 | G/A | 0.023 | 0.216 | 0.149 | 0.148 | 107.578 |
| rs9533305 | Gastric cancer | 13 | 43496947 | T/G | 0.145 | 0.215 | 0.118 | 0.069 | 45.146 |
| rs76967284 | Gastric cancer | 13 | 43599207 | C/A | 0.018 | 0.194 | 0.134 | 0.147 | 95.031 |
| rs35793695 | Gastric cancer | 13 | 43604939 | T/C | 0.036 | 0.158 | 0.121 | 0.190 | 113.591 |
| rs76446164 | Gastric cancer | 13 | 43712700 | G/A | 0.023 | 0.142 | 0.155 | 0.360 | 45.594 |
| rs118136826 | Gastric cancer | 13 | 43631804 | A/G | 0.017 | 0.129 | 0.175 | 0.461 | 68.200 |
| rs75467447 | Gastric cancer | 13 | 43701259 | C/T | 0.020 | 0.127 | 0.160 | 0.430 | 121.833 |
| rs35217111 | Gastric cancer | 13 | 43742945 | T/C | 0.063 | 0.096 | 0.094 | 0.308 | 33.705 |
| rs75058656 | Gastric cancer | 13 | 43460619 | T/C | 0.037 | 0.096 | 0.117 | 0.413 | 22.614 |
| rs66547451 | Gastric cancer | 13 | 43636011 | C/A | 0.102 | 0.092 | 0.075 | 0.218 | 506.140 |
| rs151099697 | Gastric cancer | 13 | 43479368 | G/A | 0.035 | 0.086 | 0.127 | 0.499 | 19.746 |
| rs74971271 | Gastric cancer | 13 | 43708151 | A/G | 0.095 | 0.080 | 0.058 | 0.173 | 189.561 |
| rs2657100 | Gastric cancer | 13 | 43726100 | C/T | 0.266 | 0.067 | 0.029 | 0.023 | 68.225 |
| rs12876440 | Gastric cancer | 13 | 43546327 | C/T | 0.153 | 0.044 | 0.032 | 0.162 | 135.076 |
| rs9525756 | Gastric cancer | 13 | 43859225 | A/C | 0.165 | 0.036 | 0.036 | 0.327 | 22.505 |
| rs117333564 | Gastric cancer | 13 | 43542478 | A/G | 0.055 | 0.030 | 0.102 | 0.770 | 659.017 |
| rs76499695 | Gastric cancer | 13 | 43581524 | G/A | 0.033 | 0.029 | 0.125 | 0.819 | 329.554 |
| rs9567071 | Gastric cancer | 13 | 43513731 | C/T | 0.115 | 0.021 | 0.020 | 0.285 | 73.374 |
| rs7337195 | Gastric cancer | 13 | 43528769 | A/G | 0.475 | 0.017 | 0.016 | 0.297 | 686.724 |
| rs142564139 | Gastric cancer | 13 | 43593118 | T/C | 0.020 | 0.016 | 0.164 | 0.921 | 86.444 |
| rs2657099 | Gastric cancer | 13 | 43727388 | T/C | 0.080 | 0.013 | 0.023 | 0.584 | 24.982 |
| rs2325023 | Gastric cancer | 13 | 43700918 | C/T | 0.874 | 0.011 | 0.057 | 0.842 | 74.847 |
| rs9533241 | Gastric cancer | 13 | 43350438 | G/T | 0.108 | 0.010 | 0.032 | 0.751 | 20.723 |
| rs9533400 | Gastric cancer | 13 | 43698020 | C/T | 0.894 | 0.009 | 0.034 | 0.795 | 80.665 |
| rs117421273 | Gastric cancer | 13 | 43707497 | C/T | 0.022 | 0.009 | 0.152 | 0.954 | 88.955 |
| rs9567083 | Gastric cancer | 13 | 43550749 | A/C | 0.502 | 0.008 | 0.016 | 0.627 | 1250.663 |
| rs35865643 | Gastric cancer | 13 | 43626266 | G/A | 0.146 | 0.008 | 0.019 | 0.687 | 434.917 |
| rs17551804 | Gastric cancer | 13 | 43533155 | T/C | 0.203 | 0.007 | 0.055 | 0.900 | 296.353 |
| rs9533398 | Gastric cancer | 13 | 43694539 | C/T | 0.816 | 0.007 | 0.019 | 0.725 | 51.175 |
| rs2657116 | Gastric cancer | 13 | 43712053 | G/A | 0.587 | 0.004 | 0.018 | 0.829 | 218.778 |
| rs9567117 | Gastric cancer | 13 | 43698790 | A/G | 0.580 | 0.003 | 0.016 | 0.835 | 884.134 |
| rs148243360 | Gastric cancer | 13 | 43710696 | G/A | 0.014 | 0.002 | 0.190 | 0.992 | 100.262 |
| rs2762180 | Gastric cancer | 13 | 43749805 | C/T | 0.131 | 0.002 | 0.017 | 0.918 | 22.851 |
| rs1535906 | Gastric cancer | 13 | 43407174 | A/C | 0.724 | 0.001 | 0.019 | 0.943 | 30.515 |
| rs17459793 | Gastric cancer | 13 | 43696985 | T/C | 0.330 | -0.001 | 0.017 | 0.957 | 466.490 |
| rs9567112 | Gastric cancer | 13 | 43694558 | C/T | 0.102 | -0.001 | 0.024 | 0.957 | 68.033 |
| rs7996716 | Gastric cancer | 13 | 43464953 | C/T | 0.434 | -0.002 | 0.016 | 0.904 | 20.492 |
| rs2589317 | Gastric cancer | 13 | 43704346 | T/G | 0.191 | -0.002 | 0.020 | 0.902 | 282.071 |
| rs12430380 | Gastric cancer | 13 | 43694761 | C/T | 0.082 | -0.005 | 0.023 | 0.847 | 33.813 |
| rs2657107 | Gastric cancer | 13 | 43702139 | G/A | 0.195 | -0.006 | 0.019 | 0.767 | 54.212 |
| rs7989831 | Gastric cancer | 13 | 43368569 | C/A | 0.386 | -0.008 | 0.016 | 0.627 | 23.784 |
| rs2456241 | Gastric cancer | 13 | 43612736 | A/G | 0.466 | -0.009 | 0.016 | 0.564 | 4541.552 |
| rs117473834 | Gastric cancer | 13 | 43839083 | A/G | 0.018 | -0.010 | 0.167 | 0.954 | 33.573 |
| rs12869710 | Gastric cancer | 13 | 43754792 | C/T | 0.065 | -0.010 | 0.096 | 0.917 | 25.132 |
| rs9562464 | Gastric cancer | 13 | 43692227 | G/A | 0.058 | -0.013 | 0.024 | 0.602 | 25.167 |
| rs76977375 | Gastric cancer | 13 | 43748288 | A/G | 0.046 | -0.015 | 0.108 | 0.887 | 48.899 |
| rs12429501 | Gastric cancer | 13 | 43640373 | A/G | 0.075 | -0.016 | 0.072 | 0.824 | 361.071 |
| rs2762192 | Gastric cancer | 13 | 43718991 | A/G | 0.197 | -0.019 | 0.017 | 0.260 | 43.688 |
| rs2589312 | Gastric cancer | 13 | 43727076 | G/A | 0.628 | -0.019 | 0.018 | 0.297 | 20.009 |
| rs9315977 | Gastric cancer | 13 | 43555534 | G/A | 0.568 | -0.021 | 0.018 | 0.250 | 3116.865 |
| rs912440 | Gastric cancer | 13 | 43658498 | A/G | 0.192 | -0.025 | 0.020 | 0.197 | 91.620 |
| rs9533315 | Gastric cancer | 13 | 43519407 | A/G | 0.184 | -0.025 | 0.017 | 0.136 | 286.808 |
| rs75451283 | Gastric cancer | 13 | 43700320 | A/G | 0.040 | -0.045 | 0.113 | 0.692 | 285.546 |
| rs12428023 | Gastric cancer | 13 | 43554697 | T/C | 0.128 | -0.049 | 0.029 | 0.096 | 367.616 |
| rs146437652 | Gastric cancer | 13 | 43624311 | T/C | 0.032 | -0.054 | 0.129 | 0.678 | 26.837 |
| rs111880975 | Gastric cancer | 13 | 43726053 | G/A | 0.028 | -0.055 | 0.135 | 0.685 | 45.649 |
| rs9533332 | Gastric cancer | 13 | 43555170 | G/A | 0.140 | -0.056 | 0.064 | 0.382 | 280.590 |
| rs56686332 | Gastric cancer | 13 | 43631898 | T/C | 0.069 | -0.061 | 0.055 | 0.264 | 373.430 |
| rs77066028 | Gastric cancer | 13 | 43519699 | A/C | 0.158 | -0.063 | 0.062 | 0.303 | 969.857 |
| rs117235902 | Gastric cancer | 13 | 43693266 | A/G | 0.024 | -0.069 | 0.144 | 0.633 | 129.198 |
| rs9590726 | Gastric cancer | 13 | 43513221 | A/G | 0.041 | -0.078 | 0.115 | 0.501 | 284.767 |
| rs112737168 | Gastric cancer | 13 | 43694592 | T/C | 0.038 | -0.080 | 0.090 | 0.372 | 74.164 |
| rs139709312 | Gastric cancer | 13 | 43616221 | G/T | 0.016 | -0.081 | 0.190 | 0.668 | 91.458 |
| rs117640454 | Gastric cancer | 13 | 43606294 | A/G | 0.018 | -0.093 | 0.166 | 0.574 | 86.942 |
| rs145155880 | Gastric cancer | 13 | 43704190 | A/G | 0.028 | -0.102 | 0.140 | 0.465 | 61.271 |
| rs76328888 | Gastric cancer | 13 | 43711215 | C/T | 0.034 | -0.106 | 0.069 | 0.129 | 114.419 |
| rs9533359 | Gastric cancer | 13 | 43620773 | T/C | 0.015 | -0.137 | 0.184 | 0.458 | 54.751 |
| rs9525708 | Gastric cancer | 13 | 43496639 | T/C | 0.885 | -0.137 | 0.130 | 0.290 | 32.071 |
| rs118013577 | Gastric cancer | 13 | 43649257 | T/C | 0.049 | -0.165 | 0.103 | 0.110 | 57.211 |
| rs117713912 | Gastric cancer | 13 | 43500949 | T/C | 0.014 | -0.166 | 0.198 | 0.400 | 38.024 |
| rs73190310 | Gastric cancer | 13 | 43662605 | C/T | 0.028 | -0.170 | 0.136 | 0.214 | 87.611 |
| rs141212674 | Gastric cancer | 13 | 43568798 | C/T | 0.018 | -0.180 | 0.172 | 0.294 | 21.820 |
| rs75729086 | Gastric cancer | 13 | 43608949 | T/G | 0.011 | -0.201 | 0.215 | 0.349 | 39.298 |
| rs76911442 | Gastric cancer | 13 | 43726613 | G/A | 0.020 | -0.219 | 0.167 | 0.189 | 30.385 |
| rs75542953 | Gastric cancer | 13 | 43497206 | C/T | 0.022 | -0.228 | 0.290 | 0.432 | 56.409 |
| RNF168 |  |  |  |  |  |  |  |  |  |
| rs148186494 | Gastric cancer | 3 | 196022182 | T/C | 0.019 | 0.113 | 0.161 | 0.482 | 23.076 |
| rs4916488 | Gastric cancer | 3 | 196068034 | G/T | 0.875 | 0.064 | 0.068 | 0.344 | 21.372 |
| rs79406031 | Gastric cancer | 3 | 196248556 | T/C | 0.034 | 0.064 | 0.050 | 0.199 | 26.391 |
| rs12489885 | Gastric cancer | 3 | 196244846 | C/T | 0.315 | 0.054 | 0.030 | 0.073 | 141.225 |
| rs116811581 | Gastric cancer | 3 | 196205725 | G/A | 0.027 | 0.048 | 0.139 | 0.728 | 24.986 |
| rs4305448 | Gastric cancer | 3 | 196161828 | G/T | 0.794 | 0.039 | 0.028 | 0.155 | 98.538 |
| rs12636036 | Gastric cancer | 3 | 196300621 | G/A | 0.505 | 0.023 | 0.017 | 0.186 | 73.154 |
| rs9837772 | Gastric cancer | 3 | 196230791 | T/G | 0.715 | 0.017 | 0.018 | 0.366 | 275.595 |
| rs6779233 | Gastric cancer | 3 | 196023723 | C/A | 0.763 | 0.015 | 0.023 | 0.526 | 50.065 |
| rs11711139 | Gastric cancer | 3 | 196315260 | C/T | 0.220 | 0.009 | 0.028 | 0.752 | 30.175 |
| rs34547573 | Gastric cancer | 3 | 196231308 | A/G | 0.363 | 0.008 | 0.017 | 0.629 | 48.022 |
| rs2341336 | Gastric cancer | 3 | 196220799 | T/C | 0.861 | -0.011 | 0.040 | 0.794 | 80.879 |
| rs9861944 | Gastric cancer | 3 | 196302864 | T/C | 0.798 | -0.011 | 0.033 | 0.749 | 110.914 |
| rs13090871 | Gastric cancer | 3 | 196202326 | C/T | 0.060 | -0.020 | 0.094 | 0.831 | 63.421 |
| rs4916486 | Gastric cancer | 3 | 196064419 | C/T | 0.969 | -0.021 | 0.129 | 0.874 | 21.623 |
| rs3861958 | Gastric cancer | 3 | 196069729 | C/A | 0.746 | -0.028 | 0.039 | 0.470 | 34.439 |
| rs111886030 | Gastric cancer | 3 | 196261517 | A/G | 0.186 | -0.028 | 0.057 | 0.624 | 43.459 |
| rs73075071 | Gastric cancer | 3 | 196258510 | A/G | 0.173 | -0.032 | 0.020 | 0.117 | 72.466 |
| rs116647953 | Gastric cancer | 3 | 196179495 | T/C | 0.078 | -0.045 | 0.029 | 0.118 | 80.967 |
| rs2035531 | Gastric cancer | 3 | 196316915 | C/A | 0.346 | -0.048 | 0.086 | 0.577 | 38.287 |
| rs115191658 | Gastric cancer | 3 | 196172850 | T/C | 0.027 | -0.105 | 0.139 | 0.450 | 20.334 |
| rs142316510 | Gastric cancer | 3 | 196208470 | T/G | 0.023 | -0.406 | 0.182 | 0.026 | 28.907 |
| LCOR |  |  |  |  |  |  |  |  |  |
| rs11188914 | Gastric cancer | 10 | 98538609 | C/T | 0.168 | 0.032 | 0.017 | 0.053 | 28.440 |
| rs7895214 | Gastric cancer | 10 | 98595326 | T/G | 0.184 | 0.015 | 0.019 | 0.427 | 21.226 |
| rs4606419 | Gastric cancer | 10 | 98747114 | A/C | 0.172 | 0.009 | 0.019 | 0.644 | 22.250 |
| rs56052045 | Gastric cancer | 10 | 98536552 | A/C | 0.094 | -0.056 | 0.027 | 0.038 | 23.826 |
| GLB1 |  |  |  |  |  |  |  |  |  |
| rs75470361 | Gastric cancer | 3 | 33101840 | G/A | 0.022 | 0.141 | 0.151 | 0.351 | 28.204 |
| rs79910159 | Gastric cancer | 3 | 33123411 | C/T | 0.013 | 0.097 | 0.198 | 0.625 | 23.008 |
| rs187339669 | Gastric cancer | 3 | 32901911 | A/G | 0.024 | 0.077 | 0.151 | 0.610 | 24.532 |
| rs78949515 | Gastric cancer | 3 | 33204720 | G/A | 0.020 | 0.063 | 0.158 | 0.692 | 47.751 |
| rs141884379 | Gastric cancer | 3 | 32899191 | G/A | 0.085 | 0.049 | 0.081 | 0.542 | 27.826 |
| rs112138222 | Gastric cancer | 3 | 33012981 | A/G | 0.099 | 0.048 | 0.079 | 0.544 | 25.378 |
| rs7628482 | Gastric cancer | 3 | 33336370 | T/C | 0.160 | 0.031 | 0.075 | 0.676 | 32.879 |
| rs79991185 | Gastric cancer | 3 | 33189782 | C/T | 0.034 | 0.031 | 0.123 | 0.802 | 91.918 |
| rs75580901 | Gastric cancer | 3 | 33027154 | T/C | 0.065 | 0.027 | 0.026 | 0.302 | 90.988 |
| rs113597451 | Gastric cancer | 3 | 33151918 | A/G | 0.142 | 0.024 | 0.065 | 0.712 | 45.834 |
| rs62250510 | Gastric cancer | 3 | 33147272 | G/A | 0.063 | 0.020 | 0.055 | 0.713 | 97.131 |
| rs62250478 | Gastric cancer | 3 | 33100491 | C/T | 0.499 | 0.020 | 0.020 | 0.312 | 115.454 |
| rs4423716 | Gastric cancer | 3 | 33102381 | G/A | 0.612 | 0.019 | 0.016 | 0.245 | 167.749 |
| rs60382153 | Gastric cancer | 3 | 33014135 | T/C | 0.395 | 0.019 | 0.022 | 0.399 | 76.128 |
| rs112871213 | Gastric cancer | 3 | 33014606 | A/C | 0.042 | 0.015 | 0.031 | 0.634 | 32.011 |
| rs34429545 | Gastric cancer | 3 | 33164092 | G/T | 0.240 | 0.014 | 0.018 | 0.428 | 37.255 |
| rs9828592 | Gastric cancer | 3 | 33044339 | C/T | 0.434 | 0.014 | 0.019 | 0.474 | 306.681 |
| rs61381898 | Gastric cancer | 3 | 33032482 | C/T | 0.218 | 0.013 | 0.027 | 0.639 | 97.056 |
| rs145724286 | Gastric cancer | 3 | 33045292 | C/T | 0.032 | 0.012 | 0.035 | 0.729 | 81.491 |
| rs35480293 | Gastric cancer | 3 | 33087395 | A/C | 0.255 | 0.011 | 0.017 | 0.504 | 285.139 |
| rs151300066 | Gastric cancer | 3 | 33035263 | A/G | 0.055 | 0.007 | 0.058 | 0.899 | 92.897 |
| rs2139197 | Gastric cancer | 3 | 32888851 | A/G | 0.305 | 0.007 | 0.016 | 0.677 | 25.468 |
| rs80207834 | Gastric cancer | 3 | 33232353 | A/C | 0.289 | 0.006 | 0.016 | 0.712 | 38.039 |
| rs13065359 | Gastric cancer | 3 | 33429404 | A/C | 0.354 | 0.004 | 0.016 | 0.805 | 28.901 |
| rs9846453 | Gastric cancer | 3 | 33014368 | G/A | 0.185 | 0.004 | 0.059 | 0.948 | 31.697 |
| rs11717007 | Gastric cancer | 3 | 33070666 | C/A | 0.247 | 0.003 | 0.019 | 0.865 | 449.477 |
| rs6773595 | Gastric cancer | 3 | 33194138 | C/T | 0.824 | 0.003 | 0.059 | 0.965 | 192.057 |
| rs2228428 | Gastric cancer | 3 | 32995928 | T/C | 0.230 | 0.002 | 0.034 | 0.957 | 22.574 |
| rs6764245 | Gastric cancer | 3 | 33019374 | A/G | 0.241 | 0.001 | 0.022 | 0.961 | 55.648 |
| rs7616228 | Gastric cancer | 3 | 33066683 | T/C | 0.597 | 0.001 | 0.016 | 0.946 | 100.416 |
| rs6777787 | Gastric cancer | 3 | 33057103 | G/A | 0.415 | -0.002 | 0.018 | 0.903 | 179.161 |
| rs7611260 | Gastric cancer | 3 | 33025110 | T/C | 0.370 | -0.002 | 0.016 | 0.895 | 169.943 |
| rs60761526 | Gastric cancer | 3 | 33227781 | G/A | 0.303 | -0.002 | 0.016 | 0.881 | 87.908 |
| rs75926793 | Gastric cancer | 3 | 33250469 | C/T | 0.048 | -0.003 | 0.105 | 0.981 | 28.283 |
| rs6778706 | Gastric cancer | 3 | 33024069 | A/G | 0.969 | -0.003 | 0.032 | 0.924 | 43.340 |
| rs12186107 | Gastric cancer | 3 | 33014036 | T/C | 0.186 | -0.003 | 0.020 | 0.865 | 81.641 |
| rs35660594 | Gastric cancer | 3 | 33200021 | T/C | 0.445 | -0.016 | 0.017 | 0.355 | 77.056 |
| rs35548241 | Gastric cancer | 3 | 33190556 | G/A | 0.362 | -0.018 | 0.016 | 0.266 | 203.797 |
| rs72856153 | Gastric cancer | 3 | 33111182 | G/T | 0.175 | -0.020 | 0.017 | 0.248 | 44.477 |
| rs13068113 | Gastric cancer | 3 | 33211735 | A/G | 0.135 | -0.023 | 0.019 | 0.238 | 134.558 |
| rs78487987 | Gastric cancer | 3 | 33130060 | A/G | 0.083 | -0.026 | 0.082 | 0.753 | 73.238 |
| rs79336262 | Gastric cancer | 3 | 32787771 | G/A | 0.054 | -0.038 | 0.099 | 0.704 | 26.392 |
| rs141634749 | Gastric cancer | 3 | 33014311 | T/C | 0.053 | -0.041 | 0.103 | 0.688 | 46.420 |
| rs35289681 | Gastric cancer | 3 | 33110383 | A/G | 0.028 | -0.043 | 0.135 | 0.750 | 48.579 |
| rs9827453 | Gastric cancer | 3 | 33013066 | T/C | 0.021 | -0.154 | 0.157 | 0.329 | 78.075 |
| ITGB2 |  |  |  |  |  |  |  |  |  |
| rs76289557 | Gastric cancer | 21 | 46247194 | T/C | 0.012 | 0.420 | 0.213 | 0.049 | 53.884 |
| rs77033838 | Gastric cancer | 21 | 46316120 | A/G | 0.027 | 0.223 | 0.139 | 0.110 | 44.219 |
| rs73239712 | Gastric cancer | 21 | 46310545 | A/G | 0.019 | 0.211 | 0.294 | 0.474 | 188.021 |
| rs117978955 | Gastric cancer | 21 | 46410121 | T/C | 0.075 | 0.180 | 0.085 | 0.034 | 88.759 |
| rs62214472 | Gastric cancer | 21 | 46343215 | T/C | 0.012 | 0.180 | 0.212 | 0.396 | 36.573 |
| rs7282671 | Gastric cancer | 21 | 46439894 | T/C | 0.131 | 0.106 | 0.040 | 0.007 | 21.125 |
| rs2838683 | Gastric cancer | 21 | 46201961 | C/T | 0.042 | 0.103 | 0.112 | 0.358 | 30.804 |
| rs116869330 | Gastric cancer | 21 | 46564075 | A/G | 0.025 | 0.089 | 0.120 | 0.456 | 24.143 |
| rs76410874 | Gastric cancer | 21 | 46378941 | A/G | 0.045 | 0.070 | 0.048 | 0.143 | 23.722 |
| rs71324410 | Gastric cancer | 21 | 46596639 | G/A | 0.093 | 0.066 | 0.077 | 0.390 | 29.725 |
| rs35934861 | Gastric cancer | 21 | 46321207 | A/G | 0.242 | 0.055 | 0.026 | 0.032 | 1109.303 |
| rs869708 | Gastric cancer | 21 | 46434954 | T/C | 0.062 | 0.050 | 0.092 | 0.588 | 34.532 |
| rs34552233 | Gastric cancer | 21 | 46299849 | T/C | 0.162 | 0.045 | 0.018 | 0.011 | 470.650 |
| rs79637556 | Gastric cancer | 21 | 46376807 | A/G | 0.040 | 0.040 | 0.114 | 0.724 | 26.898 |
| rs2230528 | Gastric cancer | 21 | 46320313 | T/C | 0.190 | 0.036 | 0.024 | 0.132 | 341.282 |
| rs74727741 | Gastric cancer | 21 | 46138631 | A/G | 0.202 | 0.030 | 0.056 | 0.597 | 29.151 |
| rs28493119 | Gastric cancer | 21 | 46178862 | T/C | 0.250 | 0.027 | 0.018 | 0.136 | 142.212 |
| rs991340 | Gastric cancer | 21 | 46350779 | A/G | 0.225 | 0.026 | 0.026 | 0.308 | 60.823 |
| rs2838704 | Gastric cancer | 21 | 46264375 | C/A | 0.413 | 0.025 | 0.016 | 0.114 | 159.186 |
| rs4818972 | Gastric cancer | 21 | 46176838 | A/G | 0.214 | 0.022 | 0.016 | 0.175 | 23.582 |
| rs428428 | Gastric cancer | 21 | 46219184 | T/C | 0.131 | 0.021 | 0.067 | 0.748 | 26.594 |
| rs990594 | Gastric cancer | 21 | 46346540 | C/T | 0.210 | 0.019 | 0.024 | 0.430 | 173.618 |
| rs12626397 | Gastric cancer | 21 | 46336082 | C/T | 0.376 | 0.019 | 0.017 | 0.262 | 115.363 |
| rs3746972 | Gastric cancer | 21 | 46327835 | G/A | 0.159 | 0.019 | 0.026 | 0.461 | 1079.935 |
| rs8133302 | Gastric cancer | 21 | 46255215 | C/A | 0.289 | 0.019 | 0.016 | 0.246 | 86.691 |
| rs1893562 | Gastric cancer | 21 | 46557203 | C/T | 0.544 | 0.019 | 0.016 | 0.258 | 25.976 |
| rs760453 | Gastric cancer | 21 | 46340512 | A/G | 0.443 | 0.018 | 0.016 | 0.250 | 362.236 |
| rs7281781 | Gastric cancer | 21 | 46336548 | C/T | 0.218 | 0.015 | 0.019 | 0.412 | 412.954 |
| rs9808658 | Gastric cancer | 21 | 46180860 | T/G | 0.580 | 0.015 | 0.018 | 0.415 | 85.066 |
| rs8132813 | Gastric cancer | 21 | 46483467 | C/A | 0.230 | 0.015 | 0.030 | 0.631 | 69.495 |
| rs13049630 | Gastric cancer | 21 | 46244215 | T/C | 0.233 | 0.014 | 0.017 | 0.393 | 113.508 |
| rs56056043 | Gastric cancer | 21 | 46337271 | C/T | 0.150 | 0.012 | 0.024 | 0.632 | 28.883 |
| rs114653592 | Gastric cancer | 21 | 46057417 | C/A | 0.285 | 0.011 | 0.019 | 0.581 | 23.895 |
| rs55678595 | Gastric cancer | 21 | 46317846 | G/A | 0.230 | 0.011 | 0.018 | 0.552 | 532.559 |
| rs9982678 | Gastric cancer | 21 | 46420024 | T/C | 0.349 | 0.008 | 0.017 | 0.618 | 38.333 |
| rs760458 | Gastric cancer | 21 | 46328917 | A/C | 0.225 | 0.007 | 0.018 | 0.688 | 455.616 |
| rs13048147 | Gastric cancer | 21 | 46417461 | T/C | 0.644 | 0.007 | 0.019 | 0.719 | 35.314 |
| rs13048981 | Gastric cancer | 21 | 46418100 | T/C | 0.077 | 0.006 | 0.042 | 0.879 | 20.147 |
| rs3788157 | Gastric cancer | 21 | 46510708 | C/T | 0.207 | 0.006 | 0.021 | 0.788 | 20.454 |
| rs12482063 | Gastric cancer | 21 | 46369040 | A/G | 0.162 | 0.004 | 0.062 | 0.945 | 23.650 |
| rs7281925 | Gastric cancer | 21 | 46336645 | C/T | 0.346 | 0.004 | 0.019 | 0.846 | 55.175 |
| rs235269 | Gastric cancer | 21 | 46211664 | G/A | 0.652 | 0.003 | 0.020 | 0.894 | 56.295 |
| rs28885143 | Gastric cancer | 21 | 46427266 | T/C | 0.143 | 0.001 | 0.021 | 0.951 | 119.461 |
| rs1467854 | Gastric cancer | 21 | 46420741 | T/C | 0.083 | 0.001 | 0.024 | 0.982 | 82.867 |
| rs760462 | Gastric cancer | 21 | 46328099 | C/T | 0.797 | -0.001 | 0.018 | 0.977 | 4590.512 |
| rs2838729 | Gastric cancer | 21 | 46319417 | G/A | 0.505 | -0.001 | 0.016 | 0.972 | 580.452 |
| rs55865320 | Gastric cancer | 21 | 46321659 | A/C | 0.138 | -0.001 | 0.033 | 0.980 | 210.900 |
| rs55829486 | Gastric cancer | 21 | 46344216 | G/A | 0.234 | -0.002 | 0.018 | 0.909 | 98.656 |
| rs74397353 | Gastric cancer | 21 | 46310306 | A/G | 0.040 | -0.002 | 0.114 | 0.984 | 427.256 |
| rs72613652 | Gastric cancer | 21 | 46484612 | C/T | 0.288 | -0.003 | 0.016 | 0.877 | 23.561 |
| rs17313439 | Gastric cancer | 21 | 46375354 | G/A | 0.185 | -0.003 | 0.022 | 0.878 | 26.799 |
| rs760460 | Gastric cancer | 21 | 46328820 | T/C | 0.596 | -0.006 | 0.017 | 0.745 | 1836.916 |
| rs78365502 | Gastric cancer | 21 | 46325126 | T/G | 0.067 | -0.006 | 0.026 | 0.834 | 89.819 |
| rs170961 | Gastric cancer | 21 | 46140966 | G/A | 0.394 | -0.007 | 0.021 | 0.730 | 22.470 |
| rs13047060 | Gastric cancer | 21 | 46424507 | T/G | 0.556 | -0.007 | 0.018 | 0.683 | 57.714 |
| rs1160263 | Gastric cancer | 21 | 46306138 | T/G | 0.203 | -0.008 | 0.019 | 0.672 | 47.864 |
| rs4308190 | Gastric cancer | 21 | 46444287 | T/C | 0.968 | -0.009 | 0.132 | 0.946 | 28.563 |
| rs9978380 | Gastric cancer | 21 | 46416731 | A/C | 0.600 | -0.010 | 0.017 | 0.560 | 113.913 |
| rs118146365 | Gastric cancer | 21 | 46451637 | T/C | 0.036 | -0.011 | 0.121 | 0.931 | 25.160 |
| rs1474552 | Gastric cancer | 21 | 46337290 | C/T | 0.116 | -0.011 | 0.021 | 0.596 | 141.156 |
| rs11701589 | Gastric cancer | 21 | 46404727 | T/C | 0.112 | -0.012 | 0.025 | 0.634 | 127.945 |
| rs73232668 | Gastric cancer | 21 | 46602874 | G/T | 0.069 | -0.012 | 0.089 | 0.890 | 45.326 |
| rs75974293 | Gastric cancer | 21 | 46341592 | A/C | 0.129 | -0.013 | 0.070 | 0.858 | 35.708 |
| rs117098249 | Gastric cancer | 21 | 46571273 | G/A | 0.011 | -0.013 | 0.214 | 0.952 | 21.604 |
| rs9978207 | Gastric cancer | 21 | 46351012 | C/T | 0.115 | -0.014 | 0.025 | 0.567 | 34.399 |
| rs7282299 | Gastric cancer | 21 | 46484228 | T/C | 0.346 | -0.015 | 0.017 | 0.390 | 20.065 |
| rs8126575 | Gastric cancer | 21 | 46435610 | G/T | 0.227 | -0.015 | 0.016 | 0.354 | 21.273 |
| rs235334 | Gastric cancer | 21 | 46235174 | T/C | 0.508 | -0.016 | 0.018 | 0.362 | 28.823 |
| rs73230641 | Gastric cancer | 21 | 46483069 | C/T | 0.119 | -0.017 | 0.069 | 0.801 | 65.895 |
| rs180318 | Gastric cancer | 21 | 46307495 | C/T | 0.599 | -0.018 | 0.045 | 0.691 | 100.840 |
| rs2838730 | Gastric cancer | 21 | 46319492 | A/G | 0.423 | -0.020 | 0.017 | 0.242 | 45.442 |
| rs34580582 | Gastric cancer | 21 | 46330183 | C/T | 0.046 | -0.022 | 0.033 | 0.513 | 59.592 |
| rs2013275 | Gastric cancer | 21 | 46334472 | T/C | 0.444 | -0.023 | 0.016 | 0.152 | 127.816 |
| rs2235133 | Gastric cancer | 21 | 46321172 | T/C | 0.499 | -0.023 | 0.016 | 0.153 | 1298.798 |
| rs73234878 | Gastric cancer | 21 | 46121975 | A/C | 0.018 | -0.030 | 0.171 | 0.863 | 20.083 |
| rs685674 | Gastric cancer | 21 | 46265051 | A/C | 0.268 | -0.042 | 0.020 | 0.035 | 46.014 |
| rs2838671 | Gastric cancer | 21 | 46176941 | A/G | 0.081 | -0.045 | 0.083 | 0.586 | 59.455 |
| rs146114096 | Gastric cancer | 21 | 46339143 | T/C | 0.007 | -0.046 | 0.284 | 0.871 | 27.833 |
| rs2075883 | Gastric cancer | 21 | 46310445 | A/G | 0.040 | -0.049 | 0.034 | 0.143 | 41.145 |
| rs112403172 | Gastric cancer | 21 | 46329861 | T/C | 0.016 | -0.051 | 0.181 | 0.779 | 30.607 |
| rs235263 | Gastric cancer | 21 | 46262875 | A/G | 0.049 | -0.058 | 0.031 | 0.056 | 42.355 |
| rs145463127 | Gastric cancer | 21 | 46331472 | T/C | 0.007 | -0.063 | 0.276 | 0.819 | 52.937 |
| rs173098 | Gastric cancer | 21 | 46307551 | A/G | 0.222 | -0.072 | 0.054 | 0.179 | 112.799 |
| rs112625673 | Gastric cancer | 21 | 46060808 | G/A | 0.040 | -0.073 | 0.117 | 0.530 | 21.530 |
| rs118181594 | Gastric cancer | 21 | 46334165 | G/A | 0.026 | -0.087 | 0.139 | 0.532 | 102.453 |
| rs117843275 | Gastric cancer | 21 | 46229341 | G/A | 0.016 | -0.094 | 0.182 | 0.606 | 20.026 |
| rs235321 | Gastric cancer | 21 | 46277357 | G/A | 0.910 | -0.110 | 0.079 | 0.166 | 95.186 |
| rs11700696 | Gastric cancer | 21 | 46417775 | A/G | 0.019 | -0.112 | 0.166 | 0.499 | 113.742 |
| rs79249663 | Gastric cancer | 21 | 46320721 | T/C | 0.019 | -0.115 | 0.163 | 0.482 | 35.682 |
| rs187282135 | Gastric cancer | 21 | 46488055 | C/T | 0.018 | -0.131 | 0.164 | 0.427 | 39.607 |
| rs118156675 | Gastric cancer | 21 | 46228528 | A/G | 0.031 | -0.137 | 0.128 | 0.286 | 71.436 |
| rs62221516 | Gastric cancer | 21 | 46310416 | T/G | 0.063 | -0.142 | 0.092 | 0.122 | 36.942 |
| rs74428766 | Gastric cancer | 21 | 46547403 | T/C | 0.019 | -0.144 | 0.295 | 0.625 | 33.150 |
| rs80325088 | Gastric cancer | 21 | 46329175 | T/C | 0.051 | -0.147 | 0.102 | 0.149 | 780.864 |
| rs117723815 | Gastric cancer | 21 | 46415671 | G/A | 0.016 | -0.152 | 0.183 | 0.407 | 27.403 |
| rs235357 | Gastric cancer | 21 | 46192515 | A/G | 0.974 | -0.159 | 0.141 | 0.260 | 33.762 |
| rs55944874 | Gastric cancer | 21 | 46415309 | T/C | 0.014 | -0.196 | 0.192 | 0.308 | 37.346 |
| rs192480700 | Gastric cancer | 21 | 46273331 | G/A | 0.013 | -0.237 | 0.209 | 0.257 | 43.932 |
| SUZ12 |  |  |  |  |  |  |  |  |  |
| rs72821912 | Gastric cancer | 17 | 30183388 | T/C | 0.035 | 0.218 | 0.122 | 0.073 | 21.676 |
| rs7223225 | Gastric cancer | 17 | 30173336 | C/T | 0.961 | 0.099 | 0.114 | 0.385 | 20.014 |
| rs2428341 | Gastric cancer | 17 | 30317295 | T/C | 0.143 | 0.030 | 0.063 | 0.635 | 42.922 |
| rs12941700 | Gastric cancer | 17 | 30185022 | G/A | 0.869 | -0.007 | 0.022 | 0.741 | 45.750 |
| rs17182651 | Gastric cancer | 17 | 30547537 | A/G | 0.111 | -0.009 | 0.071 | 0.902 | 27.411 |
| rs6505277 | Gastric cancer | 17 | 30420528 | G/A | 0.110 | -0.027 | 0.071 | 0.705 | 26.819 |
| rs112974258 | Gastric cancer | 17 | 30350798 | C/T | 0.040 | -0.172 | 0.114 | 0.129 | 26.937 |
| BAG4 |  |  |  |  |  |  |  |  |  |
| rs76690632 | Gastric cancer | 8 | 38258621 | T/C | 0.018 | 0.235 | 0.168 | 0.164 | 31.510 |
| rs138012741 | Gastric cancer | 8 | 38323693 | G/A | 0.028 | 0.181 | 0.138 | 0.188 | 24.416 |
| rs62505465 | Gastric cancer | 8 | 38248813 | G/T | 0.068 | 0.164 | 0.107 | 0.126 | 51.094 |
| rs117756469 | Gastric cancer | 8 | 37941880 | T/C | 0.027 | 0.133 | 0.138 | 0.337 | 24.305 |
| rs77732910 | Gastric cancer | 8 | 38147091 | C/T | 0.052 | 0.085 | 0.101 | 0.400 | 112.085 |
| rs117486616 | Gastric cancer | 8 | 38194208 | C/T | 0.023 | 0.058 | 0.149 | 0.698 | 53.403 |
| rs78581625 | Gastric cancer | 8 | 37933834 | A/G | 0.123 | 0.047 | 0.027 | 0.081 | 67.838 |
| rs141471977 | Gastric cancer | 8 | 38225226 | C/T | 0.062 | 0.033 | 0.093 | 0.722 | 48.665 |
| rs35866749 | Gastric cancer | 8 | 38070128 | G/A | 0.137 | 0.032 | 0.028 | 0.240 | 122.760 |
| rs74302890 | Gastric cancer | 8 | 38178918 | T/C | 0.141 | 0.023 | 0.023 | 0.319 | 22.701 |
| rs57011135 | Gastric cancer | 8 | 38193011 | C/A | 0.242 | 0.015 | 0.017 | 0.375 | 667.834 |
| rs11777067 | Gastric cancer | 8 | 38298647 | T/C | 0.239 | 0.013 | 0.017 | 0.444 | 464.381 |
| rs4733930 | Gastric cancer | 8 | 38311001 | T/C | 0.398 | 0.011 | 0.017 | 0.532 | 202.394 |
| rs17175708 | Gastric cancer | 8 | 38325131 | A/G | 0.401 | -0.009 | 0.016 | 0.591 | 76.475 |
| rs7837532 | Gastric cancer | 8 | 37953386 | G/A | 0.093 | -0.012 | 0.019 | 0.511 | 26.458 |
| rs2720044 | Gastric cancer | 8 | 37980587 | C/A | 0.180 | -0.016 | 0.020 | 0.423 | 32.609 |
| rs117349296 | Gastric cancer | 8 | 38080951 | T/G | 0.026 | -0.019 | 0.139 | 0.894 | 29.113 |
| rs74322494 | Gastric cancer | 8 | 38345875 | G/T | 0.125 | -0.030 | 0.068 | 0.663 | 35.530 |
| rs139832760 | Gastric cancer | 8 | 38357223 | T/C | 0.025 | -0.034 | 0.144 | 0.815 | 20.802 |
| rs117404237 | Gastric cancer | 8 | 38213632 | G/A | 0.027 | -0.040 | 0.138 | 0.770 | 27.033 |
| rs113092086 | Gastric cancer | 8 | 38143539 | T/C | 0.014 | -0.061 | 0.194 | 0.752 | 31.649 |
| rs3758101 | Gastric cancer | 8 | 38317476 | C/A | 0.780 | -0.085 | 0.054 | 0.113 | 98.015 |
| rs4647910 | Gastric cancer | 8 | 38279518 | A/G | 0.013 | -0.088 | 0.202 | 0.663 | 33.893 |
| UBLCP1 |  |  |  |  |  |  |  |  |  |
| rs13158921 | Gastric cancer | 5 | 158649872 | T/C | 0.015 | 0.456 | 0.188 | 0.015 | 37.056 |
| rs13154564 | Gastric cancer | 5 | 158539430 | C/T | 0.019 | 0.360 | 0.165 | 0.030 | 29.325 |
| rs116034218 | Gastric cancer | 5 | 158801800 | A/G | 0.026 | 0.304 | 0.143 | 0.034 | 29.670 |
| rs13162687 | Gastric cancer | 5 | 158765041 | G/A | 0.032 | 0.160 | 0.130 | 0.219 | 57.856 |
| rs17720456 | Gastric cancer | 5 | 158631274 | T/C | 0.028 | 0.156 | 0.135 | 0.246 | 51.761 |
| rs116555563 | Gastric cancer | 5 | 158503209 | T/C | 0.014 | 0.105 | 0.193 | 0.587 | 32.002 |
| rs2116785 | Gastric cancer | 5 | 158618933 | T/C | 0.045 | 0.028 | 0.055 | 0.612 | 80.890 |
| rs6870828 | Gastric cancer | 5 | 158738512 | T/C | 0.589 | 0.021 | 0.018 | 0.265 | 160.834 |
| rs11740796 | Gastric cancer | 5 | 158547841 | G/A | 0.706 | 0.017 | 0.025 | 0.485 | 29.545 |
| rs35268985 | Gastric cancer | 5 | 158445402 | C/T | 0.143 | 0.009 | 0.023 | 0.716 | 28.209 |
| rs2546893 | Gastric cancer | 5 | 158755960 | A/G | 0.478 | 0.008 | 0.016 | 0.601 | 148.175 |
| rs12652431 | Gastric cancer | 5 | 158722094 | G/A | 0.082 | 0.008 | 0.038 | 0.837 | 288.589 |
| rs13169581 | Gastric cancer | 5 | 158827526 | T/C | 0.336 | 0.003 | 0.018 | 0.850 | 21.505 |
| rs6896259 | Gastric cancer | 5 | 158862681 | G/A | 0.599 | 0.003 | 0.016 | 0.845 | 38.327 |
| rs2161417 | Gastric cancer | 5 | 158862238 | T/G | 0.329 | 0.002 | 0.018 | 0.930 | 54.492 |
| rs150259821 | Gastric cancer | 5 | 158807617 | T/C | 0.020 | 0.000 | 0.164 | 0.998 | 78.742 |
| rs3846687 | Gastric cancer | 5 | 158679579 | T/C | 0.334 | -0.002 | 0.017 | 0.909 | 123.053 |
| rs13157773 | Gastric cancer | 5 | 158800723 | T/C | 0.276 | -0.008 | 0.019 | 0.663 | 180.846 |
| rs9313802 | Gastric cancer | 5 | 158576915 | A/G | 0.450 | -0.011 | 0.016 | 0.482 | 39.190 |
| rs4921495 | Gastric cancer | 5 | 158844664 | A/G | 0.165 | -0.012 | 0.022 | 0.573 | 35.722 |
| rs918521 | Gastric cancer | 5 | 158826247 | G/A | 0.807 | -0.014 | 0.057 | 0.806 | 22.580 |
| rs4921213 | Gastric cancer | 5 | 158733619 | A/G | 0.560 | -0.017 | 0.018 | 0.347 | 279.279 |
| rs17056526 | Gastric cancer | 5 | 158501850 | T/C | 0.246 | -0.018 | 0.018 | 0.320 | 30.907 |
| rs140589473 | Gastric cancer | 5 | 158567662 | C/T | 0.141 | -0.023 | 0.035 | 0.509 | 76.513 |
| rs76735821 | Gastric cancer | 5 | 158543750 | A/G | 0.051 | -0.024 | 0.033 | 0.471 | 24.006 |
| rs7727579 | Gastric cancer | 5 | 158558685 | T/C | 0.121 | -0.028 | 0.069 | 0.686 | 23.061 |
| rs17664544 | Gastric cancer | 5 | 158673506 | C/T | 0.052 | -0.037 | 0.100 | 0.709 | 23.067 |
| rs116061799 | Gastric cancer | 5 | 158550385 | G/T | 0.046 | -0.079 | 0.107 | 0.460 | 41.157 |
| rs114042633 | Gastric cancer | 5 | 158858275 | G/A | 0.031 | -0.086 | 0.134 | 0.521 | 43.106 |
| rs78297373 | Gastric cancer | 5 | 158660872 | A/G | 0.067 | -0.105 | 0.089 | 0.239 | 47.479 |
| rs115561952 | Gastric cancer | 5 | 158602331 | T/C | 0.015 | -0.108 | 0.189 | 0.566 | 30.336 |
| rs56240842 | Gastric cancer | 5 | 158803479 | A/G | 0.041 | -0.167 | 0.113 | 0.140 | 30.189 |
| rs79096996 | Gastric cancer | 5 | 158923424 | T/G | 0.023 | -0.185 | 0.151 | 0.221 | 30.106 |
| rs76463667 | Gastric cancer | 5 | 158448858 | G/A | 0.031 | -0.198 | 0.130 | 0.127 | 21.618 |
| rs78128874 | Gastric cancer | 5 | 158863876 | C/T | 0.019 | -0.323 | 0.289 | 0.263 | 25.105 |
| MRPL20 |  |  |  |  |  |  |  |  |  |
| rs1240588 | Gastric cancer | 1 | 1385573 | T/C | 0.065 | 0.246 | 0.160 | 0.126 | 21.509 |
| rs143644995 | Gastric cancer | 1 | 1305807 | A/C | 0.015 | 0.127 | 0.186 | 0.497 | 20.673 |
| rs115712376 | Gastric cancer | 1 | 1191651 | G/A | 0.017 | 0.099 | 0.178 | 0.579 | 47.713 |
| rs114720027 | Gastric cancer | 1 | 1226690 | T/C | 0.029 | 0.091 | 0.136 | 0.505 | 147.311 |
| rs71628954 | Gastric cancer | 1 | 1280808 | T/C | 0.031 | 0.076 | 0.135 | 0.571 | 84.477 |
| rs1153105 | Gastric cancer | 1 | 1415099 | C/T | 0.143 | 0.066 | 0.084 | 0.435 | 82.583 |
| rs2239609 | Gastric cancer | 1 | 1227244 | A/G | 0.113 | 0.060 | 0.019 | 0.002 | 98.361 |
| rs143070056 | Gastric cancer | 1 | 1294375 | T/G | 0.022 | 0.042 | 0.152 | 0.783 | 37.510 |
| rs6694994 | Gastric cancer | 1 | 1468178 | C/T | 0.457 | 0.039 | 0.047 | 0.403 | 46.276 |
| rs115834287 | Gastric cancer | 1 | 1219941 | A/G | 0.059 | 0.036 | 0.097 | 0.710 | 103.228 |
| rs3766176 | Gastric cancer | 1 | 1486903 | C/T | 0.293 | 0.036 | 0.018 | 0.043 | 22.453 |
| rs6603782 | Gastric cancer | 1 | 1171417 | T/C | 0.342 | 0.031 | 0.047 | 0.517 | 134.333 |
| rs61776787 | Gastric cancer | 1 | 1606571 | G/A | 0.736 | 0.031 | 0.053 | 0.566 | 21.975 |
| rs2765030 | Gastric cancer | 1 | 1316648 | T/C | 0.890 | 0.027 | 0.023 | 0.253 | 38.107 |
| rs1240743 | Gastric cancer | 1 | 1333436 | A/C | 0.584 | 0.018 | 0.084 | 0.828 | 36.972 |
| rs3813201 | Gastric cancer | 1 | 1151232 | C/T | 0.199 | 0.017 | 0.018 | 0.324 | 145.494 |
| rs6603787 | Gastric cancer | 1 | 1188225 | T/G | 0.094 | 0.017 | 0.023 | 0.449 | 197.231 |
| rs2291885 | Gastric cancer | 1 | 1367735 | A/G | 0.354 | 0.013 | 0.024 | 0.579 | 268.261 |
| rs150793469 | Gastric cancer | 1 | 1437069 | A/C | 0.015 | 0.009 | 0.337 | 0.979 | 26.511 |
| rs72894031 | Gastric cancer | 1 | 1135651 | T/C | 0.063 | 0.004 | 0.023 | 0.877 | 26.656 |
| rs74046665 | Gastric cancer | 1 | 1215078 | G/A | 0.227 | -0.003 | 0.017 | 0.846 | 37.537 |
| rs2144440 | Gastric cancer | 1 | 1220136 | G/A | 0.199 | -0.005 | 0.017 | 0.795 | 52.272 |
| rs77950429 | Gastric cancer | 1 | 1120536 | A/G | 0.070 | -0.010 | 0.022 | 0.650 | 21.737 |
| rs28613513 | Gastric cancer | 1 | 1112810 | T/G | 0.093 | -0.016 | 0.023 | 0.483 | 39.472 |
| rs11584885 | Gastric cancer | 1 | 1099437 | A/G | 0.292 | -0.021 | 0.050 | 0.682 | 21.964 |
| rs1815606 | Gastric cancer | 1 | 1140435 | T/G | 0.484 | -0.024 | 0.032 | 0.457 | 79.210 |
| rs9442387 | Gastric cancer | 1 | 1110586 | C/T | 0.480 | -0.029 | 0.025 | 0.244 | 47.336 |
| rs141483437 | Gastric cancer | 1 | 1294470 | T/G | 0.032 | -0.036 | 0.131 | 0.786 | 119.245 |
| rs6681228 | Gastric cancer | 1 | 1469263 | T/G | 0.154 | -0.042 | 0.019 | 0.032 | 24.723 |
| rs11804831 | Gastric cancer | 1 | 1194804 | C/T | 0.218 | -0.049 | 0.054 | 0.368 | 233.731 |
| rs2273274 | Gastric cancer | 1 | 1226102 | A/G | 0.149 | -0.054 | 0.020 | 0.006 | 33.508 |
| rs2649599 | Gastric cancer | 1 | 1305493 | G/A | 0.586 | -0.064 | 0.049 | 0.190 | 447.453 |
| rs61774864 | Gastric cancer | 1 | 1495259 | A/G | 0.028 | -0.223 | 0.245 | 0.363 | 45.482 |
| rs28697106 | Gastric cancer | 1 | 1397140 | A/C | 0.025 | -0.283 | 0.142 | 0.046 | 24.235 |
| KPNB1 |  |  |  |  |  |  |  |  |  |
| rs56144785 | Gastric cancer | 17 | 45746819 | G/T | 0.043 | 0.138 | 0.194 | 0.478 | 24.319 |
| rs141601713 | Gastric cancer | 17 | 45614919 | T/C | 0.051 | -0.002 | 0.102 | 0.986 | 58.185 |
| rs115736925 | Gastric cancer | 17 | 45746683 | T/C | 0.051 | -0.002 | 0.102 | 0.982 | 44.127 |
| rs36043200 | Gastric cancer | 17 | 45629406 | A/G | 0.559 | -0.009 | 0.017 | 0.590 | 34.091 |
| rs4424943 | Gastric cancer | 17 | 45870375 | A/G | 0.171 | -0.021 | 0.022 | 0.345 | 32.154 |
| rs2325750 | Gastric cancer | 17 | 46022330 | A/G | 0.706 | -0.025 | 0.020 | 0.204 | 21.703 |
| rs79100284 | Gastric cancer | 17 | 45848646 | T/C | 0.042 | -0.102 | 0.111 | 0.358 | 22.740 |
| TOP2B |  |  |  |  |  |  |  |  |  |
| rs115802056 | Gastric cancer | 3 | 25680951 | G/A | 0.048 | 0.054 | 0.107 | 0.613 | 19.627 |
| rs1286765 | Gastric cancer | 3 | 25593872 | A/G | 0.540 | 0.032 | 0.017 | 0.053 | 40.569 |
| rs7652621 | Gastric cancer | 3 | 25626444 | C/T | 0.099 | 0.028 | 0.032 | 0.381 | 36.297 |
| rs7624894 | Gastric cancer | 3 | 25642157 | C/T | 0.108 | 0.025 | 0.023 | 0.285 | 36.499 |
| rs1656452 | Gastric cancer | 3 | 25579777 | T/C | 0.216 | 0.024 | 0.019 | 0.205 | 26.984 |
| rs34214973 | Gastric cancer | 3 | 25600836 | G/A | 0.295 | 0.024 | 0.016 | 0.139 | 20.197 |
| rs1286738 | Gastric cancer | 3 | 25614532 | T/C | 0.208 | 0.022 | 0.021 | 0.282 | 35.093 |
| rs12631825 | Gastric cancer | 3 | 25652111 | G/A | 0.317 | 0.000 | 0.017 | 0.988 | 225.873 |
| rs73043444 | Gastric cancer | 3 | 25847265 | A/G | 0.042 | -0.114 | 0.111 | 0.304 | 20.270 |
| rs1561033 | Gastric cancer | 3 | 25664830 | T/C | 0.084 | -0.158 | 0.080 | 0.049 | 55.784 |
| rs143037061 | Gastric cancer | 3 | 25721260 | A/G | 0.044 | -0.263 | 0.137 | 0.055 | 28.751 |
| HMGN2 |  |  |  |  |  |  |  |  |  |
| rs12129981 | Gastric cancer | 1 | 26702214 | A/G | 0.126 | 0.101 | 0.042 | 0.016 | 53.149 |
| rs12128284 | Gastric cancer | 1 | 26695687 | A/G | 0.034 | 0.076 | 0.122 | 0.531 | 91.963 |
| rs6685995 | Gastric cancer | 1 | 26969814 | A/G | 0.029 | 0.075 | 0.133 | 0.572 | 22.515 |
| rs2925523 | Gastric cancer | 1 | 26805169 | T/C | 0.931 | 0.075 | 0.157 | 0.633 | 154.363 |
| rs11809030 | Gastric cancer | 1 | 26988026 | A/G | 0.221 | 0.070 | 0.048 | 0.144 | 27.043 |
| rs55755393 | Gastric cancer | 1 | 26780724 | A/G | 0.211 | 0.069 | 0.047 | 0.146 | 68.605 |
| rs78489761 | Gastric cancer | 1 | 26954296 | G/T | 0.077 | 0.067 | 0.085 | 0.435 | 55.908 |
| rs34774220 | Gastric cancer | 1 | 26903412 | T/C | 0.335 | 0.038 | 0.038 | 0.324 | 109.425 |
| rs12142542 | Gastric cancer | 1 | 26590366 | C/A | 0.034 | 0.022 | 0.123 | 0.859 | 72.831 |
| rs282178 | Gastric cancer | 1 | 26899687 | A/C | 0.238 | 0.018 | 0.055 | 0.741 | 38.848 |
| rs2445637 | Gastric cancer | 1 | 26813366 | C/T | 0.405 | 0.012 | 0.019 | 0.542 | 45.282 |
| rs12122817 | Gastric cancer | 1 | 26690180 | A/G | 0.092 | 0.011 | 0.093 | 0.904 | 39.350 |
| rs4970489 | Gastric cancer | 1 | 26879792 | C/T | 0.734 | 0.008 | 0.018 | 0.654 | 43.930 |
| rs4436378 | Gastric cancer | 1 | 26715232 | A/G | 0.588 | 0.000 | 0.022 | 0.988 | 22.958 |
| rs2316002 | Gastric cancer | 1 | 26854674 | T/C | 0.530 | -0.003 | 0.023 | 0.901 | 84.751 |
| rs17261915 | Gastric cancer | 1 | 26756856 | C/T | 0.215 | -0.016 | 0.022 | 0.452 | 36.442 |
| rs78113009 | Gastric cancer | 1 | 26499036 | G/T | 0.054 | -0.023 | 0.098 | 0.813 | 52.209 |
| rs11581016 | Gastric cancer | 1 | 26878390 | A/G | 0.117 | -0.047 | 0.047 | 0.318 | 47.062 |
| rs6683157 | Gastric cancer | 1 | 26545865 | A/G | 0.157 | -0.055 | 0.024 | 0.024 | 39.775 |
| ARL6IP1 |  |  |  |  |  |  |  |  |  |
| rs137863609 | Gastric cancer | 16 | 18875042 | A/C | 0.034 | 0.232 | 0.226 | 0.303 | 29.527 |
| rs57293127 | Gastric cancer | 16 | 18607586 | A/G | 0.200 | 0.144 | 0.102 | 0.161 | 44.556 |
| rs76937745 | Gastric cancer | 16 | 18831882 | C/T | 0.041 | 0.099 | 0.113 | 0.385 | 34.993 |
| rs139900982 | Gastric cancer | 16 | 18956160 | G/A | 0.031 | 0.091 | 0.132 | 0.493 | 47.759 |
| rs12448824 | Gastric cancer | 16 | 19009904 | A/G | 0.421 | 0.048 | 0.046 | 0.304 | 70.170 |
| rs72777499 | Gastric cancer | 16 | 19087796 | T/C | 0.209 | 0.037 | 0.057 | 0.514 | 76.142 |
| rs12933625 | Gastric cancer | 16 | 18961054 | T/G | 0.598 | 0.023 | 0.017 | 0.172 | 298.097 |
| rs4780773 | Gastric cancer | 16 | 18975564 | A/G | 0.667 | 0.023 | 0.049 | 0.643 | 26.336 |
| rs17731755 | Gastric cancer | 16 | 18865935 | C/T | 0.069 | 0.018 | 0.092 | 0.842 | 58.157 |
| rs11646535 | Gastric cancer | 16 | 18975487 | A/G | 0.398 | 0.017 | 0.049 | 0.727 | 24.524 |
| rs192809799 | Gastric cancer | 16 | 19080794 | A/G | 0.033 | 0.004 | 0.151 | 0.981 | 39.845 |
| rs11640739 | Gastric cancer | 16 | 19029283 | T/C | 0.089 | -0.010 | 0.080 | 0.901 | 28.494 |
| rs36054827 | Gastric cancer | 16 | 18884830 | T/G | 0.324 | -0.013 | 0.022 | 0.564 | 1018.741 |
| rs9635482 | Gastric cancer | 16 | 18787237 | G/A | 0.080 | -0.017 | 0.033 | 0.609 | 75.069 |
| rs12927397 | Gastric cancer | 16 | 18950852 | A/G | 0.106 | -0.019 | 0.030 | 0.523 | 50.575 |
| rs78227835 | Gastric cancer | 16 | 18974506 | A/G | 0.042 | -0.019 | 0.114 | 0.865 | 30.177 |
| rs4637337 | Gastric cancer | 16 | 18784829 | A/G | 0.326 | -0.032 | 0.022 | 0.149 | 949.485 |
| rs66993176 | Gastric cancer | 16 | 18860151 | C/T | 0.061 | -0.041 | 0.097 | 0.673 | 62.041 |
| rs2606429 | Gastric cancer | 16 | 18784153 | T/C | 0.062 | -0.054 | 0.092 | 0.563 | 55.924 |
| rs117506357 | Gastric cancer | 16 | 18873684 | A/G | 0.120 | -0.078 | 0.068 | 0.254 | 203.475 |
| DCUN1D1 |  |  |  |  |  |  |  |  |  |
| rs11922132 | Gastric cancer | 3 | 182490754 | C/T | 0.113 | 0.038 | 0.023 | 0.095 | 30.525 |
| rs141583319 | Gastric cancer | 3 | 182645219 | C/T | 0.042 | 0.007 | 0.113 | 0.954 | 20.271 |
| rs74376726 | Gastric cancer | 3 | 182694981 | A/G | 0.148 | 0.003 | 0.018 | 0.891 | 37.693 |
| rs2971450 | Gastric cancer | 3 | 182567156 | A/G | 0.689 | -0.018 | 0.016 | 0.266 | 46.289 |
| rs1879988 | Gastric cancer | 3 | 182464135 | C/T | 0.648 | -0.019 | 0.017 | 0.264 | 20.392 |
| rs598689 | Gastric cancer | 3 | 182861572 | G/A | 0.863 | -0.020 | 0.019 | 0.299 | 32.513 |
| rs575326 | Gastric cancer | 3 | 182890241 | T/C | 0.899 | -0.022 | 0.026 | 0.383 | 23.703 |
| rs6765551 | Gastric cancer | 3 | 182427687 | A/C | 0.343 | -0.023 | 0.019 | 0.214 | 19.529 |
| rs73178986 | Gastric cancer | 3 | 182815540 | A/G | 0.106 | -0.025 | 0.072 | 0.729 | 21.067 |
| rs658672 | Gastric cancer | 3 | 182877292 | C/T | 0.752 | -0.025 | 0.016 | 0.117 | 22.768 |
| rs11715991 | Gastric cancer | 3 | 182695762 | A/G | 0.617 | -0.026 | 0.022 | 0.239 | 74.733 |
| rs67343033 | Gastric cancer | 3 | 182668445 | T/C | 0.087 | -0.041 | 0.034 | 0.225 | 59.044 |
| rs623712 | Gastric cancer | 3 | 182835666 | C/T | 0.968 | -0.116 | 0.127 | 0.363 | 22.128 |
| CENPJ |  |  |  |  |  |  |  |  |  |
| rs143639540 | Gastric cancer | 13 | 25426348 | T/C | 0.020 | -0.062 | 0.161 | 0.700 | 55.465 |
| rs117213023 | Gastric cancer | 13 | 25566695 | A/G | 0.023 | -0.189 | 0.152 | 0.214 | 41.330 |
| ZNF148 |  |  |  |  |  |  |  |  |  |
| rs73195466 | Gastric cancer | 3 | 125040698 | G/A | 0.025 | 0.105 | 0.146 | 0.472 | 35.870 |
| rs1455051 | Gastric cancer | 3 | 124999627 | T/C | 0.667 | 0.034 | 0.016 | 0.033 | 242.568 |
| rs4679388 | Gastric cancer | 3 | 125075923 | A/C | 0.334 | 0.029 | 0.016 | 0.070 | 24.156 |
| rs6799398 | Gastric cancer | 3 | 125141827 | G/A | 0.424 | 0.022 | 0.017 | 0.194 | 23.685 |
| rs2333218 | Gastric cancer | 3 | 125127132 | C/T | 0.382 | 0.013 | 0.022 | 0.545 | 45.188 |
| rs4431054 | Gastric cancer | 3 | 125353202 | G/A | 0.057 | 0.011 | 0.029 | 0.705 | 22.951 |
| rs73195448 | Gastric cancer | 3 | 124994033 | G/A | 0.079 | 0.001 | 0.084 | 0.991 | 68.892 |
| rs518109 | Gastric cancer | 3 | 124894687 | C/T | 0.460 | -0.015 | 0.016 | 0.353 | 33.363 |
| rs11922173 | Gastric cancer | 3 | 124937570 | C/T | 0.380 | -0.021 | 0.018 | 0.239 | 36.246 |
| rs74487985 | Gastric cancer | 3 | 125174856 | G/A | 0.065 | -0.155 | 0.113 | 0.169 | 20.649 |
| ZNF33A |  |  |  |  |  |  |  |  |  |
| rs148589061 | Gastric cancer | 10 | 38151413 | A/G | 0.023 | 0.215 | 0.153 | 0.160 | 21.178 |
| rs138867977 | Gastric cancer | 10 | 38592924 | T/G | 0.058 | 0.091 | 0.096 | 0.341 | 63.737 |
| rs1819389 | Gastric cancer | 10 | 38440389 | T/C | 0.058 | 0.082 | 0.096 | 0.392 | 64.474 |
| rs2254918 | Gastric cancer | 10 | 38249547 | G/A | 0.853 | 0.063 | 0.076 | 0.408 | 20.445 |
| rs147495805 | Gastric cancer | 10 | 38090261 | A/G | 0.092 | 0.055 | 0.078 | 0.481 | 55.904 |
| rs1625270 | Gastric cancer | 10 | 38192360 | C/T | 0.082 | 0.007 | 0.081 | 0.930 | 48.036 |
| rs145766788 | Gastric cancer | 10 | 38293929 | T/C | 0.082 | 0.004 | 0.081 | 0.960 | 52.873 |
| PTP4A2 |  |  |  |  |  |  |  |  |  |
| rs61099507 | Gastric cancer | 1 | 32313196 | G/A | 0.195 | 0.014 | 0.020 | 0.485 | 24.068 |
| rs1536131 | Gastric cancer | 1 | 32314400 | G/A | 0.607 | -0.021 | 0.016 | 0.205 | 34.133 |
| HNRNPA1 |  |  |  |  |  |  |  |  |  |
| rs35979828 | Gastric cancer | 12 | 54685880 | T/C | 0.056 | 0.004 | 0.098 | 0.970 | 76.955 |
| SET |  |  |  |  |  |  |  |  |  |
| rs3750321 | Gastric cancer | 9 | 131465284 | T/G | 0.574 | 0.003 | 0.020 | 0.896 | 25.004 |
